# Selective Integrin Ligands Promote Cell Internalization of the Antineoplastic Agent Fluorouracil

**DOI:** 10.1021/acsptsci.1c00094

**Published:** 2021-09-02

**Authors:** Monica Baiula, Martina Cirillo, Giulia Martelli, Valentina Giraldi, Elisa Gasparini, Alessandro Claudio Anelli, Santi Mario Spampinato, Daria Giacomini

**Affiliations:** ‡ Department of Pharmacy and Biotechnology, University of Bologna, Via Irnerio, 48, 40126, Bologna, Italy; † Department of Chemistry “G. Ciamician”, University of Bologna, Via Selmi 2, 40126 Bologna, Italy

**Keywords:** Lactams, integrins, cell adhesion, agonist, internalization, 5-fluorouracil

## Abstract

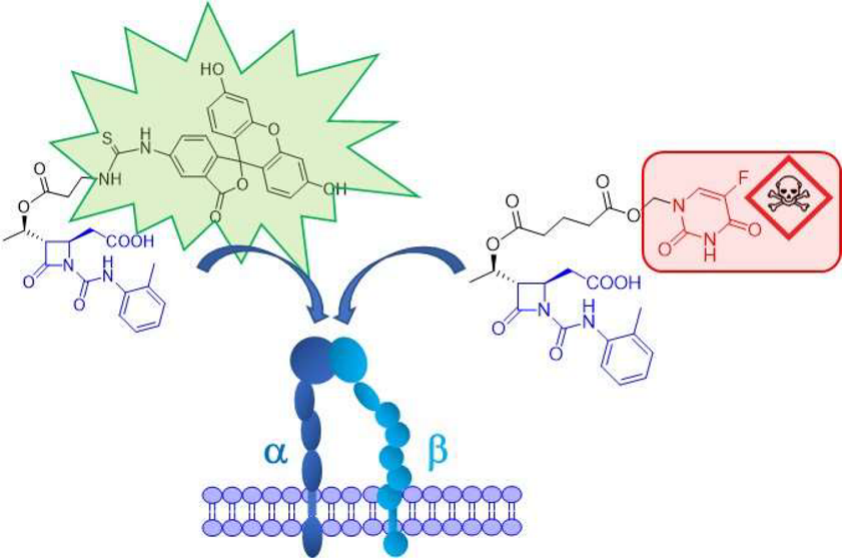

Drug conjugates consisting of an antineoplastic drug and a targeting receptor ligand could be effective to overcome the heavy side effects of unselective anticancer agents. To address this need, we report here the results of a project aimed to study agonist and antagonist integrin ligands as targeting head of molecular cargoes for the selective delivery of 5-fluorouracil (5-FU) to cancer or noncancer cells. Initially, two fluorescent β-lactam-based integrin ligands were synthesized and tested for an effective and selective internalization mediated by α_4_β_1_ or α_5_β_1_ integrins in Jurkat and K562 cells, respectively. No cellular uptake was observed for both fluorescent compounds in HEK293 noncancerous control cells. Afterward, three conjugates composed of the β-lactam-based integrin ligand, suitable linkers, and 5-FU were realized. The best compound E, acting as α_5_β_1_ integrin agonist, is able to selectively deliver 5-FU into tumor cells, successfully leading to cancer cell death.

Targeted drug delivery can be an effective strategy to increase the bioavailability of therapeutics, specifically to cancer tissue, to decrease the heavy side effects (nonspecific delivery to healthy tissue), and to improve clinical outcomes.^1^ In particular, it has been recognized how molecular interactions between receptors and ligands that control cell-to-cell communications may represent an effective target.^2^ Considerable progresses in tumor-targeting strategies have been achieved with antineoplastic drug conjugates as delivery systems that consist of a tumor-targeting group and an antineoplastic drug, connected by a linker. In this context, integrins are peculiar receptors because they activate intracellular signaling pathways to regulate cell growth, survival, migration, invasion, and angiogenesis.^3^

Integrins are overexpressed in many types of cancer cells, and they have been implicated in mediating several hallmarks of cancer, including cancer cell proliferation, dormancy, survival, stemness, metabolic adaptation, and metastatic niche.^4−7^ In particular, α_5_β_1_ integrin plays a predominant role in tumor-induced angiogenesis, migration, and invasion of cancer cells; it is aberrantly upregulated in various types of cancers; and its overexpression is correlated with poor prognosis.^8,9^ The α_v_β_6_ integrin is an epithelial cell-restricted receptor and is expressed in malignant cells but not in normal epithelium;^10,11^ it is involved in tumor formation and progression by modulating the expression of metalloproteinase enzymes.^12^ Despite its important role in inflammation and immunity, α_4_β_1_ integrin is also expressed on several types of tumor cells and contributes to migration and metastasis, tumor angiogenesis, and development of drug resistance.^6,9,13^

In addition, integrins can be internalized upon specific ligand binding^14−16^ and therefore they may be used as shuttles to selectively release the antineoplastic drug only inside integrin expressing cancer cells.^17^ Chemotherapy is considered the standard of care for several locally advanced cancers. Cytotoxic drugs have been largely employed in this setting, with the pyrimidine analogue 5-fluorouracil (5-FU) and cisplatin being the most often employed.^18^ 5-FU is a well-known and widely used antineoplastic drug for the treatment of different types of cancers. It acts as an antimetabolite by inhibiting essential biosynthetic processes and by being incorporated into RNA and DNA, disrupting their normal function.^19,20^ These effects can induce, among others, cell cycle arrest^21,22^ and promote apoptosis triggered by p53.^23,24^ In order to obtain a better clinical use of antineoplastic drugs, many integrin-targeted peptide- and peptidomimetic-drug conjugates have been developed and investigated.^25−29^

In this study, 5-FU was used as a model drug for the development of an effective anticancer drug delivery therapy, exploiting its conjugation to novel and selective integrin ligands for promoting an active tumor targeting.^30^ Conjugation of anticancer drugs with integrin-specific ligands may in fact lead to higher selectivity toward cancer cells and to payload accumulation within tumor cells through integrin trafficking.^17^ From a pharmacological point of view, ligands can be classified on the basis of their action at the receptor: agonists are able to bind the receptor mimicking the action of the endogenous agonists, thus inducing intracellular signaling activation and in some cases receptor internalization.^31^ On the contrary, antagonists bind to the receptor blocking its interaction with endogenous agonists without inducing signal transduction or receptor internalization. Therefore, we have hypothesized that integrin agonists, conjugated with anticancer drugs, could be exploited as a targeting unit to promote selective drug internalization only into cancer cells.

Our previous studies provided a series of novel molecules designed to selectively target different integrins, mainly RGD-binding or leukocyte classes,^32−34^ which are capable of modulating integrin-mediated cellular processes. Some ligands behave as agonists promoting cell adhesion and intracellular signaling, while others, acting as integrin antagonists, are able to inhibit integrin-dependent cell functions. As a proof of concept on the applicability of agonist ligands, some compounds with a β-lactam scaffold were chosen to functionalize electrospun polylactic acid nanofibers or strontium-substituted hydroxyapatite.^35,36^ The new functionalized biomaterials with incorporated agonist ligands showed enhanced properties in adhesion of human mesenchymal stem cells (hMSC) with promising applications in tissue regeneration.^37^

In the present work, starting from the previously studied compound **A** as a selective agonist for α_4_β_1_ integrins,^32^ we first designed and realized two fluorescent compounds **B** and **C** to get evidence on ligands internalization (Figure 1). Then, the chemotherapeutic agent 5-FU, chosen as model drug, was conjugated to the integrin ligand **A** by means of different linkers and the so-obtained new conjugates **D**, **E**, and **F** were studied in cell-based assays in order to ascertain their activity and selectivity against tumor cells (Figure 1).

**Figure 1:**
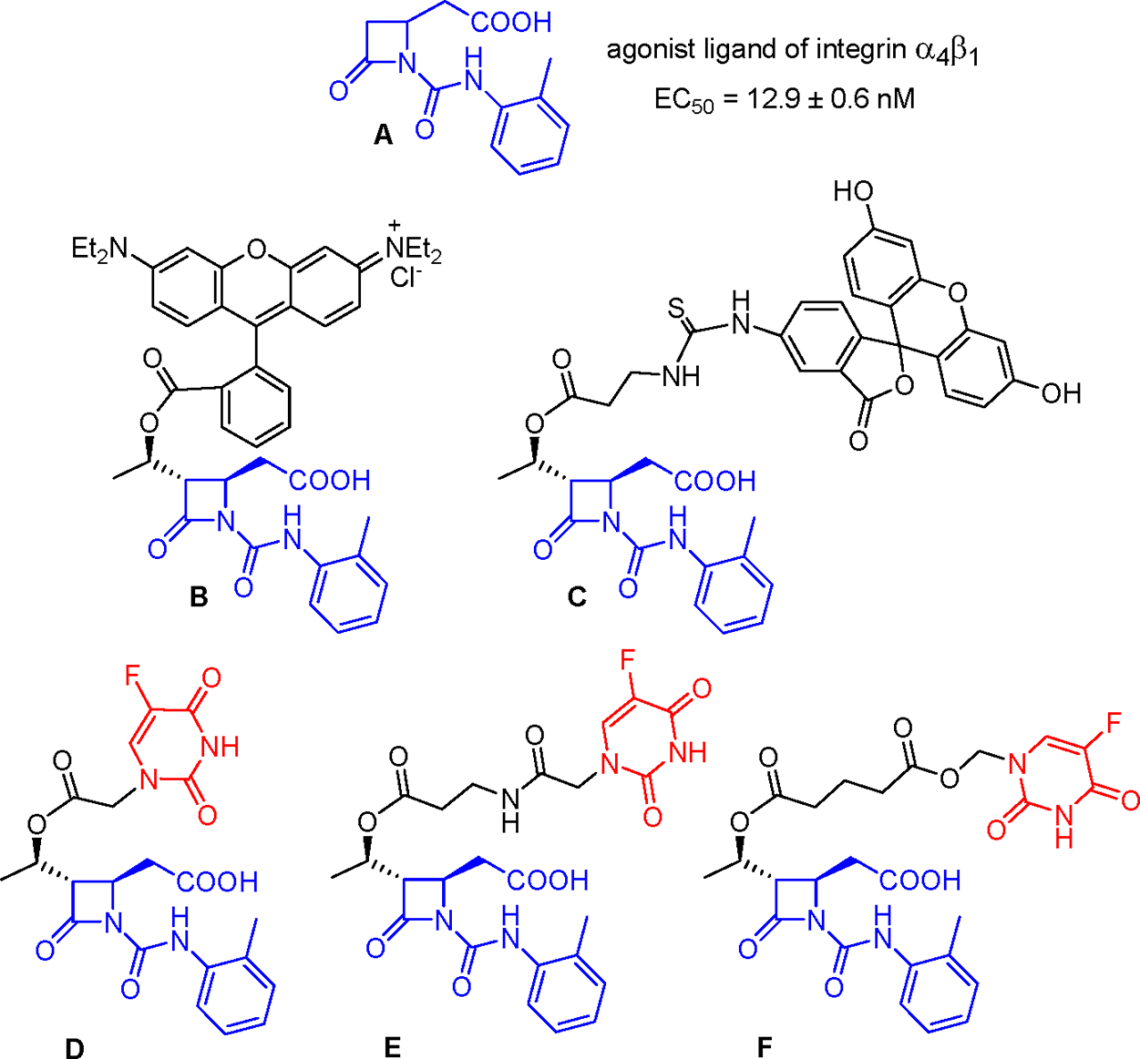
A series of β-lactam compounds evaluated in this study. Compound **A** is the reference compound as model of integrin agonist ligand; **B** and **C** are new fluorescent compounds for internalization analyses; **D**, **E**, and **F** are new 5-FU-conjugates designed to evaluate the selectivity of the anticancer effect.

## Results

### Chemistry

**Scheme 1:**
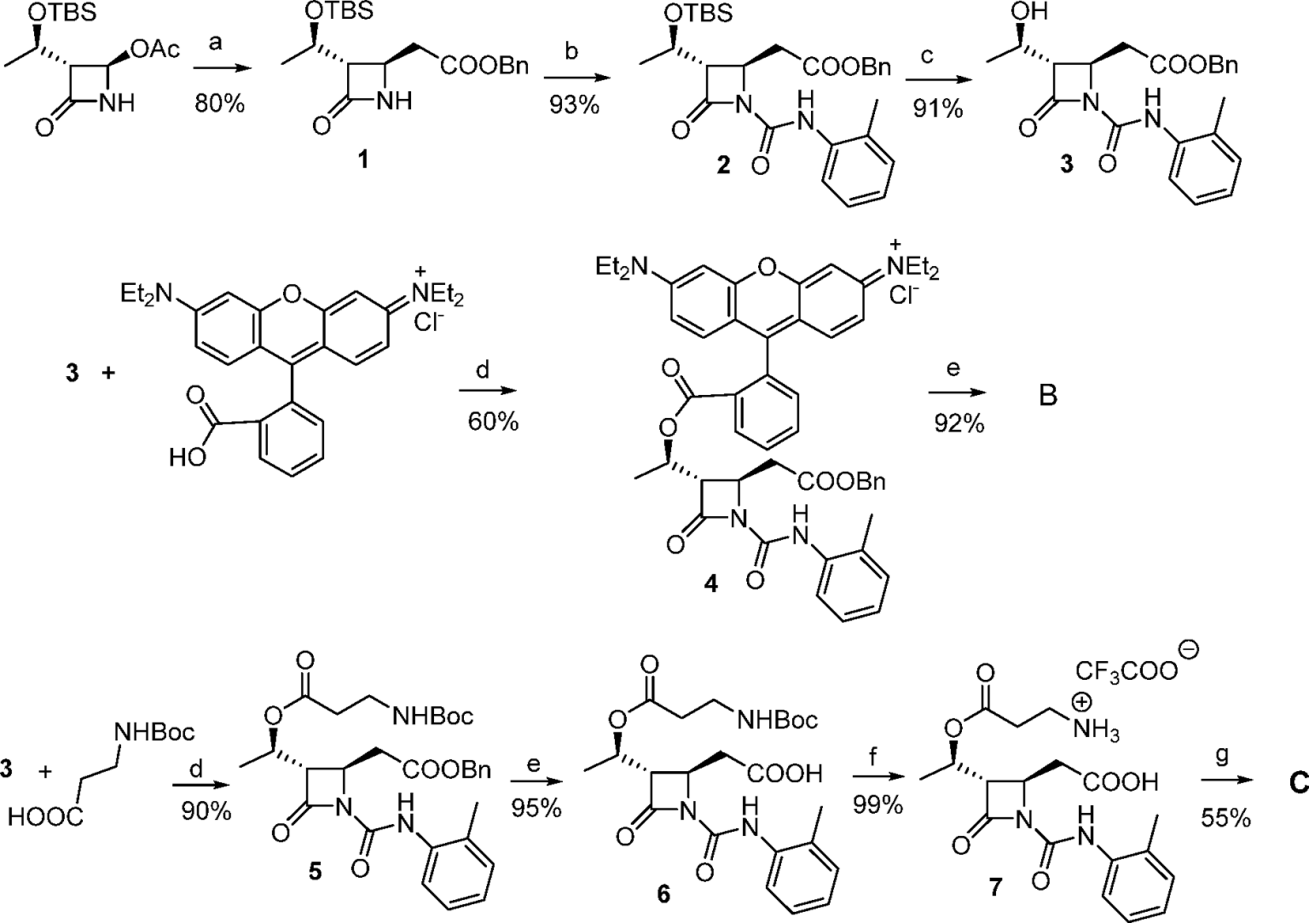
Synthesis of Fluorescent Compounds **B** and **C** Reagents and conditions: (a) Zn, TMSCl, benzylbromoacetate, THF, 0 °C then rt, 3 h; (b) *o*-tolylisocyanate, TEA, CH_2_Cl_2_, rt, 16 h; (c) BF_3_·OEt_2_, CH_3_CN, 0 °C then rt, 2 h; (d) DCC, TEA, DMAP, Rhodamine B or *N*-Boc-beta alanine, CH_2_Cl_2_, 0 °C then rt, 24 h; (e) H_2_, Pd/C (10%), THF/CH_3_OH 1:1, rt, 2 h; (f) TFA, CH_2_Cl_2_, 0 °C then rt, 16 h; (g) fluorescein isothiocyanate, TEA, CH_2_Cl_2_, rt, 4 h. Yields % refer to isolated compounds.

Compound **A** was chosen as a model for the design of the new ligands that should keep a carboxylic acid terminal on the C4 position of the β-lactam scaffold to target the integrin metal ion-dependent adhesion site (MIDAS), and an *o*-tolyl-urea moiety on the β-lactam nitrogen for a selective activation of α_4_β_1_ integrins. To trace the integrin-mediated internalization of the compounds into the cell, a fluorescent tag was introduced on the model compound **A** that was suitably modified. Accordingly, tags were anchored on the C3 side chain of a 3-hydroxyethyl-β-lactam, and Rhodamine B or fluorescein isothiocyanate (FITC) was chosen to obtain compounds **B** and **C**, respectively (Scheme 1).

The synthesis of fluorescent compounds **B** and **C** started from a nucleophilic substitution reaction on the C-4 position of the commercially available (2*R*,3*R*)-3-((*R*)-1-((*t*-butyl dimethylsilyl)oxy) ethyl)-4-oxoazetidin-2-yl acetate, with a Reformatsky reagent obtained in turn from benzyl bromoacetate and zinc preactivated with *t*-butyldimethylsilyl chloride (Scheme 1). The substitution of the 4-acetoxy group occurred with a complete control of the stereoselectivity obtaining exclusively the *trans* diastereoisomer **1**.^38^ Compound **1** was then acylated on the β-lactam nitrogen atom with the commercially available *o*-tolylisocyanate to give **2**, in order to get the specific *o*-tolylureidic residue necessary for modulating the affinity toward the integrin receptor. The *t*-butyldimethylsilyl group on the C-3 side chain was then removed with BF_3_·OEt_2_ as Lewis acid affording alcohol **3** in good yields.

For the synthesis of compound **B**, the hydroxyl group in compound **3** was exploited for inserting Rhodamine B by DCC and DMAP-mediated esterification reaction to give intermediate **4**. The final deprotection of benzyl ester on the C-4 side chain catalyzed by Pd/C yielded the free carboxylic acid needed for integrin recognition at the MIDAS.

To obtain compound **C**, in order to have a free amine group for the insertion of the fluorescein fluorophore, alcohol **3** was subjected to a DCC-mediated esterification with the commercial *N*-Boc-β-alanine, obtaining compound **5** in excellent yields. Following hydrogenolysis for benzyl ester deprotection and Boc removal, compound **7** was achieved in quantitative yields. TEA-mediated reaction of **7** with FITC gave the target compound **C** in 55% yield after flash chromatography.

**Scheme 2:**
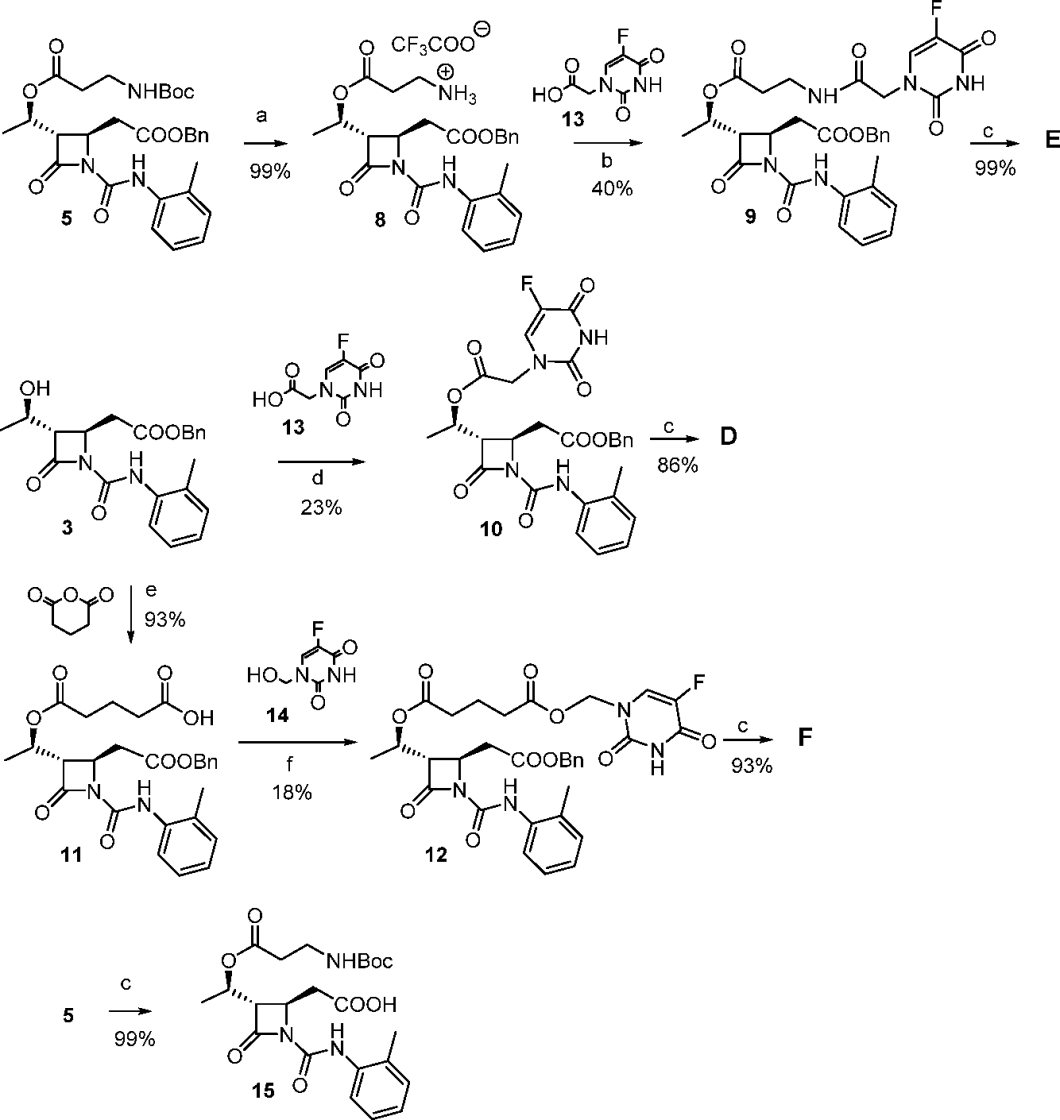
Synthesis of 5-FU Conjugate Compounds **D**, **E**, and **F** Reagents and conditions: (a) TFA, CH_2_Cl_2_, 0 °C then rt, 5 h; (b) TEA, CH_2_Cl_2_, HOBt, EDC, DMF, 0 °C then rt, 18 h; (c) H_2_, Pd/C (10%), THF/CH_3_OH 1:1, rt, 2 h; (d) DCC, DMAP, CH_3_CN, DMF, rt, 48 h; (e) TEA, DMAP, DCM, rt, 18 h; (f) EDC, DMAP, CH_3_CN, rt, 18 h. Yields % refer to isolated compounds.

To conjugate 5-FU to the selected β-lactam scaffold, we designed three different anchoring systems: a short ester linkage to give compound **D**, a longer diester generated from glutaric anhydride to obtain **F**, and an ester–amide linker derived from beta-alanine to get **E** (Scheme 2). The linker would be responsible to give enough stability to the cargo to reach the target and to successfully release the drug at the tumor cells.^39^ We chose innocent hydrocarbon chains which did not substantially increase the molecular weight of the cargo, did not give any interference with the recognition process, and were differently connected to the drug with an ester or amide group, in order to obtain a good release of the free drug.

Synthesis of compound **D** comprised a DCC/DMAP-mediated esterification between alcohol **3** and the fluorouracil acid **13**, prepared as previously reported.^40^ The final hydrogenolysis of the benzylester group on compound **10** gave **D** in good yields (Scheme 2).

Compound **E** was obtained from intermediate **5**, which was subjected to Boc deprotection with TFA to give **8**. A coupling reaction with **13** mediated by TEA, HOBt, and EDC gained **9**, and a final hydrogenolysis quantitatively yielded compound **E** (Scheme 2).

In order to obtain compound **F**, a coupling between alcohol **3** and glutaric anhydride under mild conditions^41^ gained acid **11** in excellent yields, without the need of purification. The *N*-hydroxymethylene-5-fluorouracil **14**, obtained as reported in the literature,^42^ was then esterified with **11** in the presence of EDC and DMAP. Finally, hydrogenolysis of the benzyl ester on compound **12** gained target product **F** with the free carboxylic acid group required for integrin recognition at MIDAS.

In addition, starting from the intermediate **5**, product **15** was obtained by hydrogenolysis, and it was used as reference compound of a β-lactam analogue without the cytotoxic portion.

### Stability Assays

**Figure 2:**
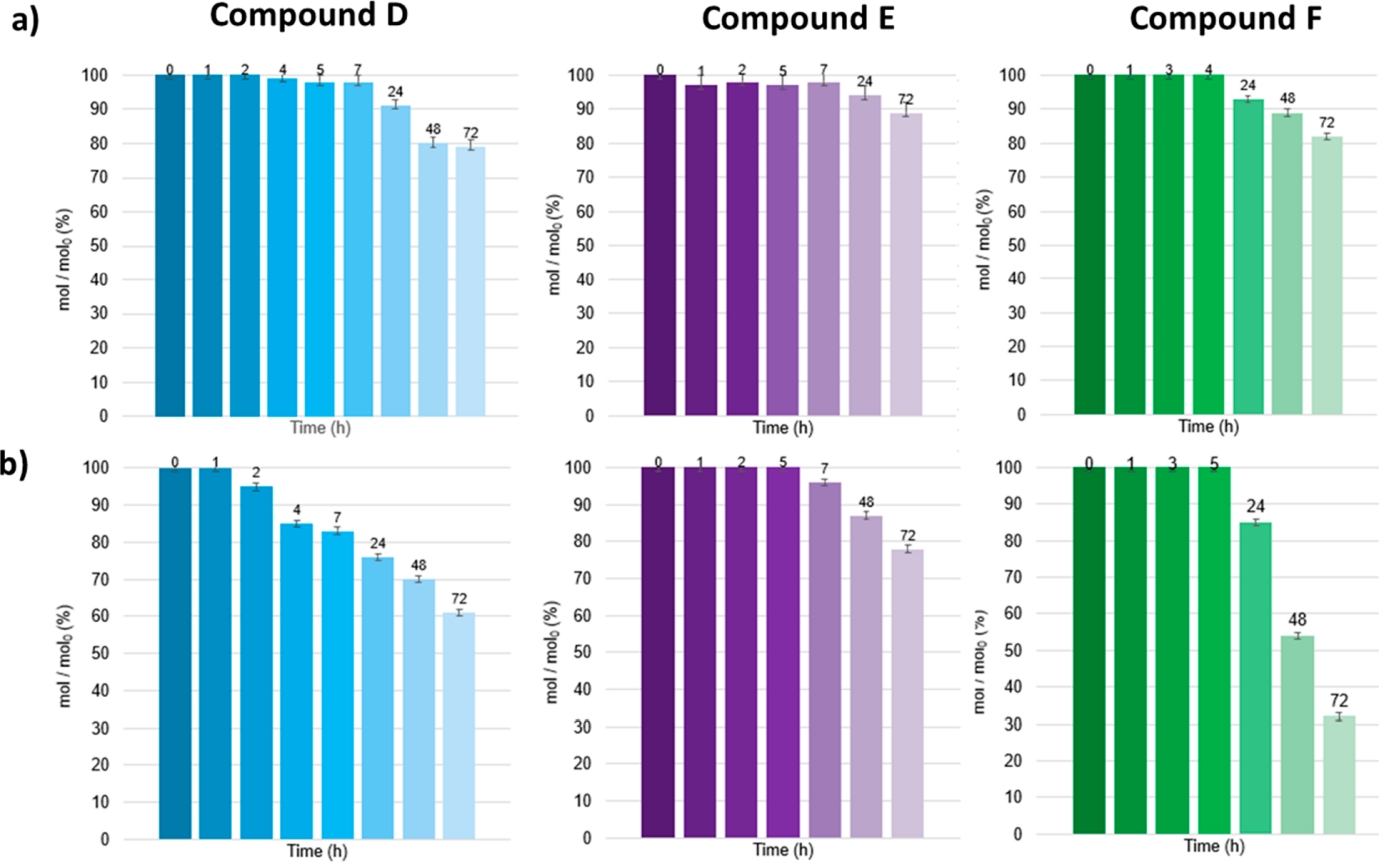
Stability studies for compounds **D** (blue), **E** (violet) and **F** (green); panel (a) stabilities in phosphate buffer solution (PBS) 0.1 M pH = 7.4; panel (b) stabilities in fetal bovine serum (FBS). The amount of intact compounds is reported as mol % respect to mol % at time = 0 (mol_0_).

The stability of the three new 5-FU conjugates **D**, **E**, and **F** was tested in Phosphate Buffer Solution (PBS) 0.1 M (pH = 7.4) and in Fetal Bovine Serum (FBS) as models for physiological conditions and evaluated by HPLC-UV analysis (Supporting Information). The compounds (1 mg/mL) were dissolved in PBS or FBS and incubated at 30 °C in thermostat. Aliquots were taken at different time points from 0 to 72 h, since the analysis of apoptosis induction by compounds **D**–**F** in cells is measured after 72 h exposure. The results are summarized in Figure 2 and reported as mol % of intact compound respect to mol % at the initial time (mol_0_). Compound **E** showed a good stability in both PBS and FBS with an 89 and 78 mol/mol_0_ (%) recovery of the intact conjugate after 72 h, respectively. Compounds **D** and **F** are rather stable in PBS, whereas in FBS the stability underwent a sudden decrease: after 72 h the intact compounds **D** and **F** were recovered in 60 and 30%, respectively. In order to recognize possible decomposition products in samples of **D** and **F** in FBS, HPLC-MS analyses have been conducted (Supporting Information). Just after 2 h, the test solutions of **D** in FBS showed the formation of the carboxylic acid of 3′-hydroxy-β-lactam due to ester hydrolysis. The test solution of compound **F** in FBS after 24 h showed the formation of the β-lactam-glutaryl acid released by hydrolysis of the aminal group on 5-FU (see Supporting Information). Compounds **E** and **F** are quite stable also under slightly acidic conditions (PBS, 0.1 M, pH = 6), which could mimic a lowered pH of the tumor cell environment (see Supporting Information).^43,44^

### Cell Adhesion Assays

To investigate the ability of new fluorescent compounds **B** and **C** and 5-FU-conjugates **D**, **E**, and **F** to modulate integrin-mediated cell adhesion in comparison with the parental agonist **A**, we employed cell adhesion assays using Jurkat E6.1 cells (mainly expressing α_4_β_1_ integrin),^32^ K562 cells (mainly expressing α_5_β_1_ integrin),^45^ and HT-29 cells (mainly expressing α_v_β_6_ integrin).^46^ All the three integrins evaluated in the present study (α_4_β_1_, α_v_β_6_, and α_5_β_1_) are expressed in several types of cancer cells and their expression, as those of other integrins, has been correlated with metastasis and poor patient prognosis.^47^ Specifically, both α_5_β_1_ and α_v_β_6_ integrins are known to increase tumor progression and cancer invasion and to mediate resistance to radiotherapy,^48^ whereas α_4_β_1_ is involved in cancer cell ability to invade basement membranes and metastasize.^49^ In addition, β_1_ integrins mediate drug resistance and stimulate metastasis of several different tumor types.^50,51^ Cell adhesion results are summarized in Table 1.

**Table 1:** Effects of 5-FU-Conjugate Compounds **B**–**F** on α_5_β_1_, α_v_β_6_, or α_4_β_1_ Integrin-Mediated Cell Adhesion^a^–^c^

		K562/FN	Jurkat/VCAM-1	HT-29/FN
entry	compound	α_5_β_1_	α_4_β_1_	α_v_β_6_
1	**A**	>100^d^	0.0129 ± 0.0006^d^ agonist	>100^d^
2	**B**	0.332 ± 0.047 agonist	0.549 ± 0.066 agonist	>100
3	**C**	11.1 ± 1.7 agonist	41.1 ± 7.3 agonist	>100
4	**D**	0.717 ± 0.070 agonist	0.372 ± 0.052 agonist	>100
5	**E**	1.30 ± 0.31 agonist	2.99 ± 0.41 antagonist	>100
6	**F**	0.058 ± 0.006 antagonist	2.36 ± 0.23 antagonist	>100
7	**15**	0.014 ± 0.004 antagonist	>100	>100

a Data are presented as EC_50_ for agonists and as IC_50_ for antagonists (μM).

b Cell adhesion mediated by α_5_β_1_ for K562 cell adhesion to FN, by α_v_β_6_ for HT-29 cell adhesion to FN and by α_4_β_1_ evaluating Jurkat cell adhesion to VCAM-1.

c Values represent the mean ± SD; *n* = 3.

d Data of parental compound **A** were already published, see ref (32).

Integrin agonists are considered those compounds able to promote cell adhesion to fibronectin or VCAM-1; conversely, antagonists are defined as compounds capable of inhibiting cell adhesion to fibronectin or VCAM-1 in a concentration-dependent manner.

Parental compound **A** has been analyzed in a previous study,^32^ and it showed a potent and selective activity as agonist toward α_4_β_1_ integrin; moreover, it was completely inactive toward all the other integrins investigated (α_v_β_3_, α_v_β_5_, α_v_β_6_, α_IIb_β_3_, α_L_β_2_). For comparison purposes, cell adhesion data of compound **A** on α_4_β_1_, α_v_β_6_, and α_5_β_1_ integrins have been added to Table 1 (entry 1). Both fluorescent compounds **B** and **C** maintained an agonist behavior in cell adhesion assays involving α_4_β_1_, even with a lower potency compared with parental compound **A**, and, unexpectedly, were able to switch on agonism toward α_5_β_1_ integrin (Table 1, entries 2 and 3). In particular, integrin agonist-FITC-conjugated **C** was less potent than Rhodamine B-conjugate compound **B** when employed in cell adhesion assays. Regarding 5-FU-integrin ligand-conjugates, compound **D** behaved as a less effective agonist in cell adhesion assays on α_4_β_1_ integrin compared to parental compound **A**, albeit it maintained an interesting activity in the submicromolar range (EC_50_: 0.372 ± 0.052 μM, Table 1 entry 4). In addition, compound **D** acted as an agonist also in α_5_β_1_ integrin-mediated cell adhesion assay (Table 1, entry 4), conversely to parental compound **A**, which was reported to be highly selective for α_4_β_1_.^32^ The elongation of the anchoring system onto the β-lactam scaffold with an ester–amide linker, as in compound **E**, induced a reduction in the potency toward α_5_β_1_ integrin if compared to compound **D** but still maintained the agonist behavior. On the contrary, compound **E** was able to reduce α_4_β_1_-mediated cell adhesion with potency in the micromolar range (Table 1, entry 5). Compound **F**, which bears a long diester as the anchoring system, showed potency and behavior similar to compound **E** toward α_4_β_1_ integrin, acting as an antagonist, while it demonstrated an opposite and more potent activity toward α_5_β_1_ compared with agonists **D** and **E** (Table 1, entries 4, 5, and 6). The reference compound **15** showed a selective potency as antagonist against α_5_β_1_ resembling some β-lactam-based compounds with a carboxylic acid side chain as recently reported.^34^ All the new compounds tested were inactive toward α_v_β_6_ integrin (Table 1). Overall, cell adhesion assays showed that 5-FU-conjugates, when compared with model compound **A**, retain the ability to modulate cell adhesion toward α_4_β_1_ integrin but with a reduced potency and with opposite activity for compounds **E** and **F**. Moreover, both fluorescent and 5-FU-conjugated compounds acquired an interesting activity toward α_5_β_1_ integrin, as agonists for **B**–**E**, and as antagonist in case of **F**.

### Cellular Uptake

In order to determine the capacity of internalization of the novel integrin ligands, two fluorescent compounds **B** and **C** were synthesized, and the extent of their internalization into cancer cells, expressing α_4_β_1_ or α_5_β_1_ integrins (Jurkat and K562, respectively), or noncancer cells, expressing only β_1_ integrin subunit (HEK293), was quantified by flow cytometry.

**Figure 3:**
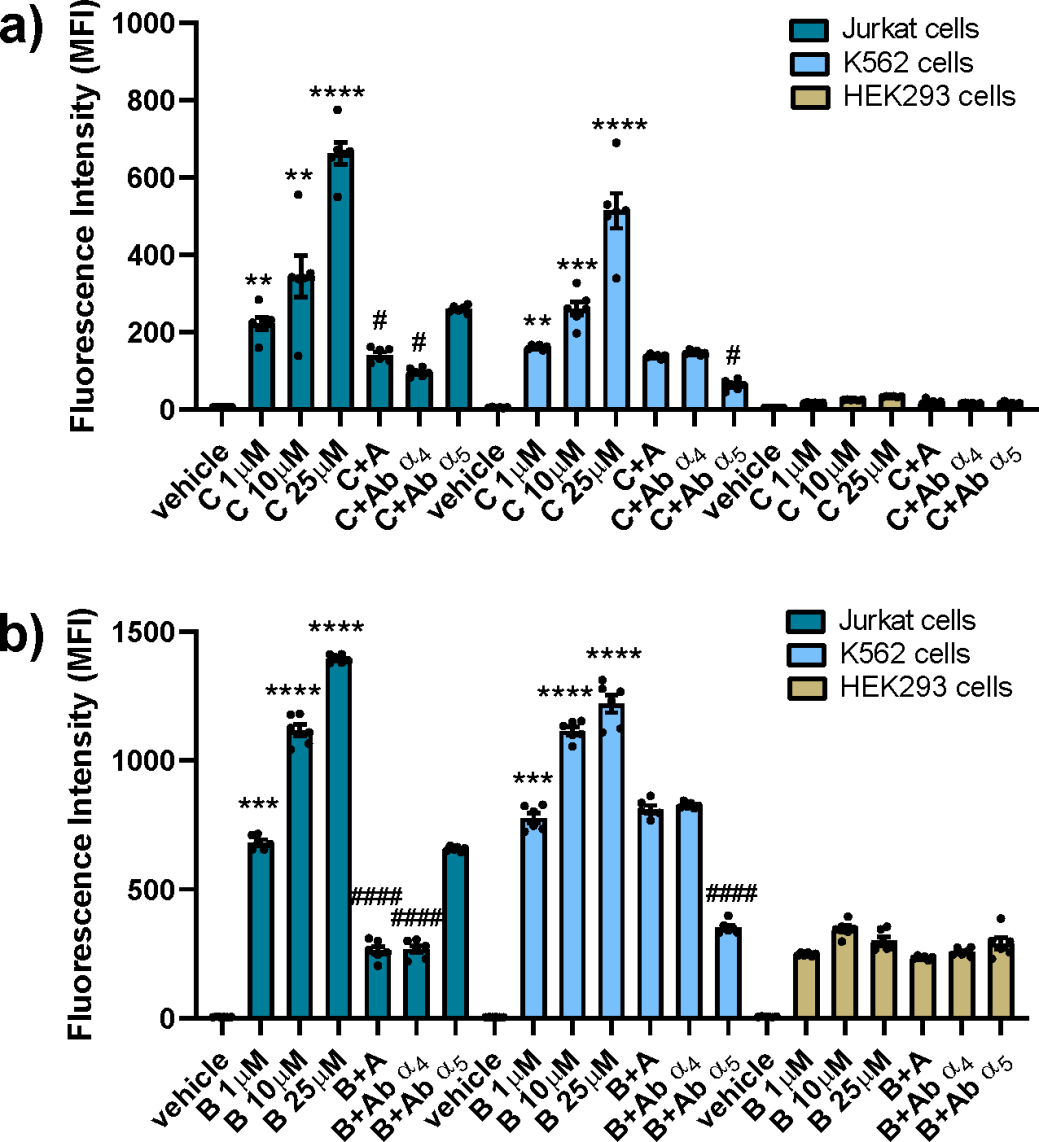
Cellular uptake of integrin agonist-FITC-conjugated **C** (panel a) and Rhodamine B-conjugate **B** (panel b) by Jurkat, K562, and HEK293 cells. Cells were incubated with fluorescent conjugates (1–10–25 μM) or medium containing the vehicle alone (vehicle) for 1 h. To demonstrate integrin involvement, cells were pretreated with anti-α_4_ (10 μg/mL) or anti-α_5_ (10 μg/mL) antibody or α_4_β_1_ selective agonist **A** (100 μM) for 30 min, before the addition of the fluorescent compound (1 μM). The fluorescence intensity of the cells (MFI: mean fluorescence intensity, arbitrary units) corresponds to fluorescent conjugates intracellular uptake and was quantified by flow cytometry. Values are mean ± SD from three independent experiments conducted in triplicate. **p < 0.01; ***p < 0.001; ****p < 0.0001 vs vehicle; #*p* < 0.05, ####*p* < 0.0001 vs 1 μM (Newman-Keuls test after ANOVA).

Fluorescent-FITC-conjugated **C**, which behaves as an integrin agonist for both α_4_β_1_ and α_5_β_1_, is highly internalized in a concentration-dependent manner, both in Jurkat and in K562 cells (Figure 3, panel a). In Jurkat cells, cellular uptake of compound **C** was prevented by pretreatment with agonist **A** or an antibody anti-α_4_ integrin. The blockade of α_5_ integrin with a specific antibody was however ineffective in reducing the internalization of fluorescent conjugate **C** in Jurkat cells. These data suggest that compound **C** internalization in Jurkat cells is α_4_β_1_ integrin-dependent. In K562 cells, intracellular uptake of compound **C** was mediated by α_5_β_1_ integrin, as demonstrated by a strong reduction of internalization induced by pretreatment with an antibody anti-α_5_. Superimposable results were obtained for compound **B** (Figure 3, panel b): it was internalized in a concentration-dependent manner in both Jurkat and K562 cancer cells, and its intracellular uptake was mediated by α_4_ integrin in Jurkat cells and by α_5_ integrin in K562 cells. This behavior of **B** and **C** was also confirmed by qualitative confocal microscopy analysis of the internalization in HEK293 cells transfected with α_4_ or α_5_ integrin subunit, as shown in the Supporting Information Figure S1. No cellular uptake was observed for both compounds **C** and **B** in nontransfected HEK293 noncancer cells (Figure 3, panel a and b, respectively). Altogether, these results demonstrated that fluorescent-integrin ligand conjugates displayed internalization properties required to deliver cytotoxic drugs into cancer cells in an integrin-selective manner.

### Apoptosis Assays

The in vitro activity of 5-FU conjugates **D**, **E**, and **F** was evaluated by apoptosis assays in Jurkat, K562 and HEK293 cells. Previous studies^52,53^ have demonstrated that Jurkat cells present mutations in BAX and TP53 genes, leading to the lack of these proteins or to the production of a truncated isoform, respectively. Although these alterations impair crucial components of apoptotic process, it has been shown that 5-FU is able to trigger apoptosis in a time- and concentration-dependent manner in Jurkat cells.^53^ HEK293 cells were employed as noncancerous control cells; moreover, this cell line does not express α_4_ nor α_5_ integrin subunit, whereas it endogenously expresses the integrin subunit β_1_.^14,32,54^

Jurkat, K562, and HEK293 cell lines were exposed to 5-FU; reference compounds **A**, **13**, **15**. and the three 5-FU-conjugates **D**, **E**, and **F** (10–50–100 μM) for 72 h. Apoptosis was evaluated by Annexin V assay as described in the Materials and Methods.

**Figure 4:**
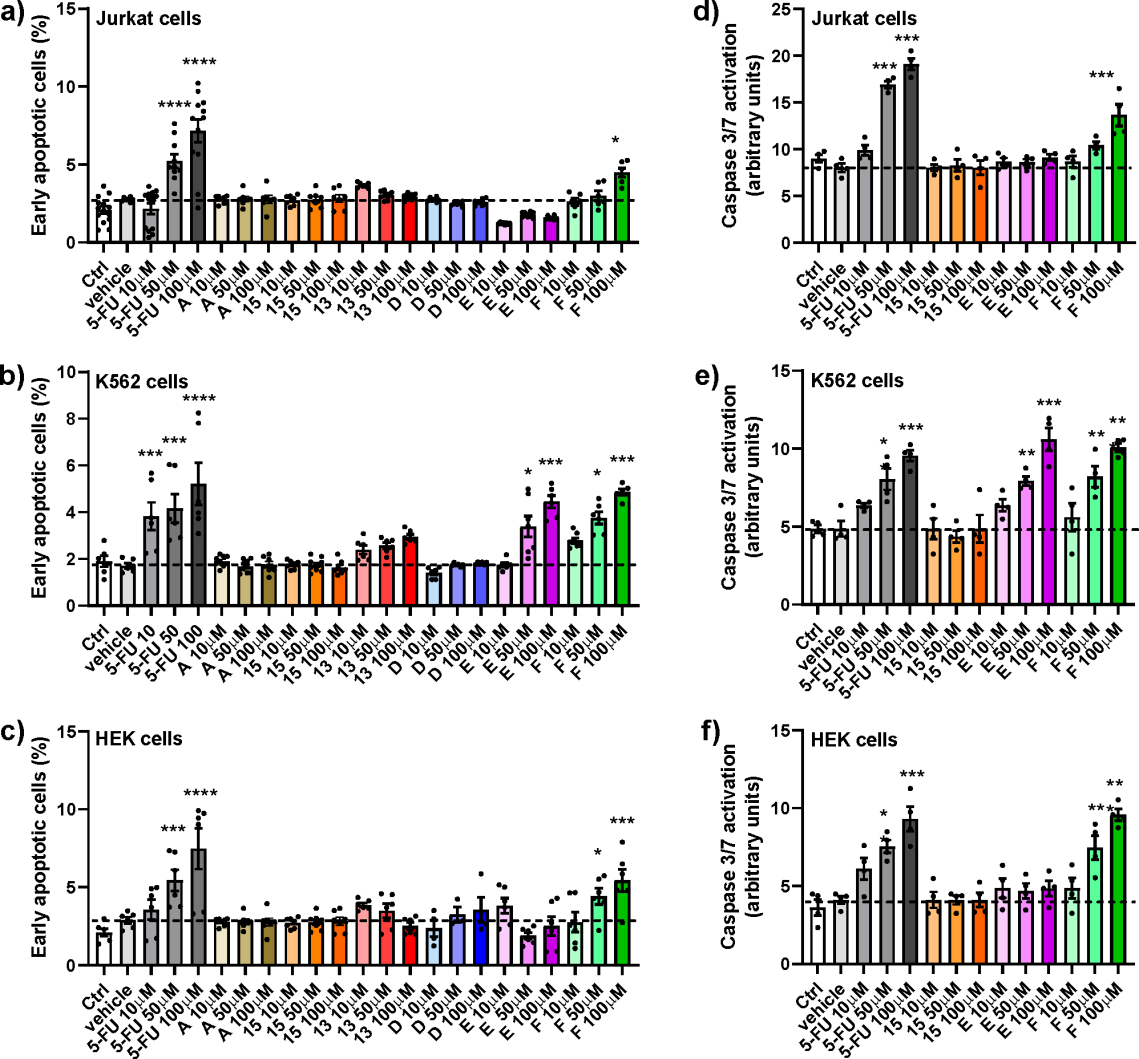
Analysis of apoptosis induced by 5-FU; 5-FU conjugate compounds **D**, **E**, and **F**; and reference compounds **A**, **13**, and **15** (10–50–100 μM), in Jurkat (panels a, d) and K562 (panels b, e) cancer cells and in HEK293 (panels c, f) cell line after 72 h exposure. In panels a–c, apoptosis was determined by flow cytometry to evaluate the ability of the cells to bind annexin V, and the results are presented as the percentage of early apoptotic cells. In panels d–f, caspase 3/7 activation, measured by flow cytometry, is shown. Values are mean ± SD from three independent experiments conducted in triplicate. *p < 0.05; ***p < 0.001; ****p < 0.0001 vs vehicle (Newman-Keuls test after ANOVA).

As shown in Figure 4, 5-FU was able to induce apoptosis in all three cell lines considered; interestingly, the proapoptotic effect of 5-FU was concentration-dependent. The reference compounds **A**, **13**, and **15** were not able to induce apoptosis in all the cell lines employed in this study. In Jurkat cells, which express α_4_β_1_ integrin, only compound **F** induced a significant increase of apoptosis (Figure 4a), only at the highest concentration (100 μM). As regards K562 cells, expressing α_5_β_1_ integrin, compounds **E** and **F** increased significantly apoptotic levels (Figure 4b). The effect of **F** was strongly concentration-dependent and effective as that of 5-FU, at least at the highest concentration (100 μM) (5-FU 100 μM vs **F** 100 μM: not significant). Compound **D** did not induce apoptosis neither in Jurkat nor in K562 cells (Figure 4a,b). In addition, none of the 5-FU conjugates **D** and **E** were able to exert pro-apoptotic effects in HEK293 cells (Figure 4c).

On the contrary, we observed a significant increment of apoptotic levels in HEK293 cells induced by **F** (Figure 4c); this effect could be probably due to the degradation of conjugated compound **F** leading to 5-FU release, as above-mentioned. To further investigate the apoptotic process activated intracellularly by 5-FU conjugated compounds, caspase 3/7 activation was evaluated by flow cytometry as described in the Materials and Methods. Jurkat, K562, and HEK293 cells were exposed to the compounds able to significantly increase the percentage of early apoptotic cells (**E** and **F**, 10–100 μM, for 72 h) in comparison to the unconjugated drug 5-FU. As shown in Figure 4 (panels d–f), 5-FU and compound **F** were able to induce caspase 3/7 activation in all the three cell lines employed in the study, whereas compound **E** activated the proapoptotic pathway through caspase 3/7 only in K562 cells (panel e), thus confirming the results observed in the annexin V assay. These results show that the new 5-FU conjugates exhibit a selective integrin-mediated antitumor activity.

## Discussion and Conclusions

A selective and effective drug delivery is essential for antitumor drugs to selectively target tumor cells and thus for lowering the toxicity against noncancerous cells. Tumor-selective targeting may improve drug delivery through the increment of antineoplastic drug concentration in tumor cells avoiding distribution to other tissues and through the improved distribution of the drug within cancer cells. Moreover, tumor-selective conjugates may surmount anticancer drug resistance possibly through drug conjugate endocytosis.^55−57^

The action of some peptide- or peptidomimetic-conjugates has been already studied as tumor-penetrating molecules.^55,58,59^ To provide a specific receptor targeting for the conjugates, integrins have been identified because they are essential for physiological development and a feature in some diseases, particularly in cancer.^17,50,60^ An active targeting via integrins has been accomplished by including specific integrin ligands in the drug-conjugate, for example RGD peptide, Cilengitide, or others.^61^ Moreover, the use of integrin-targeting small molecules for intracellular delivery of an associated cargo maximizes endocytosis if compared with an integrin-targeting monoclonal antibody.^62^ However, notwithstanding the efforts in the design and realization of the conjugates, less attention was paid to the effective action of the targeting ligand, if it would act as an antagonist or an agonist at the integrin receptor.

Our study was conceived to also investigate this point, and the results show that the new 5-FU conjugates exhibit a selective integrin-mediated antitumor activity. The design of the new derivatives was inspired to β-lactam **A**, taken as a model of a potent and selective agonist of integrins α_4_β_1_. The *o*-tolyl-urea on the nitrogen atom and the carboxylic acid on the C-4 position of the β-lactam ring have to be maintained as substituents in the new compounds because they are crucial for integrin recognition, as previously demonstrated.^32,34^ To covalently anchor fluorescent tags or 5-FU, the same β-lactam skeleton present in **A** was modified with a further C-3 hydroxyethyl side chain, and accordingly, five new β-lactam compounds were obtained.

Preliminary adhesion assays on K562 and Jurkat cells confirmed for all five molecules **B**–**F** a lower potency than the model compound **A**, but unexpectedly, compound **E** showed to be agonist toward integrin α_5_β_1_ and antagonist against integrin α_4_β_1_, whereas compound **F** proved to be an antagonist for both integrins (Table 1). These results show that also the substituent on the C-3 position of the β-lactam ring could play an active role on the receptor response. In fact, ligands with longer C-3 side chains activate the antagonist behavior, and more generally, the presence of a C-3 substituent switches on the activity toward α_5_β_1_ integrin.

It was then demonstrated by flow cytometry that the two fluorescent agonists **B** and **C** undergo internalization in a concentration-dependent manner in Jurkat and K562 cells. This internalization is selectively addressed by integrin binding because upon treatment with the agonist **A** or neutralizing antibodies specific for α_4_ or α_5_ chains, the fluorescent compounds **B** and **C** were no more able to enter within the cells.

The absence of internalization of the two fluorescent β-lactams **B** and **C** in noncancer HEK 293 cells demonstrated the privileged selectivity of the two ligands for cancer cells expressing α_5_β_1_ or α_4_β_1_ integrin. This result is quite important because it could address a positive answer to the meaningful issue of drug discovery with a cell-selective toxicity.

Finally, the three 5-FU-conjugates **D**, **E**, and **F** were evaluated by apoptosis assays in Jurkat, K562, and HEK 293 cells (Figure 4).

Compound **D** (Figure 4, blue bars) was a bad apoptosis inducer for all three cell lines. This behavior could be ascribed to the low stability of compound **D** (Figure 2, panel b), which, following ester hydrolysis, could release 5-FU-acetic acid **13** that was less toxic than 5-FU (Figure 4, red and gray bars, respectively).^63^

Compound **E** (Figure 4, violet bars) showed a selective apoptosis response against K562 cells with a concentration-dependent behavior (Figure 4, panel b). This selectivity could be ascribed to a different integrins expression in the cancer lines (α_5_β_1_ in K562 whereas α_4_β_1_ in Jurkat cells) and, moreover, to the opposing integrin responses of **E** as agonist toward integrins α_5_β_1_ and antagonist for α_4_β_1_ (Table 1). Since **E** was the most stable among the compounds (Figure 2), its behavior as selective apoptosis-inducer gives evidence that conjugate compounds could be more cytotoxic than the parent compound **13**, as already reported for some other conjugates.^63^

Compound **F**, characterized by the glutaryl linker, showed apoptotic effects over all the three cell lines, despite its activity as antagonist toward both α_5_β_1_ and α_4_β_1_ integrins. This widespread cytotoxicity could be due to the low stability of **F** that could release 5-FU in the culture medium. Accordingly, **F** induced a significant increase in apoptosis levels also in noncancer cells HEK293 cells which do not express α_5_β_1_ or α_4_β_1_ integrins. Moreover, the apoptotic effect is particular evident for K562 cells, where at 100 μM the early apoptotic cells are comparable to those of the unconjugated 5-FU (Figure 4, panel b).

On the basis of the results presented here, it seems that integrin ligands behaving as antagonists toward α_5_β_1_ or α_4_β_1_ integrins would not be suitable for a selective delivery of the antineoplastic drug 5-FU against cancer cells. On the contrary, integrin agonists could gain an intracellular delivery of the antineoplastic drug, leading to a selective cancer cell death. Nevertheless, further studies are needed to better clarify which are the most suitable integrin ligands for a selective intracellular delivery of an associated cargo.

## Materials and Methods

Commercial reagents were used as received without additional purification. ^1^H and ^13^C NMR spectra were recorded with an INOVA 400 instrument with a 5 mm probe. All chemical shifts were quoted relative to deuterated solvent signals (δ in ppm and J in Hz). Polarimetric analyses were conducted on Unipol L 1000 “Schmidt–Haensch” polarimeter at 598 nm. FTIR spectra: Bruker Alpha instrument, measured as films between NaCl plates, wave numbers are reported in cm^–1^. The purities of the target compounds **D**, **E**, and **F** were assessed as being >95% using HPLC (Supporting Information). HPLC-MS: Agilent Technologies HP1100 instrument, equipped with a ZORBAX-Eclipse XDB-C8 Agilent Technologies column; mobile phase, H_2_O/CH_3_CN, 0.4 mL/min; gradient from 30 to 80% of CH_3_CN in 8 min, 80% of CH_3_CN until 25 min, coupled with an Agilent Technologies MSD1100 single-quadrupole mass spectrometer, full scan mode from *m*/*z* = 50 to 2600, in positive or negative ion mode. Compound **1**,^32^**13**,^40^ and **14**(42) are known and were synthesized according to reported procedures, spectroscopic data of the compounds were in accordance to those reported in literature.

### General Procedure for Hydrogenolysis (GP1)

A β-lactam benzyl ester (1 equiv) was dissolved in a mixture of THF and CH_3_OH (22 mL/mmol, 1:1 v/v), and Pd/C (10% w/w) was added. The solution was then stirred under a H_2_ atmosphere (1 atm) at room temperature. After a complete consumption of the starting material (TLC monitoring, 2 h) the reaction mixture was filtered through Celite and concentrated in vacuum. The crude was then triturated with a few drops of pentane to afford the desired carboxylic acid.

### General Procedure for N-Boc-Deprotection (GP2)

A *N*-Boc-protected β-lactam (1 equiv) was dissolved in CH_2_Cl_2_ (18.5 mL/mmol) under a nitrogen atmosphere, and trifluoroacetic acid (TFA) (4 equiv) was added dropwise at 0 °C. New TFA aliquots were added each 60 min at 0 °C until a complete conversion (HPLC or TLC monitoring). The solvent was removed under reduced pressure, and the crude was triturated with few drops of pentane to afford the resulting deprotected compound.

#### Benzyl 2-((2*R*, 3*S*)-3-((*R*)-1-((*t*-butyldimethylsilyl)oxy)ethyl)-4-oxo-1-(*o*-tolylcarbamoyl)azetidin-2yl)acetate (**2**)

Compound **1** (80 mg, 0.21 mmol, 1 equiv) was dissolved in anhydrous CH_2_Cl_2_ (2.3 mL) under a nitrogen atmosphere. Anhydrous TEA (148 μL, 1.05 mmol, 5 equiv) was added dropwise, followed by a dropwise addition of *o*-tolylisocyanate (130 μL, 1.05 mmol, 5 equiv). The mixture was stirred at room temperature until complete consumption of the starting β-lactam (16 h, TLC monitoring) and then quenched with a saturated solution of NH_4_Cl. The mixture was extracted with CH_2_Cl_2_ (3 × 10 mL), the organic layers were collected, dried over anhydrous Na_2_SO_4_, concentrated in vacuum, and purified by flash-chromatography (Cyclohexane/EtOAc 90:10), affording the desired compound **2** as a colorless oil (100 mg, 93%).

^1^H NMR: (400 MHz; CDCl_3_) δ = 0.06 (s, 3H), 0.08 (s, 3H), 0.84 (s, 9H), 1.20 (d, *J* = 6.3 Hz, 3H), 2.27 (s, 3H), 2.93 (dd, *J* = 15.5, 7.7 Hz, 1H), 3.10–3.24 (m, 2H), 4.29–4.35 (m, 1H), 4.58–4.61 (m, 1H), 5.16 (s, 2H), 7.05 (t, *J* = 7.3 Hz, 1H), 7.20 (dd, *J* = 14.3, 7.3 Hz, 2H), 7.29–7.38 (m, 5H), 7.91 (d, *J* = 8.1 Hz, 1H), 8.41 (bs, 1H) ppm. ^13^C NMR: (100 MHz; CDCl_3_) δ = −4.9, −3.8, 18.0, 18.1, 22.3, 25.9, 37.3, 49.9, 62.9, 65.0, 67.1, 121.5, 124.7, 127.1, 127.9, 128.7, 128.8, 128.9, 130.7, 135.7, 135.8, 148.2, 168.6, 170.1 ppm. HPLC-MS (ESI^+^): Rt = 18.1 min, *m*/*z* = 511 [M + H]^+^. IR (film): ν = 3341, 3034, 2857, 1766, 1737, 1718, 1548, 1459, 1252 cm^–1^. [α]^D^_20_ = −72 (c = 1, CH_2_Cl_2_).

#### Benzyl 2-((2*R*,3*S*)-3-((*R*)-1-hydroxyethyl)-4-oxo-1-(*o*-tolycarbamoyl)azetidin-2-yl)acetate (**3**)

BF_3_·OEt_2_ (35 μL, 0.28 mmol, 1.15 equiv) was added to a solution of β-lactam **2** (124 mg, 0.24 mmol, 1 equiv) in CH_3_CN (5 mL) at 0 °C under nitrogen atmosphere. After 30 min, the reaction was allowed to warm to room temperature and stirred until a complete consumption of the starting material (2 h, TLC monitoring). The mixture was then quenched with phosphate buffer (0.1M, pH 7.4, 10 mL) and extracted with CH_2_Cl_2_ (3 × 15 mL). The organic layers were collected, dried over Na_2_SO_4_, and concentrated in vacuum. The crude was purified by flash-chromatography (Cyclohexane/EtOAc 70:30) yielding compound **3** as a colorless oil (76 mg, 91%).

^1^H NMR: (400 MHz; CDCl_3_) δ = 1.37 (d, *J* = 6.3 Hz, 3H), 2.28 (s, 3H), 2.77 (dd, *J* = 17.1, 9.8 Hz, 1H), 3.12 (dd, *J* = 7.9, 2.6 Hz, 1H), 3.3 (bs, 1H), 3.52 (dd, *J* = 17.1, 3.6 Hz, 1H), 4.21 (dq, *J* = 12.7, 6.3 Hz, 1H), 4.38 (dt, *J* = 9.8, 3.2 Hz, 1H), 5.16 (d, *J*_*AB*_ = 12.2 Hz, 1H), 5.19 (d, *J*_*AB*_ = 12.2 Hz, 1H), 7.04 (t, *J* = 7.5 Hz, 1H), 7.15–7.25 (m, 2H), 7.28–7.41 (m, 5H), 7.93 (d, *J* = 7.9 Hz, 1H), 8.43 (bs, 1H) ppm. ^13^C NMR: (100 MHz; CDCl_3_) δ = 18.2, 21.7, 37.0, 52.5, 64.0, 66.4, 67.8, 121.4, 125.0, 127.4, 127.9, 129.0, 129.1, 129.2, 131.0, 135.5, 135.7, 148.3, 167.5, 171.7 ppm. HPLC-MS (ESI^+^): Rt = 10.0 min, *m*/*z* = 397 [M + H]^+^ IR (film): ν = 3475, 3340, 2971, 1764, 1735, 1715, 1548, 1459, 1306, 1187 cm^–1^. [α]^D^_20_ = −81 (c = 1, CH_2_Cl_2_)

#### *N*-(9-(2-(((*R*)-1-((2*R*, 3*S*)-2-(2-(Benzyloxy)-2-oxoethyl)-4-oxo-1-(*o*-tolylcarbamoyl)azetidin-3yl)ethoxy)carbonyl)phenyl)-6-(diethylamino)-3*H*-xanthen-3-ylidene)-*N*-ethylethanaminium Chloride (**4**)

To a solution of alcohol **3** (57 mg, 0.14 mmol, 1 equiv) in CH_2_Cl_2_ (2.5 mL) under nitrogen atmosphere, Rhodamine B (67 mg, 0.14 mmol, 1 equiv) and DMAP (3.4 mg, 0.03 mmol, 0.2 equiv) were added. The mixture was then cooled to 0 °C, and DCC (29 mg, 0.14 mmol, 1 equiv) was added; the system was allowed to reach room temperature in 15 min and left under stirring overnight. After 24 h (TLC monitoring), the reaction mixture was filtered washing with CH_2_Cl_2_ (5 mL) and evaporated. The crude was suspended in EtOAc at 0 °C, and the solid residual dicyclohexylurea was eliminated by filtration. The organic layer was concentrated in vacuum and purified by flash-chromatography (CH_2_Cl_2_/EtOAc 50:50 then EtOAc 100% then EtOAc/CH_3_OH 80:20) yielding compound **4** as a purple solid (72 mg, 60%).

^1^H NMR: (400 MHz; CDCl_3_) δ = 1.14 (d, *J* = 6.4 Hz, 3H) 1.29 (t, *J* = 6.9 Hz, 12H), 2.18 (s, 3H), 2.77 (dd, *J* = 16.4, 8.2 Hz, 1H), 3.15 (dd, *J* = 16.3, 4.0 Hz, 1H), 3.31 (dd, *J* = 6.2, 2.7 Hz, 1H), 3.53–3.68 (m, 8H), 4.11–4.17 (m, 1H), 5.07 (s, 2H), 5.22–5.30 (m, 1H), 6.79 (d, *J* = 9.5 Hz, 1H), 6.82–6.90 (m, 3H), 7.01–7.10 (m, 3H), 7.15–7.23 (m, 2H), 7.27–7.36 (m, 6H), 7.70 (t, *J* = 7.7 Hz, 1H), 7.78–7.83 (m, 2H), 8.20 (d, *J* = 7.8 Hz, 1H), 8.27 (bs, 1H), ppm. ^13^C NMR: (100 MHz; CDCl_3_) δ = 12.8, 17.7, 17.8, 36.5, 46.3, 51.4, 60.0, 66.9, 68.5, 96.3, 96.5, 113.5, 113.5, 114.3, 114.6, 121.1, 124.8, 126.9, 127.6, 128.4, 128.6, 128.7, 129.9, 130.4, 130.5, 130.6, 131.2, 131.3, 133.5, 133.6, 135.1, 135.4, 147.5, 155.5, 155.6, 155.7, 157.7, 157.9, 158.3, 164.3, 166.0, 169.5 ppm. HPLC-MS (ESI^+^): Rt = 9.10 min, *m*/*z* = 821 [M-Cl-]^+^. IR (film): ν = 3338, 3062, 2976, 2929, 2855, 1766, 1720, 1647, 1592, 1548, 1413, 1338, 1250, 1181, 1133, 1076 cm^–1^.

#### *N*-(9-(2-(((*R*)-1-((2*R*,3*S*)-2-(Carboxymethyl)-4-oxo-1-(*o*-tolylcarbamoyl)azetidin-3yl)ethoxy)carbonyl)phenyl)-6-(diethyl amino)-3*H*-xanthen-3-ylidene)-*N*-ethylethanaminium Chloride (**B**)

Following GP1 compound **4** (38 mg, 0.04 mmol) yielded compound **B** as a purple solid (31 mg, 92%).

^1^H NMR: (400 MHz; CD_3_OD) δ = 0.99 (d, *J* = 6.4 Hz, 3H), 1.28–1.32 (m, 12H), 2.17 (s, 3H), 2.45 (dd, *J* = 14.9, 8.7 Hz, 1H), 2.94 (dd, *J* = 14.9, 3.6 Hz, 1H), 3.30–3.32 (m, 1H), 3.60–3.70 (m, 8H), 3.90–3.93 (m, 1H), 5.28–5.34 (m, 1H), 6.98–7.09 (m, 6H), 7.14–7.20 (m, 3H), 7.43 (d, *J* = 7.4 Hz, 1H), 7.68–7.78 (m, 2H), 7.83 (t, *J* = 7.5 Hz, 1H), 8.25 (d, *J* = 7.7 Hz, 1H) ppm. ^13^C NMR: (100 MHz; CD_3_OD) δ = 12.8, 17.4, 17.8, 34.6, 46.7, 52.5, 61.1, 69.2, 97.5, 97.6, 114.4, 115.4, 116.0, 122.9, 126.0, 127.5, 129.9, 131.3, 131.9, 132.3, 133.4, 134.1, 136.2, 149.1, 152.8, 156.8, 157.0, 158.7, 159.1, 165.7, 167.9, 168.7, 168.9 ppm. HPLC-MS (ESI^+^): Rt = 8.03 min, *m*/*z* = 731 [M-Cl-]^+^. IR (film): ν = 3341, 2975, 2931, 1765, 1716, 1648, 1590, 1339, 1250, 1133, 1011 cm^–1^.

#### (*R*)-1-((2*R*, 3*S*)-2-(2-(Benzyloxy)-2-oxoethyl)-4-oxo-1-(*o*-tolylcarbamoyl)azetidin-3-yl)ethyl 3-((tert-butoxycarbonyl)amino)propanoate (**5**)

Following the procedure reported for **4**, alcohol **3** (98 mg, 0.25 mmol, 1 equiv) was treated with Boc-β-alanine (76 mg, 0.4 mmol, 1.6 equiv), DMAP (6 mg, 0.05 mmol, 0.2 equiv), and DCC (83 mg, 0.4 mmol, 1.6 equiv). Purification by flash-chromatography (Cyclohexane/EtOAc 80:20) yielded compound **5** as a colorless oil (127 mg, 90%).

^1^H NMR: (400 MHz; CDCl_3_) δ = 1.38 (d, *J* = 4.3 Hz, 3H) 1.43 (s, 9H), 2.28 (s, 3H), 2.52 (t, *J* = 4.5 Hz, 2H), 2.86 (dd, *J* = 15.3, 7.4 Hz, 1H), 3.28–3.45 (m, 4H), 4.39–4.46 (m, 1H), 5.08 (bs, 1H), 5.15 (s, 2H), 5.29–5.39 (m, 1H), 7.05 (t, *J* = 7.4 Hz, 1H), 7.15–7.25 (m, 2H), 7.28–7.38 (m, 5H), 7.92 (d, *J* = 8.0 Hz, 1H), 8.38 (bs, 1H) ppm. ^13^C NMR: (100 MHz; CDCl_3_) δ = 17.8 18.4, 27.1, 28.6, 36.3, 36.7, 51.8, 60.3, 67.1, 67.6, 79.6, 121.2, 124.8, 127.0, 127.6, 128.6, 128.7, 128.8, 130.7, 135.3, 135.5, 147.7, 156.0, 166.4, 169.7, 171.7, ppm. HPLC-MS (ESI^+^): Rt = 12.0 min *m*/*z* = 468 [M-Boc+H]^+^. IR (film): ν = 3344, 2977, 2932, 1766, 1736, 1718, 1594, 1546, 1459, 1252, 1170 cm^–1^. [α]^D^_20_ = −25 (c = 0.6, CH_2_Cl_2_)

#### 2-((2*R*, 3*S*)-3-((*R*)-1-((3-((*tert*-Butoxycarbonyl)amino) propanoyl)oxy)ethyl)-4-oxo-1-(*o*-tolylcarbamoyl)azetidin-2-yl)acetic Acid (**6**)

Following GP1, compound **5** (107 mg, 0.19 mmol) yielded compound **6** as a colorless oil (86 mg, 95%).

^1^H NMR: (400 MHz; CD_3_OD) δ = 1.35 (d, *J* = 6.4 Hz, 3H) 1.37 (s, 9H), 2.22 (s, 3H), 2.48 (t, *J* = 6.7 Hz, 2H), 2.79 (dd, J = 15.9, 8.4 Hz, 1H), 3.17–3.21 (m, 1H), 3.23–3.29 (m, 2H), 3.48 (dd, *J* = 6.3, 2.1 Hz, 1H), 4.35–4.42 (m, 1H), 5.27–5.36 (m, 1H), 7.01 (t, *J* = 7.4 Hz, 1H), 7.12–7.20 (m, 2H), 7.74 (d, *J* = 7.9 Hz, 1H) ppm. ^13^C NMR: (100 MHz; CD_3_OD) δ = 17.8, 18.5, 28.7, 35.6, 37.3, 53.1, 61.3, 68.9, 80.2, 123.0, 126.0, 127.6, 130.0, 131.5, 136.4, 149.6, 158.2, 168.2, 172.5 ppm. HPLC-MS (ESI^+^): Rt = 9.58 min *m*/*z* = 378 [M-Boc+H]^+^. IR (film): ν = 3343, 2979, 2936, 1766, 1738, 1714, 1615, 1593, 1252, 1171 cm^–1^. [α]^D^_20_ = −45 (c = 1, CH_2_Cl_2_)

#### 2,2,2-Trifluoroacetate, 3-((*R*)-1-((2*R*,3*S*)-2-(carboxymethyl)-4-oxo-1-(*o*-tolylcarbamoyl)azetidin-3yl)ethoxy)-3-oxopropan-1-aminium Salt (**7**)

Following GP2, compound **6** (51 mg, 0.11 mmol, 1 equiv) yielded compound **7** as a colorless oil (54 mg, 99%).

^1^H NMR: (400 MHz; CD_3_CN) δ = 1.40 (d, *J* = 6.4 Hz, 3H), 2.25 (s, 3H), 2.67–2.78 (m, 3H), 3.12–3.42 (m, 5H), 4.36 (dt, *J* = 9.7, 3.1 Hz, 1H), 5.39–5.51 (m, 1H), 7.0 (bs, 2H), 7.07 (t, *J* = 7.5 Hz, 1H), 7.17–7.25 (m, 2H), 7.83 (d, *J* = 8.1 Hz, 1H), 8.39 (bs, 1H) ppm. ^13^C NMR: (100 MHz; CD_3_CN) δ = 17.9, 18.8, 32.1, 36.9, 37.1, 53.7, 61.6, 69.5, 122.5, 125.7, 127.7, 129.5, 131.6, 136.7, 149.3, 167.4, 171.6, 173.4 ppm. HPLC-MS (ESI^+^): Rt = 1.7 min m/z = 378 [M-TFA+H]^+^. IR (film): ν = 3340, 3067, 2984, 2940, 2727, 2575, 1771, 1733, 1726, 1718, 1678, 1594, 1337, 1308, 1182 cm^–1^. [α]^D^_20_ = −34 (c = 1, CH_2_Cl_2_).

#### 2-((2*R*,3*S*)-3-((*R*)-1-((3-(3-(3′,6′-Dihydroxy-3-oxo-3*H*-spiro[isobenzofuran-1,9′-xanthen]-5yl)thioureido)propanoyl)oxy)ethyl)-4-oxo-1-(*o*-tolylcarbamoyl)azetidin-2-yl)acetic Acid (**C**)

Compound **7** (46 mg, 0.09 mmol, 1 equiv) was dissolved in anhydrous CH_2_Cl_2_ (1 mL) under a nitrogen atmosphere. Anhydrous TEA (50 μL, 0.36 mmol, 4 equiv) was added dropwise, followed by a dropwise addition of FITC (32 mg, 0.08 mmol, 0.9 equiv). After complete consumption of the starting β-lactam (4 h), the solvent was evaporated in vacuum, and the crude was redissolved in CH_3_OH (1 mL). Then water and HCl (1M) were added until pH = 3 to litmus. The aqueous solution was extracted with EtOAc (3 × 10 mL). The organic layers were collected, dried over Na_2_SO_4_, and concentrated in vacuum. Purification by flash chromatography (EtOAc/CH_3_OH 80:20 then 70:30) yielded compound **C** as an orange solid (38 mg, 55%).

^1^H NMR: (400 MHz; CD_3_OD) δ = 1.39 (d, *J* = 6.4 Hz, 3H), 2.21 (s, 3H), 2.63 (dd, *J* = 15.3, 9.1 Hz, 1H), 2.70 (t, *J* = 6.2 Hz, 2H), 3.14 (dd, *J* = 15.3, 3.6 Hz, 1H), 3.47 (dd, *J* = 7.2, 2.4 Hz, 1H), 3.80–3.90 (m, 2H), 4.43 (dd, *J* = 5.8, 3.1 Hz, 1H), 5.33–5.42 (m, 1H), 6.51 (d, *J* = 8.7, 2H), 6.60–6.70 (m, 4H), 6.99 (t, *J* = 7.4 Hz, 1H), 7.05–7.18 (m, 3H), 7.70–7.76 (m, 2H), 8.18 (bs, 1H) ppm. ^13^C NMR: (100 MHz; CD_3_OD) δ = 18.1, 18.8, 34.9, 39.3, 41.3, 54.2, 61.7, 69.4, 103.8, 112.0, 114.4, 116.9, 120.7, 123.4, 126.1, 126.3, 127.8, 129.6, 130.4, 130.7, 131.7, 136.36 142.6, 148.6, 150.0, 154.7, 162.5, 168.6, 171.6, 173.1, 176.1, 183.0 ppm. HPLC-MS (ESI^+^): Rt = 8.4 min *m*/*z* = 767 [M + H]^+^. IR (film): ν = 3374, 2972, 2936, 1764, 1710, 1688, 1594, 1545, 1460, 1310, 1205 cm^–1^

#### 2,2,2-Trifluoroacetate, 3-((*R*)-1-((2*R*,3*S*)-2-(2-(benzyloxy)-2-oxoethyl)-4-oxo-1-(*o-*tolyl carbamoyl)azetidin-3-yl)ethoxy)-3-oxopropan-1-aminium Salt (**8**)

Following GP2, compound **5** (59 mg, 0.10 mmol, 1 equiv) yielded compound **8** as a colorless oil (56 mg, 96%).

^1^H NMR: (400 MHz, CD_3_CN) δ = 1.36 (d, *J* = 6.4 Hz, 3H), 2.25 (s, 3H), 2.71 (t, *J* = 6.3 Hz, 2H), 2.82–3.08 (m, 4H), 3.17–3.26 (m, 3H), 3.46 (dd, *J* = 7.3, 2.7 Hz, 1H), 4.39 (ddd, *J* = 8.1, 4.0, 2.7 Hz, 1H), 5.12 (d, *J* = 12.4 Hz, 1H), 5.16 (d, *J* = 12.4 Hz, 1H), 5.34–5.42 (m, 1H), 7.05–7.10 (m, 2H), 7.17–7.26 (m, 2H), 7.32–7.40 (m, 4H), 7.82 (d, *J* = 8.1 Hz, 1H), 8.36 (bs, 1H) ppm. ^13^C NMR: (100 MHz, CDCl_3_) δ = 17.5, 18.1, 30.7, 36.3, 36.6, 52.1, 60.7, 67.3, 68.9, 122.0, 125.6, 127.0, 128.2, 128.7, 128.8, 130.8, 134.5, 135.2, 148.3, 165.9, 170.5, 171.3 ppm. ESI-MS (ESI^+^): Rt = 1.7 min, *m*/*z* = 468 [M-TFA]^+^. IR (film): ν = 3342, 3064, 2930, 1768, 1734, 1680, 1594, 1460, 1203 cm^–1^. [α]^D^_20_ = −16 (c = 0.9, CH_2_Cl_2_)

#### (*R*)-1-((2*R*,3*S*)-2-(2-(Benzyloxy)-2-oxoethyl)-4-oxo-1-(*o*-tolyl carbamoyl) azetidin-3-yl)ethyl 3-(2-(5-fluoro2,6-dioxo-2,3-dihydropyrimidin-1(6*H*)-yl)acetamido)propanoate (**9**)

In a round-bottom flask, compound **8** (51 mg, 0.09 mmol, 1.2 equiv) was dissolved in CH_2_Cl_2_ (0.5 mL) under a nitrogen atmosphere, and anhydrous TEA (15 μL, 0.108 mmol, 1.4 equiv) was added dropwise. The reaction was left for 20 min in order to desalt compound **8**. At the same time, in a second round-bottom flask, compound **13** (14 mg, 0.075 mmol, 1 equiv) was dissolved in DMF (0.1 mL), and the solution of the first round-bottom flask was dropped. Then HOBt (10 mg, 0.075 mmol, 1 equiv) and EDC (14 mg, 0.075 mmol, 1 equiv) were added at 0 °C. After 1 h, the solution was warmed to rt and left under stirring after complete consumption of the starting material (18 h, TLC monitoring). The mixture was quenched with H_2_O and extracted with CH_2_Cl_2_ (3 × 10 mL). The organic layers were collected, dried over Na_2_SO_4_, and concentrated in vacuum. Purification by flash chromatography (Cyclohexane/EtOAc 30:70 than 20:80) yielded compound **9** as a waxy white solid (19 mg, 40%).

^1^H NMR: (400 MHz, CDCl_3_) δ = 1.39 (d, *J* = 6.3 Hz, 3H), 2.28 (s, 3H), 2.45–2.62 (m, 2H), 2.79 (dd, *J* = 16.2, 9.1 Hz, 1H), 3.32 (dd, *J* = 7.8, 2.1 Hz, 1H), 3.38 (dd, *J* = 16.3, 3.6 Hz, 1H), 3.47–3.57 (m, 2H), 4.16 (d, *J* = 15.6 Hz, 1H), 4.26 (d, *J* = 15.6 Hz, 1H), 4.38–4.44 (m, 1H), 5.14 (s, 2H), 5.32–5.42 (m, 1H), 6.90–7.09 (m, 2H), 7.18–7.22 (m, 2H), 7.30–7.37 (m, 5H), 7.91 (d, *J* = 8.1 Hz, 1H), 8.35 (bs, 1H), 9.48 (bs, 1H) ppm. ^13^C NMR: (100 MHz, CDCl_3_) δ = 18.1, 18.7, 34.1, 35.6, 37.2, 51.1, 52.1, 60.7, 67.5, 68.2, 121.6, 122.5, 125.3, 125.9, 127.3, 128.1, 128.7, 129.0 (d, J = 33 Hz), 130.0, 135.5, 139.8 (d, J = 219 Hz) 148.2, 150.2, 157.5 (d, J = 26.5 Hz), 166.7, 170.4, 171.9 ppm. HPLC-MS (ESI^+^): Rt= 8.2 min, *m*/*z* = 638 [M + H]^+^. IR (film): ν = 3367, 3060, 2990, 1765, 1693, 1662, 1615, 1543, 1241, 1169, 1133 cm^–1^. [α]^D^_20_ = −43 (c = 1, CH_3_OH).

#### 2-((2*R*,3*S*)-3-((*R*)-1-((3-(2-(5-Fluoro-2,6-dioxo-2,3-dihydro pyrimidin-1(6*H*)yl)acetamido)propanoyl)oxy)ethyl)-4-oxo-1-(*o*-tolylcarbamoyl)azetidin-2-yl)acetic Acid (**E**)

Following GP1, compound **9** (13 mg, 0.02 mmol) yielded compound **E** as a waxy white solid (11 mg, 99%).

^1^H NMR: (400 MHz, CD_3_OD) δ = 1.41 (d, *J* = 7.4 Hz, 3H) 2.28 (s, 3H), 2.59 (dd, *J* = 10.9, 6.0 Hz, 1H), 2.68 (dd, *J* = 15.3, 8.9 Hz, 1H), 3.17–3.22 (m, 1H), 3.38–3.54 (m, 3H), 4.37 (s, 2H), 4.40–4.43 (m, 1H), 5.31–5.43 (m, 1H), 7.03–7.08 (m, 1H), 7.19 (m, 2H), 7.74 (d, *J* = 6.2 Hz, 1H), 7.76 (d, *J* = 8.2 Hz, 1H) ppm. ^13^C NMR: (100 MHz, CD_3_OD) δ = 18.1, 18.8, 35.2, 36.8, 38.0, 51.5, 53.7, 61.8, 69.5, 123.6, 126.5, 128.0, 130.7, 131.9, 132.1 (d, *J* = 33.9 Hz), 136.8, 141.9 (d, *J* = 232.0 Hz), 150.1, 151.9, 160.3 (d, J = 25.9 Hz), 168.6, 169.6, 172.7 ppm. HPLC-MS (ESI^+^): Rt= 5.01 min; *m*/*z* = 548 [M + H]^+^. IR (film): ν = 3352, 2930, 1763, 1697, 1664, 1592, 1542, 1244, 1176 cm^–1^. [α]^D^_20_ = −45 (c = 1.1, CH_3_OH).

#### Benzyl 2-((2*R*,3S)-3-((*R*)-1-(2-(5-Fluoro-2,4-dioxo-3,4-dihydropyrimidin-1(2*H*)-yl)acetoxy)ethyl)-4-oxo-1-(*o*-tolylcarbamoyl)azetidin-2-yl)acetate (**10**)

Compound **3** (65 mg, 0.17 mmol, 1 equiv) was dissolved in MeCN (1.6 mL) under a nitrogen atmosphere. Compound **13** (31 mg, 0.17 mmol, 1 equiv) was dissolved in DMF (1.7 mL) and added dropwise, followed by the addition of DCC (38 mg, 0.18 mmol, 1.1 equiv) and DMAP (4 mg, 0.033 mmol, 0.2 equiv). The reaction was left under stirring after complete consumption of the starting material (48 h, TLC monitoring). The reaction mixture was filtered, washed with CH_2_Cl_2_ (5 mL), and evaporated. The crude was suspended in EtOAc at 0 °C, and the solid residual dicyclohexylurea was eliminated by filtration. The organic layer was concentrated in vacuum and purified by flash chromatography (CH_2_Cl_2_/EtOAc 70:30), yielding compound **10** as a waxy solid (21 mg, 23%).

^1^H NMR: (400 MHz, CDCl_3_) δ = 1.46 (d, *J* = 6.4 Hz, 3H), 2.28 (s, 3H), 2.74 (dd, *J* = 17.2, 9.9 Hz, 1H), 3.24 (dd, *J* = 8.9, 2.4 Hz, 1H), 3.55 (dd, *J* = 17.1, 3.3 Hz, 1H), 4.17 (d, *J* = 17.6 Hz, 1H), 4.43 (dt, *J* = 9.9, 2.8 Hz, 1H), 4.65 (d, *J* = 17.5 Hz, 1H), 5.14 (d, *J* = 12.5 Hz, 1H), 5.17 (d, *J* = 12.5 Hz, 1H), 5.47 (dq, *J* = 12.8, 6.4 Hz, 1H), 7.05 (t, *J* = 7.5 Hz, 1H), 7.15–7.20 (m, 2H), 7.32–7.38 (m, 6H), 7.91 (d, *J* = 8.1 Hz, 1H), 8.36 (s, 1H), 9.29 (s, 1H) ppm. ^13^C NMR: (100 MHz, CDCl_3_) δ = 17.8, 18.6, 36.7, 48.7, 52.1, 61.2, 67.2, 69.4, 121.2, 124.9, 127.0, 127.7, 128.1, 128.8, 129.0 (d, J = 19 Hz), 130.6, 135.2, 135.3, 140.6 (d, *J* = 237 Hz), 147.6, 149.6, 157.1 (d, *J* = 26 Hz), 165.5, 166.9, 170.4 ppm. HPLC-MS (ESI^+^): Rt = 9.81 min; *m*/*z* = 567 [M + H]^+^. IR (film): ν = 3338, 3204, 3067, 2933, 1764, 1703, 1669, 1616, 1593, 1548, 1460, 1381, 1249, 1210 cm^–1^. [α]^D^_20_ = −6.5 (c = 1.3, CH_2_Cl_2_)

#### 2-((2*R*,3*S*)-3-((*R*)-1-(2-(5-Fluoro-2,4-dioxo-3,4-dihydropyrimidin-1(2*H*)-yl)acetoxy)ethyl)-4-oxo-1-(*o*-tolylcarbamoyl)azetidin-2-yl)acetic Acid (**D**)

Following GP1, compound **10** (21 mg, 0.04 mmol) yielded compound **D** as a waxy white solid (15 mg, 86%).

^1^H NMR: (400 MHz, CD_3_OD) δ = 1.45 (d, *J* = 6.4 Hz, 3H) 2.28 (s, 3H), 2.79 (dd, *J* = 16.0, 9.0 Hz, 1H), 3.23 (dd, *J* = 16.0, 3.1 Hz, 1H), 3.52 (dd, *J* = 7.2, 2.4 Hz, 1H), 4.40 (dd, *J* = 5.9, 2.8 Hz, 1H), 4.48 (d, *J* = 17.5 Hz, 1H), 4.58 (d, *J* = 17.5 Hz, 1H), 5.38–5.46 (m, 1H), 7.06 (t, *J* = 7.4 Hz, 1H), 7.14–7.24 (m, 2H), 7.80 (d, *J* = 8.0 Hz, 1H), 7.85 (d, *J* = 6.1 Hz, 1H) ppm. ^13^C NMR: (100 MHz, CD_3_OD) δ = 17.8, 18.5, 37.9, 50.2, 53.7, 61.5, 70.5, 123.0, 126.0, 127.6, 130.0, 131.1, 131.4 (d, *J* = 3.6 Hz), 136.5, 141.6 (d, *J* = 232.8 Hz), 149.7, 151.3, 159.8 (d, *J* = 53 Hz), 167.8, 168.6 ppm. HPLC-MS (ESI^+^): Rt = 6.69 min; *m*/*z* = 477 [M + H]^+^. IR (film): ν = 3424, 1762, 1641, 1551, 1460, 1250, 1214 cm^–1^. [α]^D^_20_ = −14 (c = 1, CH_2_Cl_2_).

#### 5-((*R*)-1-((2*R*,3*S*)-2-(2-(Benzyloxy)-2-oxoethyl)-4-oxo-1-(*o*-tolylcarbamoyl)azetidin-3-yl)ethoxy)-5-oxopentanoic Acid (**11**)

Compound **3** (49 mg, 0.12 mmol, 1.0 equiv) was dissolved in CH_2_Cl_2_ (3.5 mL) under a nitrogen atmosphere. Glutaric anhydride (28 mg, 0.24 mmol, 2.0 equiv), DMAP (3 mg, 0.024 mmol, 0.2 equiv), and TEA (34 μL, 0.24 mmol, 2 equiv) were then added. The solution was left under stirring after complete consumption of the starting material (18 h, TLC monitoring). The mixture was quenched with H_2_O (1 mL) and HCl 2N (2 mL) and extracted with CH_2_Cl_2_ (3 × 10 mL). The organic layers were collected, dried over anhydrous Na_2_SO_4_, and concentrated in vacuum to afford compound **11** as a colorless oil (60 mg, 95%).

^1^H NMR: (400 MHz, CDCl_3_) δ = 1.36 (d, *J* = 6.4 Hz, 3H) 1.87–1.98 (m, 2H), 2.27 (s, 3H), 2.45–2.32 (m, 4H), 2.88 (dd, *J* = 16.2, 8.2 Hz, 1H), 3.29 (dd, *J* = 16.1, 4.0 Hz, 1H), 3.35 (dd, *J* = 6.3, 2.7 Hz, 1H), 4.36–4.50 (m, 1H), 5.13 (s, 2H), 5.33 (p, *J* = 6.4 Hz, 1H), 7.01–7.10 (m, 1H), 7.11–7.22 (m, 2H), 7.29–7.38 (m, 5H), 7.92 (d, *J* = 8.1 Hz, 1H), 8.38 (bs, 1H) ppm. ^13^C NMR: (100 MHz, CDCl_3_) δ = 17.6, 18.1, 19.7, 32.6, 33.1, 36.6, 51.4, 60.1, 66.9, 67.1, 120.9, 124.5, 126.8, 127.8, 128.0, 128.2, 128.6, 130.2, 135.2, 135.4, 147.6, 166.3, 169.5, 171.8, 177.5 ppm. HPLC-MS (ESI^+^): Rt = 10.01 min, *m*/*z* = 511 [M + H]^+^. IR (film): ν = 3344, 3055, 2984, 2928, 1769, 1736, 1719, 1710, 1614, 1593, 1545 cm^–1^. [α]^D^_20_ = −36 (c= 1, CH_2_Cl_2_).

#### (*R*)-1-((2*R*,3*S*)-2-(2-(Benzyloxy)-2-oxoethyl)-4-oxo-1-(*o*-tolylcarbamoyl)azetidin-3-yl)ethyl((5-fluoro-2,4-dioxo-3,4-dihydropyrimidin-1(2*H*)-yl)methyl)glutarate (**12**)

In a first round-bottom flask, 5-FU (55 mg, 0.42 mmol, 1.8 equiv) was dissolved in H_2_O (3.5 mL), and paraformaldehyde (19 mg, 0.64 mmol, 2.7 equiv) was added. The reaction was left 6 h at 60 °C and then H_2_O was concentrated in vacuum. The residual was dissolved in MeCN (5.8 mL) and transferred in a second round-bottom flask under nitrogen atmosphere, then compound **11** (115 mg, 0.23 mmol, 1 equiv) was added followed by EDC (61 mg, 0.32 mmol, 1.4 equiv) and DMAP (39 mg, 0.32 mmol, 1.4 equiv). The mixture was left under stirring after complete consumption of the starting material (18 h, TLC monitoring). The mixture was quenched with H_2_O and extracted with CH_2_Cl_2_ (3 × 10 mL). The organic layers were collected, dried over anhydrous Na_2_SO_4_, and concentrated in vacuum. Purification by flash chromatography (Cyclohexane/EtOAc 45:55) yielded compound **12** as a waxy white solid (27 mg, 18%).

^1^H NMR: (400 MHz, CDCl_3_) δ = 1.35 (d, *J* = 6.2 Hz, 3H), 1.86–1.98 (m, 2H), 2.27 (s, 3H), 2.31–2.48 (m, 4H), 2.88 (dd, J = 16.0, 8.0 Hz, 1H), 3.25–3.35 (m, 2H), 4.42–4.47 (m, 1H), 5.16 (s, 2H), 5.25–5.39 (m, 1H), 5.54 (s, 2H), 7.05 (t, *J* = 7.1 Hz, 1H), 7.16–7.24 (m, 2H), 7.30–7.36 (m, 5H), 7.53 (d, *J* = 4.8 Hz, 1H), 7.92 (d, *J* = 7.9 Hz, 1H), 8.38 (bs, 1H), 8.83 (bs, 1H) ppm. ^13^C NMR: (100 MHz, CDCl_3_) δ = 17.8. 18.3, 19.8, 32.9, 33.1, 36.8, 51.4, 60.2, 67.1, 67.3, 70.0, 121.1, 124.8, 127.0, 127.6, 128.5, 128.6, 128.8, 130.3 (d, *J* = 30 Hz), 135.2, 135.8, 140.8 (d, *J* = 289 Hz), 147.8, 149.2, 156.5 (d, *J* = 28 Hz), 166.5, 169.7, 171.8, 173.0 ppm. HPLC-MS (ESI^+^): Rt = 9.91 min, *m*/*z* = 653 [M + H]^+^. IR (film): ν = 3210, 3088, 2986, 1763, 1719, 1709, 1677, 1252, 1126 cm^–1^. [α]^D^_20_ = −24 (c= 0.9, CH_2_Cl_2_).

#### 2-((2*R*,3*S*)-3-((*R*)-1-((5-((5-Fluoro-2,4-dioxo-3,4-dihydro pyrimidin-1(2*H*)-yl)methoxy)-5-oxopentanoyl)oxy)ethyl)-4-oxo-1-(*o*-tolylcarbamoyl)azetidin-2-yl)acetic Acid (**F**)

Following GP1, compound **12** (27 mg, 0.04 mmol) yielded compound **F** as a waxy white solid (21 mg, 93%).

^1^H NMR: (400 MHz, CDCl_3_) δ = 1.42 (d, *J* = 6.0 Hz, 3H), 1.89–2.0 (m, 2H), 2.29 (s, 3H), 2.31–2.56 (m, 4H), 2.74 (m, 1H), 3.35 (d, *J* = 6.5 Hz, 1H), 3.41 (d, *J* = 14.9 Hz, 1H), 4.40–4.43 (m, 1H), 5.29–5.37 (m, 1H), 5.56 (d, *J* = 10.6 Hz, 1H), 5.64 (d, *J* = 10.4 Hz, 1H), 7.05 (dd, *J* = 20.6, 13.2 Hz, 1H), 7.13–7.26 (m, 2H), 7.59 (d, *J* = 4.9 Hz, 1H), 7.91 (d, *J* = 8.1 Hz, 1H), 8.43 (bs, 1H), 10.04 (bs, 1H) ppm. ^13^C NMR: (100 MHz, CDCl_3_) δ = 17.6, 18.3, 19.6, 30.3, 32.7, 36.9, 51.9, 60.4, 67.6, 70.2, 121.1, 124.8, 125.5, 126.8, 127.7, 128.6 (d, *J* = 34 Hz), 130.5, 135.0, 140.4 (d, *J* = 238 Hz), 147.8, 149.7, 157,2 (d, *J* = 26 Hz), 166.2, 171.8, 172.9, 173.2 ppm. HPLC-MS (ESI^+^): Rt = 6.36 min, *m*/*z* = 563 [M + H]^+^. IR (film): ν = 3192, 3059, 2963, 1735, 1718, 1701, 1686, 1250, 1124, 1082 cm^–1^. [α]^D^_20_ = −32 (c = 0.7, CH_2_Cl_2_).

#### 2-((2*R*,3*S*)-3-((*R*)-1-((3-((*tert*-Butoxycarbonyl)amino)propanoyl)oxy)ethyl)-4-oxo-1-(*o*-tolylcarbamoyl)azetidin-2-yl)acetic Acid (**15**)

Following GP1, compound **5** (31 mg, 0.06 mmol) yielded compound **15** as a waxy oil (29 mg, > 99%).

^1^H NMR: (400 MHz, CDCl_3_) δ = 1.37 (d, J = 6.7 Hz, 12H), 2.24 (s, 3H), 2.48 (t, J = 6.7 Hz, 2H), 2.78 (dd, J = 16.0, 8.4 Hz, 1H), 3.29–3.04 (m, 5H), 3.49 (dd, J = 6.2, 2.6 Hz, 1H), 4.39 (dt, J = 7.1, 3.1 Hz, 1H), 5.32 (p, J = 6.3 Hz, 1H), 7.07–6.97 (m, 1H), 7.21–7.09 (m, 2H), 7.73 (d, J = 8.0 Hz, 1H). HPLC-MS (ESI^+^): Rt = 8,34 min, *m*/*z* = 378 [M-Boc+H]^+^.

### Stability Tests

Compounds **D**, **E**, and **F** were dissolved in PBS 0.1 M pH = 7.4 (1 mg/mL) and incubated at 30 °C in a thermostat. Aliquots (0.5 mL) were taken at different time points (from 0 to 72 h) and analyzed by HPLC-UV using Zorbax-Eclipse XDB column – C18, 4.6 × 150 mm, 5 μm for **D** and **E**, and Gemini column – C18, 100 × 2 mm, 3 μm for **F**. Peaks relative to the intact compounds were integrated, and their concentration was determined at the established times to obtain a stability profile of the compounds in PBS.

Compounds **D**, **E**, and **F** were dissolved in fetal bovine serum (1 mg/mL) and incubated at 30 °C in a thermostat. Aliquots of 0.15 mL were taken at different time points (from 0 to 72 h) and diluted with 0.6 mL of MeOH. After centrifugation for 3 min at 50 rpm, 0.4 mL of the supernatant were taken and for compound **D** and **E** directly analyzed in HPLC-UV using Gemini column – C18 100 × 2 mm 3 μm; for compound **F** instead, the supernatant was concentrated, and the resulting solid material was redissolved in 0.2 mL of Milli-Q water and 0.2 mL of MeCN and analyzed as described above. Peaks relative to the intact compounds were integrated and their concentration was determined at the established times to obtain a stability profile of the compounds in FBS.

Compounds **E** and **F** were dissolved in PBS 0.1 M pH = 6 (1 mg/mL) and incubated at 30 °C in a thermostat. Aliquots (0.5 mL) were taken at different time points (from 0 to 72 h) and analyzed by HPLC-UV InfinityLab with a column Poroshell 120 EC-C18 3.0 × 150 mm 2.7 μm, flow 0.3 mL/min, 40 °C. Peaks relative to the intact compounds were integrated, and their concentration was determined at the established times to obtain the stability profile of the compounds (see Supporting Information).

### Cell Culture

Jurkat E6.1 human T (an immortalized cell line from human blood leukemic T-cell lymphoblasts), HT-29 (human colorectal adenocarcinoma cell line) and K562 (human erythroleukemic cell line) cells were grown in RPMI-1640 (Life Technologies, Carlsbad, CA, U.S.A.) supplemented with l-glutamine and 10% FBS (fetal bovine serum; Life technologies). K562 cells were treated with 25 ng/mL PMA (Phorbol 12-myristate 13-acetate, Sigma-Aldrich SRL, Milan, Italy) 40 h prior to the experiments in order to induce differentiation and consequently to increase α_5_β_1_ integrin expression. HEK293 cells were routinely cultured in EMEM (Cambrex, Walkersville, MD, U.S.A.) with the addition of l-glutamine, nonessential amino acids, and 10% FBS. Cells were kept at 37 °C under 5% CO_2_ humidified atmosphere. All cell lines were obtained from American Type Culture Collection (ATCC, Rockville, MD, USA). The cell lines employed in this study are considered as useful in vitro models to investigate potential ligands acting as integrin agonists or antagonists.^32,34,64^

### Cell Adhesion Assays

The adhesion assays were performed as previously described.^32^ Briefly, regarding adhesion assays on Jurkat E6.1 cell, black 96-well plates (Corning Costar, Celbio, Milan, Italy) were coated overnight at 4 °C with VCAM-1 (5 μg/mL) to investigate α_4_β_1_ integrin-mediated cell adhesion. Jurkat E6.1 cells were stained by incubation with CellTracker green CMFDA (12.5 μM, 30 min at 37 °C, Life Technologies). After three washes, various concentrations of each compound (10^–4^ – 10^–10^ M) or the vehicle (methanol) were added to Jurkat E6.1 cells and incubated for 30 min at 37 °C. Cells were then plated (500 000 cells/well) on VCAM-1-coated wells and incubated for 30 min at 37 °C. After three washes, adhered cells were lysed with 0.5% Triton X-100 in PBS for 30 min at 4 °C, and green fluorescence was measured (Ex485 nm/Em535 nm).

For the adhesion assay on K562 and HT-29 cells, clear 96-well plates were coated by passive adsorption with fibronectin (10 μg/mL) overnight at 4 °C. K562 cells were then preincubated with various concentrations of each compound (10^–4^–10^–10^ M) or with the vehicle (methanol) for 30 min at room temperature. Then the cells (50 000 cells/well) were plated and incubated at room temperature for 1 h. After nonadherent cells were washed with 1% BSA (bovine serum albumin) in PBS, 50 μL of hexosaminidase substrate was added and incubated for 1 h at room temperature. After the addition of stopping solution, plates were read at 405 nm in an EnSpire Multimode Plate Reader (PerkinElmer, Waltham, MA, USA).

Experiments were carried out in quadruplicate and repeated at least three times. Data analysis and IC_50_ or EC_50_ values were calculated using GraphPad Prism 5.0 (GraphPad Software, San Diego, CA, U.S.A.).

### Cellular Uptake

Intracellular uptake of fluorescent-conjugated compounds was evaluated by flow cytometry as previously described,^32^ with the following modifications. Jurkat, K562, and HEK293 cells were seeded in 12-well plates and treated with fluorescent conjugates (1–10–25 μM) for 1 h at 37 °C. To determine integrin involvement in fluorescent conjugates cell internalization, cells were pretreated with anti-α^4^ (10 μg/mL, Abcam, #Ab220) or anti-α_5_ (10 μg/mL, BD Bioscience, #555651) antibody or α_4_β_1_ selective agonist **A** (100 μM) for 30 min. Afterward, the cells were washed three times with cold PBS, and cellular uptake was quantified by flow cytometry on a Guava easyCyte 5 flow cytometer (Merck Millipore, Vimodrone, Italy).

### Confocal Laser Scanning Microscopy

HEK293 cells (not expressing α_5_ nor α_4_ but endogenously expressing β_1_ integrin)^65,66^ were plated in 6-well plates on glass coverslip and transiently transfected with α_5_ (pCB7 alpha5) or with α_4_ (pcDNA3.1+ α_4_, Origene, Rockville, MD, USA) subunit coding plasmid. After 48 h from transfection, the expression of α_5_ or α_4_ subunit was verified by flow cytometry (data not shown). pCB7 alpha5 was a gift from Filippo Giancotti (Addgene plasmid #16041).^67^ HEK293 cells were treated with compound **B** or **C** (1 μM) for 1 h. Afterward, the cells were washed twice with PBS and fixed with paraformaldehyde (3% in PBS, pH 7.4, 10 min); then the coverslips were washed twice with 0.1 M glycine in PBS and twice with 1% BSA (bovine serum albumin) in PBS. Nuclei were counterstained with 4′,6-diamidino-2-phenylindole dilactate (DAPI, Sigma). Specimens were embedded in Mowiol and analyzed using a Nikon C 1s confocal laser-scanning microscope, equipped with a Nikon PlanApo 60×, 1.4-NA oil immersion lens.

### Cell Apoptosis Detection

Phycoerythrin-conjugated annexin V (annexin-PE) and 7-amino-actynomicin D (7-AAD; Guava Nexin Reagent, Merck Millipore, Darmstadt, Germany) were employed to determine the percentage of viable, early apoptotic, and late apoptotic/necrotic cells by flow cytometry.^32,68^ After 72 h treatments with different concentrations of compounds (10–50–100 μM), cells were collected by centrifugation, the supernatants were discarded, and the cell pellets were resuspended in 100 μL of complete medium. Then, the cells were stained with 100 μL of Nexin reagent for 20 min at room temperature in the dark, following the manufacturer’s instructions. Cells were analyzed on a Guava easyCyte 5 flow cytometer. At least 10 000 cells/sample were analyzed. Three populations of cells can be identified by this assay: viable cells (annexin V-PE and 7-AAD negative), early apoptotic cells (annexin V-PE positive and 7-AAD negative), and late stages apoptosis or necrotic cells (annexin V-PE and 7-AAD positive). Sample acquisition and data analysis were performed using the InCyte software module.

### Caspase 3/7 Activation

Apoptosis was further assayed by measuring caspase-3/7 activity following treatment with 5-FU conjugated compounds using Guava Caspase 3/7 FAM kit (Millipore) according to the manufacturer’s instructions. Briefly, cells were exposed to different concentrations of compounds (10–50–100 μM) for 72 h; then cells were collected by centrifugation, the supernatants were discarded, and the cell pellets were resuspended in 100 μL of complete medium. Then, the cells were stained with 100 μL of Caspase 3/7 reagent working solution for 60 min at 37 °C. At the end of the incubation cells were washed twice with 1× Apoptosis Wash Buffer and stained with 7-AAD reagent for 10 min at room temperature. Cells were analyzed on a Guava easyCyte 5 flow cytometer. At least 10 000 cells/sample were analyzed. Three populations of cells can be identified in this assay: viable cells (negative for both caspase 3/7 and 7-AAD reagents); cells in the middle stages of apoptosis (positive for caspase 3/7 reagent and negative for 7-AAD); cells in the late stages of apoptotic or dead (positive for both caspase 3/7 and 7-AAD reagents). Sample acquisition and data analysis were performed using the InCyte software module.

## Supporting Information Available

Figure S1, stability tests, HPLC-MS analysis and spectral characterizations (^1^H, ^13^C NMR) (PDF)

## ABBREVIATIONS

THF: tetrahydrofuran
TEA: triethylamine
DCC: dicyclohexylcabodiimide
EDC: 1-ethyl-3-(3-(dimethylamino)propyl)carbodiimide
DMAP: *N,N-*dimethylaminopyridine
DMF: *N,N*-dimethylformamide
HOBt: hydroxybenzotriazole
5-FU: 5-fluorouracil
TFA: trifluoroacetic acid
FITC: fluorescein isothiocyanate
PBS: phosphate buffer
FBS: fetal bovine serum
BSA: bovine serum albumin
CMFDA: 5-chloromethylfluorescein diacetate
FN: fibronectin
VCAM-1: vascular cell adhesion molecule-1
PE: phycoerythrin
7-AAD: 7-aminoactinomycin D
hMSC: human mesenchymal stem cells
MIDAS: metal ion-dependent adhesion site
MFI: mean fluorescent intensity
PMA: phorbol 12-myristate 13-acetate
