# Supplementary material for: Selective Integrin Ligands Promote Cell Internalization of the Antineoplastic Agent Fluorouracil
Source: ACS Pharmacol Transl Sci. 2021 Sep 2;4(5):1528–42. doi: 10.1021/acsptsci.1c00094 (PMC8506610; doi:10.1021/acsptsci.1c00094)

Supporting Information

Selective Integrin Ligands Promote Cell Internalization of the Antineoplastic Agent Fluorouracil

Monica Baiula, ‡* Martina Cirillo, † Giulia Martelli,† Valentina Giraldi,† Elisa Gasparini,† Alessandro Claudio Anelli,† Santi Mario Spampinato,‡ and Daria Giacomini†*

‡ Department of Pharmacy and Biotechnology, University of Bologna, Via Irnerio, 48, 40126, Bologna, Italy

† Department of Chemistry "G. Ciamician", University of Bologna, Via Selmi 2, 40126 Bologna, Italy.

E-mails of corresponding authors: monica.baiula@unibo.it; daria.giacomini@unibo.it

Content:

- Figure S1 Confocal microscopy images **page S2**
- Stability tests for compounds D, E, and F in aqueous phosphate buffer PBS (pH = 7.4), in fetal bovine serum (FBS), and for compounds E and F in aqueous phosphate buffer PBS (pH = 6.0) **pages S3-S10**
- HPLC-MS analyses of stability tests samples of compounds D, E, F in FBS **page S11-S13**
- ^{1}H NMR and ^{13}CNMR spectra for compounds B, C, D, E, F, and 2-12 **pages S14-S29**
- HPLC analysis of compounds B, C, D, E, and F **page S30**
- ^{1}H NMR and HPLC-MS of compound **15** **page S31**

S1

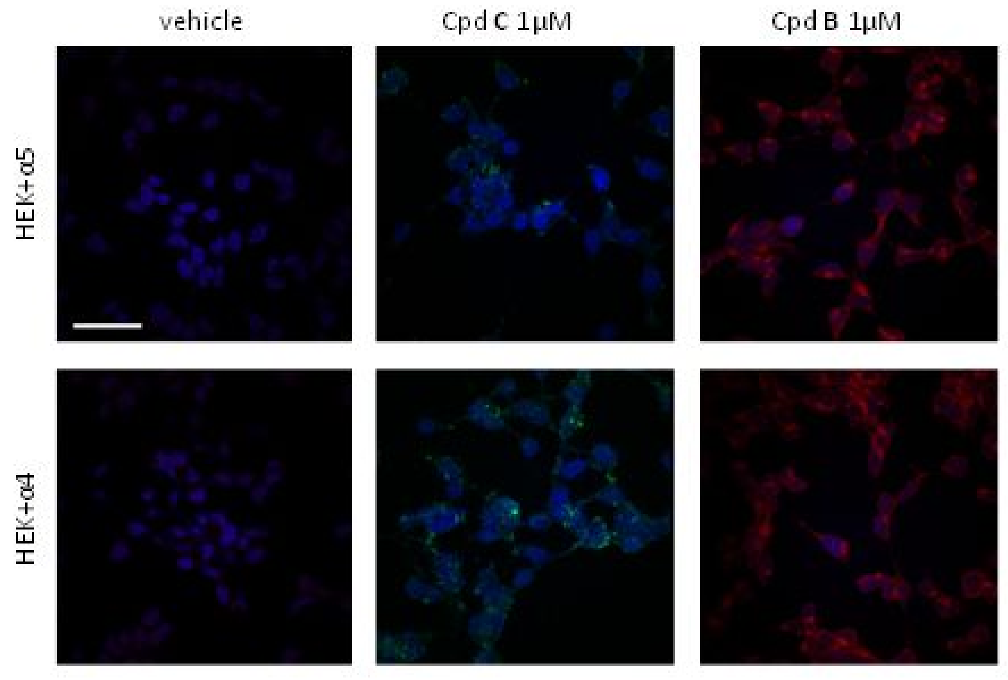

Figure S1. Confocal microscopy images of HEK293+α_4 or HEK293+α_5 cells treated with compound **C** (Cpd **C**, 1 µM; green fluorescence) or compound **B** (Cpd **B**, 1 µM; red fluorescence) or their vehicle for 1 h. Nuclei were counterstained with DAPI. Scale bar: 30 µm. The images were elaborated using NIS-Elements C Software.

Stability tests for compound D

Data for Compound **D** in PBS (pH = 7.4)

<u>HPLC analysis</u>: Zorbax-Eclipse column XDB – C18 4.6x150 mm 5 micron, flow 0,5 mL/min, 30°C

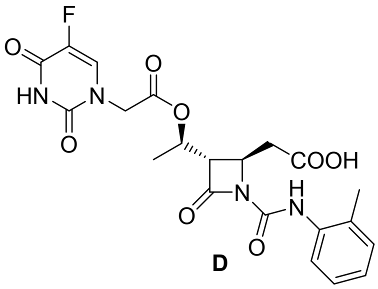

D

Incubation in buffer phosphate (PBS) 0.1M pH = 7.4 at 30°C

Concentration of **D** at time 0 C_0 = 1mg/ml
Retention time = 13 min (HPLC elution conditions: from 60% H_2O + formic acid 0.2% - 40% MeCN + formic acid 0.2% to 90 % H_2O + formic acid 0.2% - 10% MeCN + formic acid 0.2% in 30 min).

Table S1. HPLC data

Time (h)	Area (mAuxs) λ=254 nm	mol/mol_0 %
0	472872	100
1	479768	101
2	476121	101
4	467288	99
5	463779	98
7	463975	98
24	429891	91
48	378227	80
72	373332	79

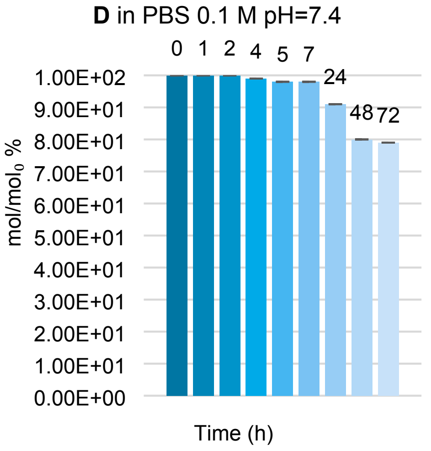

Data for Compound **D** in FBS

HPLC analysis: Gemini column – C18 100x2 mm 3 micron, flow 0,5 mL/min, 30°C

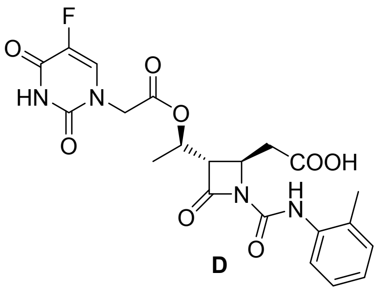

D

Incubation in Fetal Bovine Serum (FBS) at 30°C

Concentration of **D** at time 0 C_0 = 1mg/ml
Retention time = 6 min (HPLC elution conditions: 60% H_2O + TFA 0.08% - 40% MeCN + TFA 0.08% to 80 % H_2O + TFA 0.08% - 20% MeCN + TFA 0.08% in 25 min).

Table S2. HPLC data

Time (h)	Area (mAuxs) λ=254 nm	mol/mol_0 %
0	7036	100
1	7231	103
2	6690	95
4	5984	85
7	5810	83
24	5369	76
48	4923	70
72	4288	61

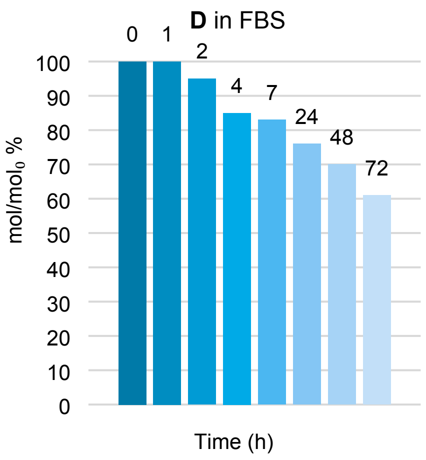

Stability tests for compound E

Data for Compound **E** in PBS (pH = 7.4)

<u>HPLC analysis</u>: Zorbax-Eclipse column XDB – C18 4.6x150 mm 5 micron, flow 0.5 mL/min, 30°C

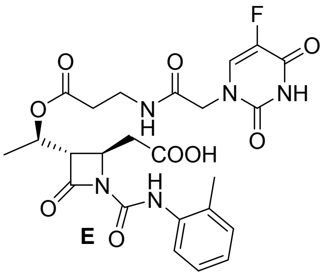

Incubation in buffer phosphate (PBS) 0.1M pH = 7.4

Concentration of **E** at time 0 C_0 = 1mg/ml
Retention time = 13 min (HPLC elution conditions: from 60% H_2O + formic acid 0.2% - 40% MeCN + formic acid 0.2% to 90 % H_2O + formic acid 0.2% - 10% MeCN + formic acid 0.2% in 30 min).

Table S3. HPLC data

Time (h)	Area (mAuxs) λ=254 nm	mol/mol$_0$ %
0	462855	100
1	449416	97
2	451766	98
5	448138	97
7	452891	98
24	434403	94
72	413465	89

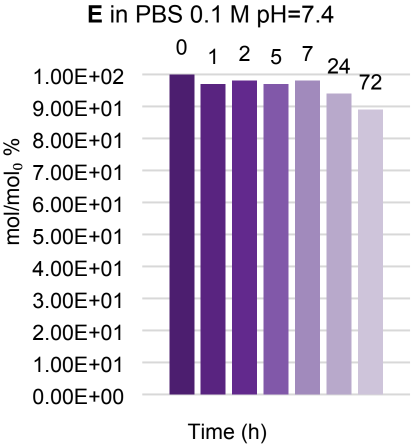

Data for Compound **E** in PBS (pH = 6)

HPLC analysis: InfinitiLab Poroshell 120 EC-C18 3.0x150 mm 2.7 micron, flow 0.3 mL/min, 40°C

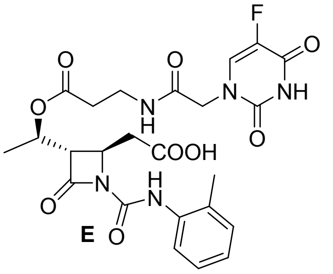

E

Incubation in buffer phosphate (PBS) 0.1M pH = 6 at 30°C

Concentration of **E** at time 0 C_0 = 1 mg/ml
Retention time = 5.4 min (HPLC elution conditions: from 70% H_2O + TFA 0.1% - 30% MeCN + TFA 0.1% to 10 % H_2O + TFA 0.1% - 90% MeCN + TFA 0.1% in 24 min).
Standard dev: 42 mAuxs

Table S4. HPLC data

Time (h)	Area (mAuxs) λ=254 nm	mol/mol$_0$ %
0	4995	100
1	5032	101
2	5055	101
4	5180	104
7	5445	109
24	5062	101
48	4988	99.9
72	4975	99.6

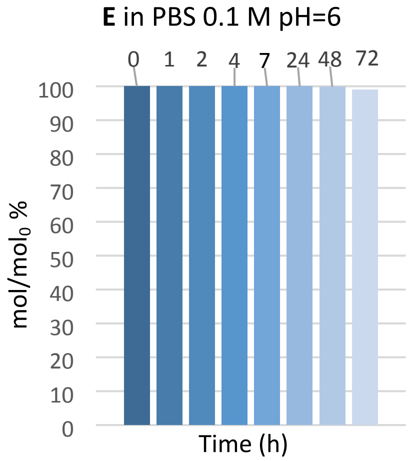

Data for Compound **E** in FBS

<u>HPLC analysis:</u> Gemini column – C18 100x2 mm 3 micron, flow 0,5 mL/min, 30°C

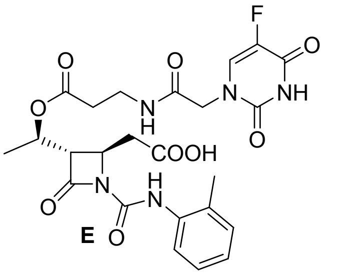

E

Incubation in Fetal Bovine Serum (FBS) at 30°C

Concentration of **E** at time 0 C_0 = 1mg/ml
Retention time = 4 min (HPLC elution conditions: 60% H_2O + TFA 0.08% - 40% MeCN + TFA 0.08% to 90 % H_2O + TFA 0.08% - 10% MeCN + TFA 0.08% in 30 min).

Table S5. HPLC data

Time (h)	Area (mAuxs) λ=254 nm	mol/mol_0 %
0	6335	100
1	6786	107
2	6691	106
5	6783	107
7	6056	96
48	5513	87
72	4930	78

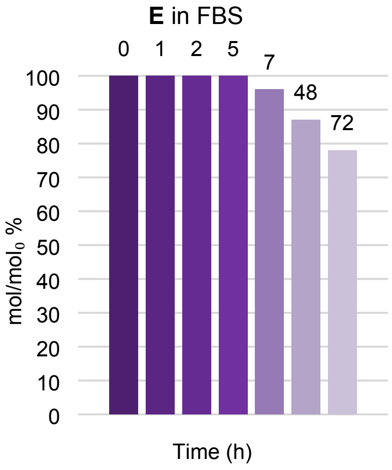

Stability tests for compound F

Data for Compound **F** in PBS (pH = 7.4)

HPLC analysis: Gemini column – C18 100x2 mm 3 micron, flow 0,4 mL/min, 30°C

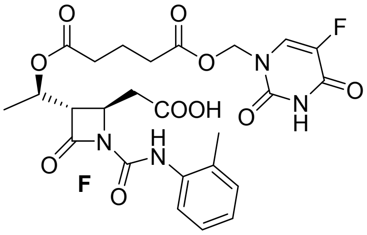

Incubation in buffer phosphate (PBS) 0.1M pH = 7.4 at 30°C

Concentration of **F** at time 0 C_0 = 1mg/ml
Retention time = 3 min (HPLC elution conditions: 20% H_2O + TFA 0.08% - 80% MeCN + TFA 0.08% to 10 % H_2O TFA 0.08% - 90% MeCN + TFA 0.08% in 25 min).

Table S6. HPLC data

Time (h)	Area (mAuxs) λ=280 nm	mol/mol$_0$%
0	230749	100
1	232005	101
3	242890	105
4	241960	105
24	213893	93
48	204333	89
72	189093	82

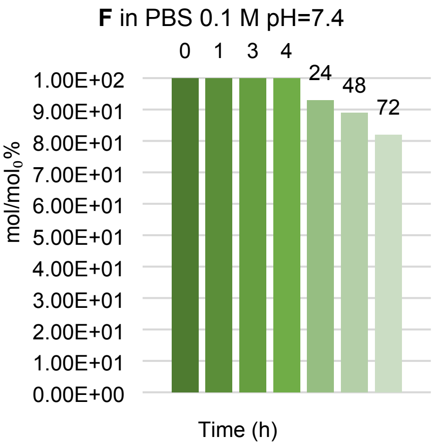

Data for Compound **F** in PBS (pH = 6)

HPLC analysis: InfinitiLab Poroshell 120 EC-C18 3.0x150 mm 2.7 micron, flow 0.3 mL/min, 40°C

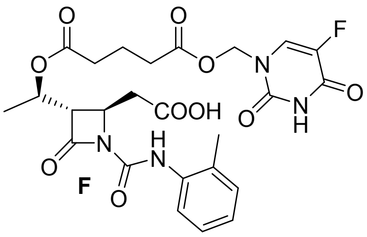

Incubation in buffer phosphate (PBS) 0.1M pH = 6 at 30°C

Concentration of **E** at time 0 C_0 = 1 mg/ml
Retention time = 6.5 min (HPLC elution conditions: from 70% H_2O + TFA 0.1% - 30% MeCN + TFA 0.1% to 10 % H_2O + TFA 0.1% - 90% MeCN + TFA 0.1% in 24 min).
Standard dev: 42 mAuxs

Table S7. HPLC data

Time (h)	Area (mAuxs) λ=254 nm	mol/mol_0 %
0	4624	100
1	4623	100
2	4639	100
4	4629	100
7	4647	101
24	4570	99
48	4521	98
72	4528	98

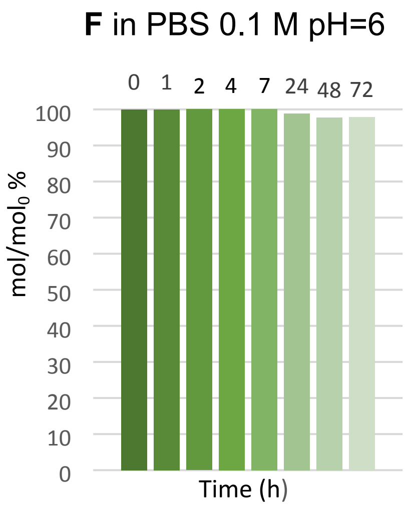

Data for Compound **F** in FBS

HPLC analysis: Gemini column – C18 100x2 mm 3 micron, flow 0,4 mL/min, 30°C

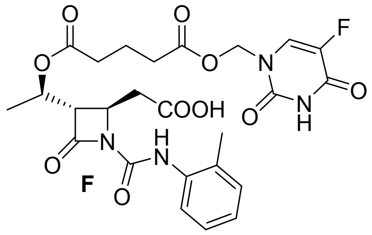

Incubation in Fetal Bovine Serum (FBS) at 30°C

Concentration of **F** at time 0 C_0 = 1mg/ml
Retention time = 3 min (HPLC elution conditions: Da 20% H_2O + TFA 0.08% - 80% MeCN + TFA 0.08% to 10 % H_2O TFA 0.08% - 90% MeCN + TFA 0.08% in 25 min).

Table S8. HPLC data

Time (h)	Area (mAuxs) λ=280 nm	mol/mol_0 %
0	6926	100
1	7429	107
3	7260	105
5	7534	109
24	5888	85
48	3729	54
72	220	32

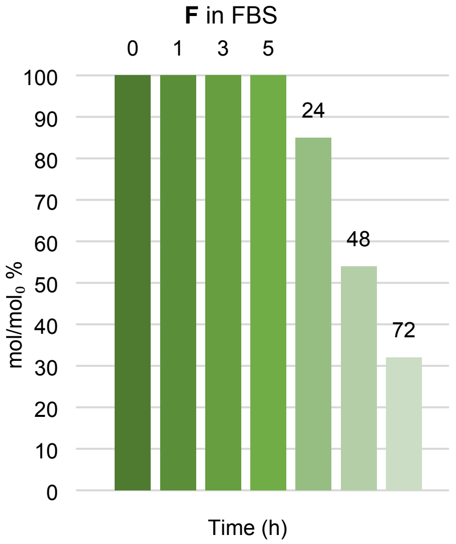

HPLC-MS analysis of compound **D** after 2 h in FBS

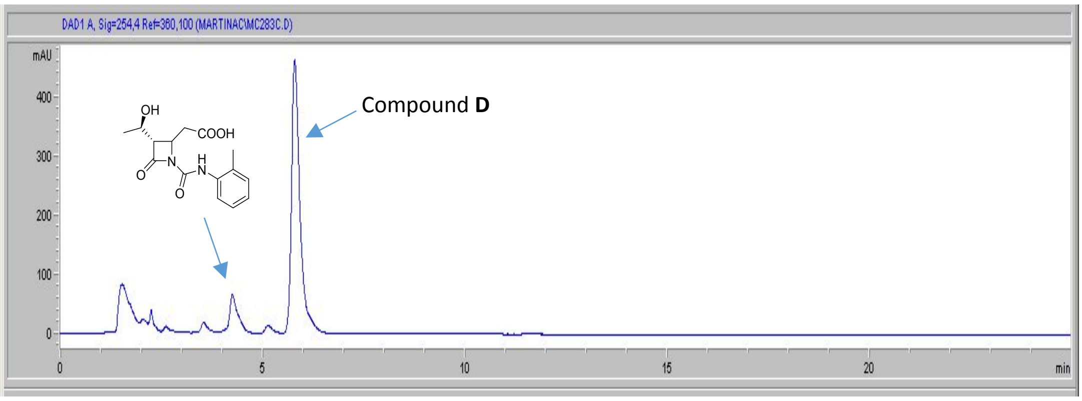

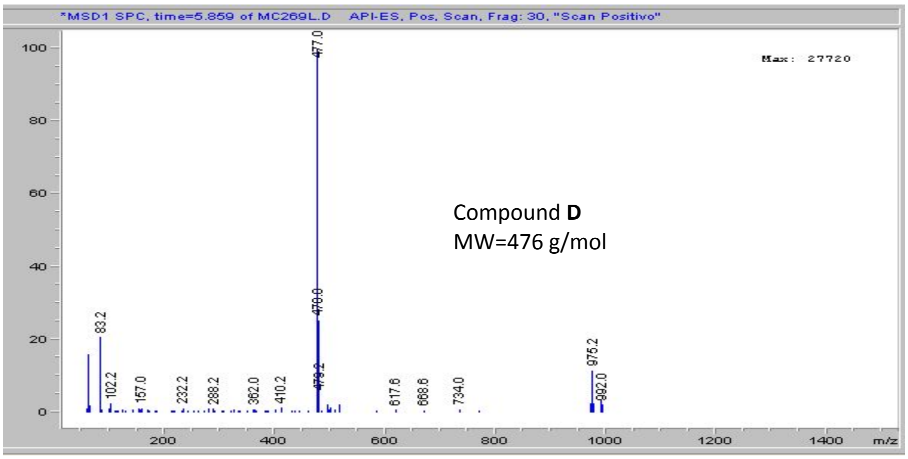

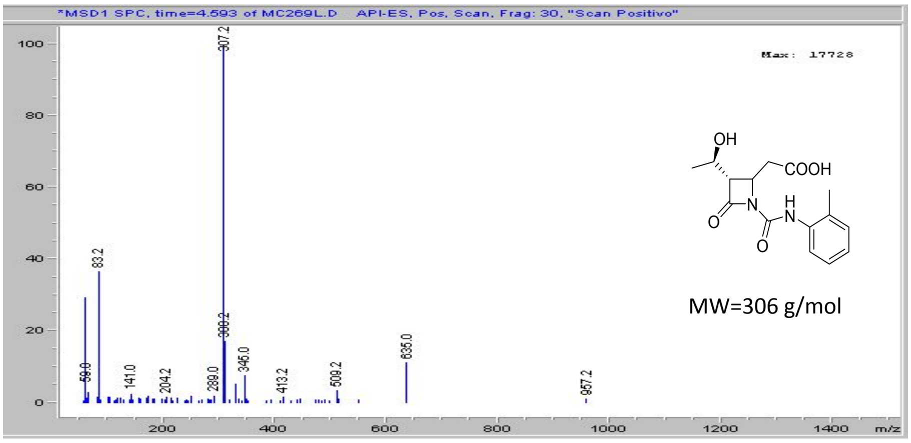

HPLC-MS analysis of compound **E** after 72 h in FBS

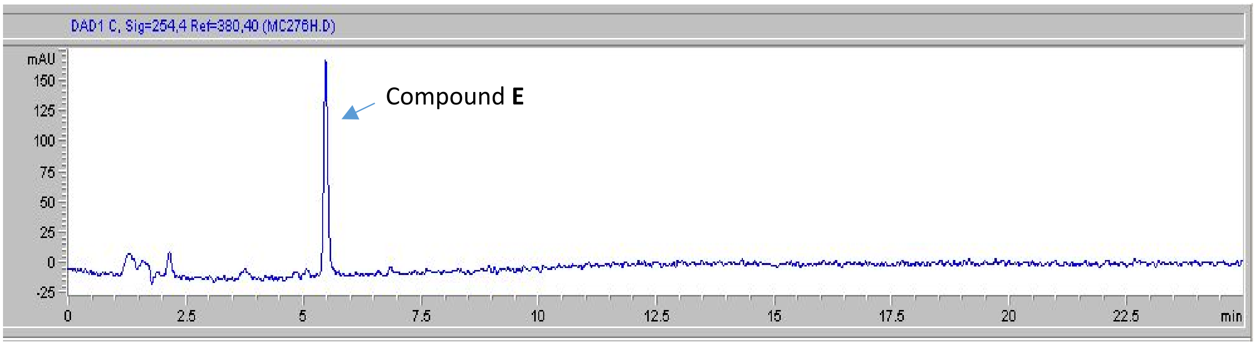

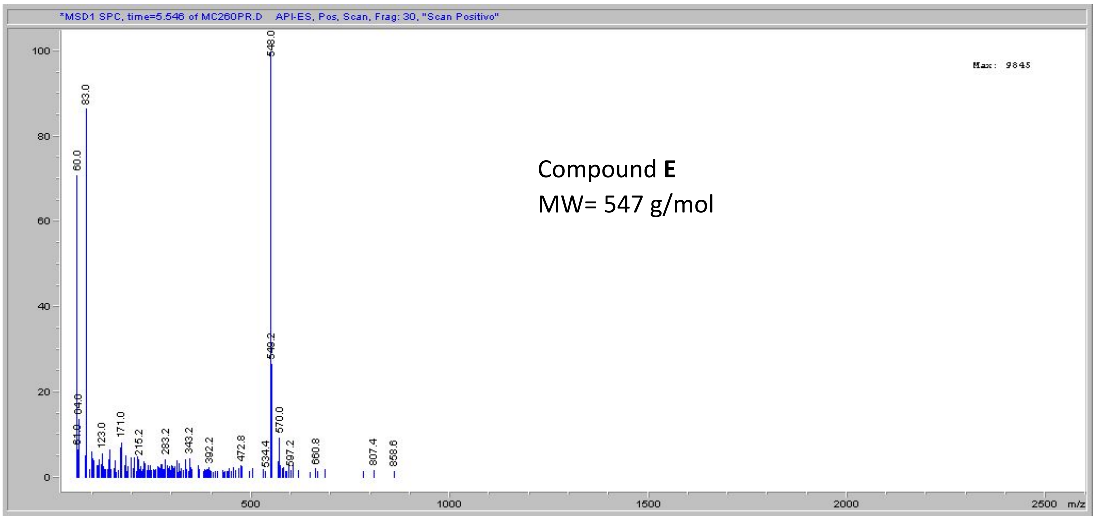

HPLC-MS analysis of compound **F** after 24 h in FBS

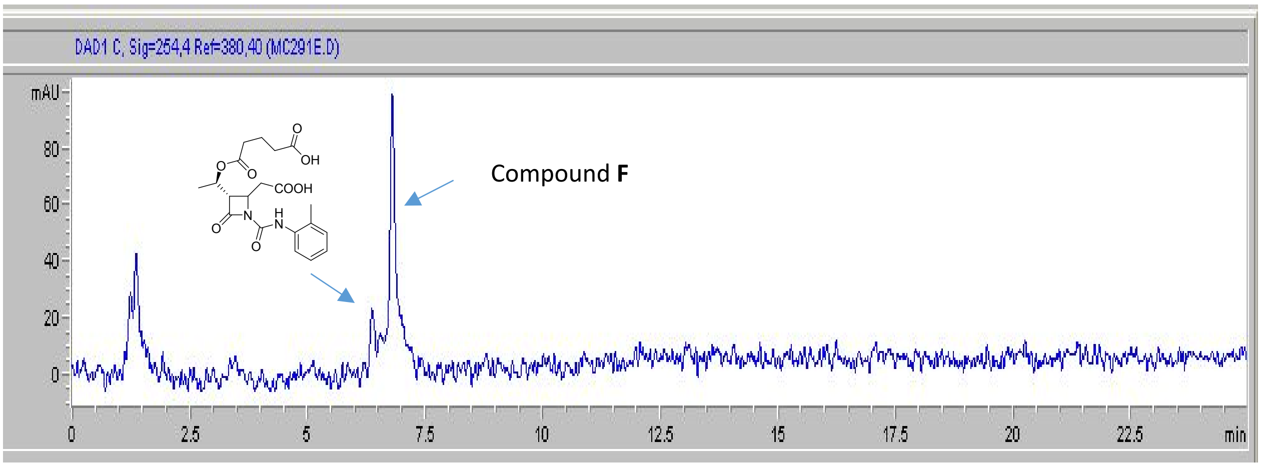

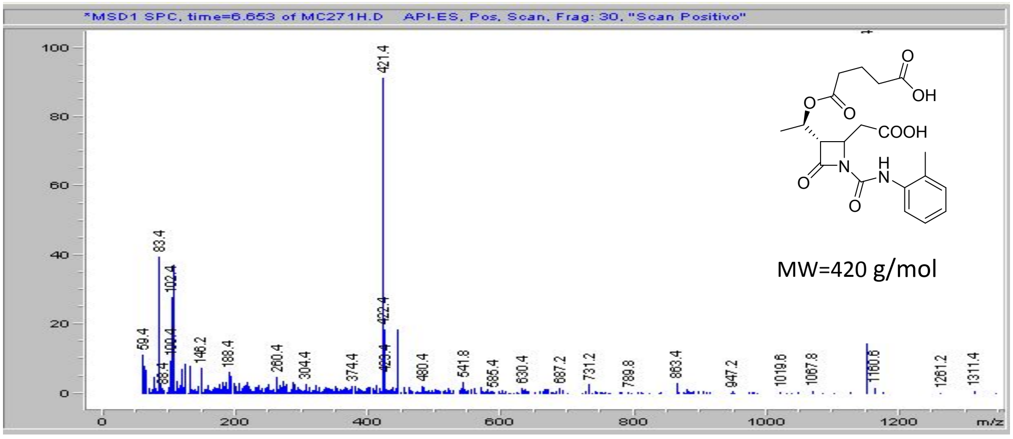

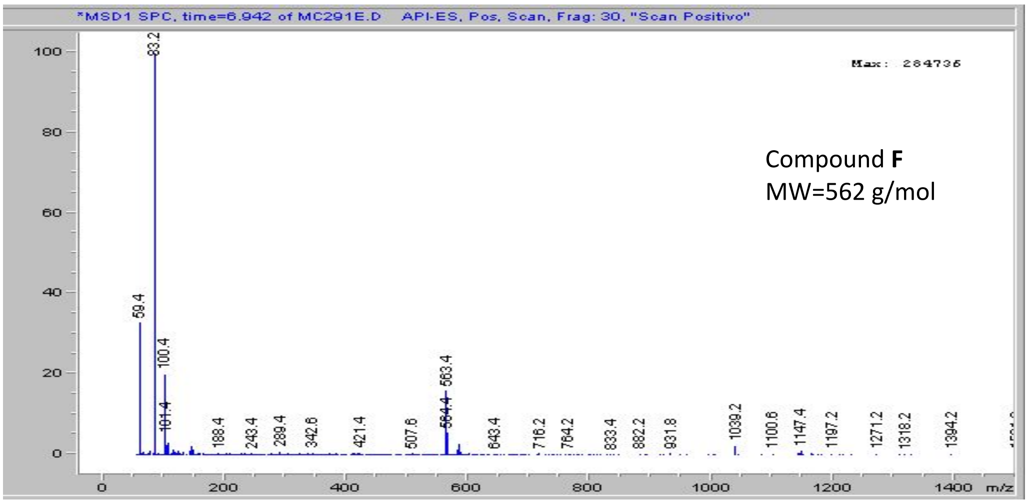

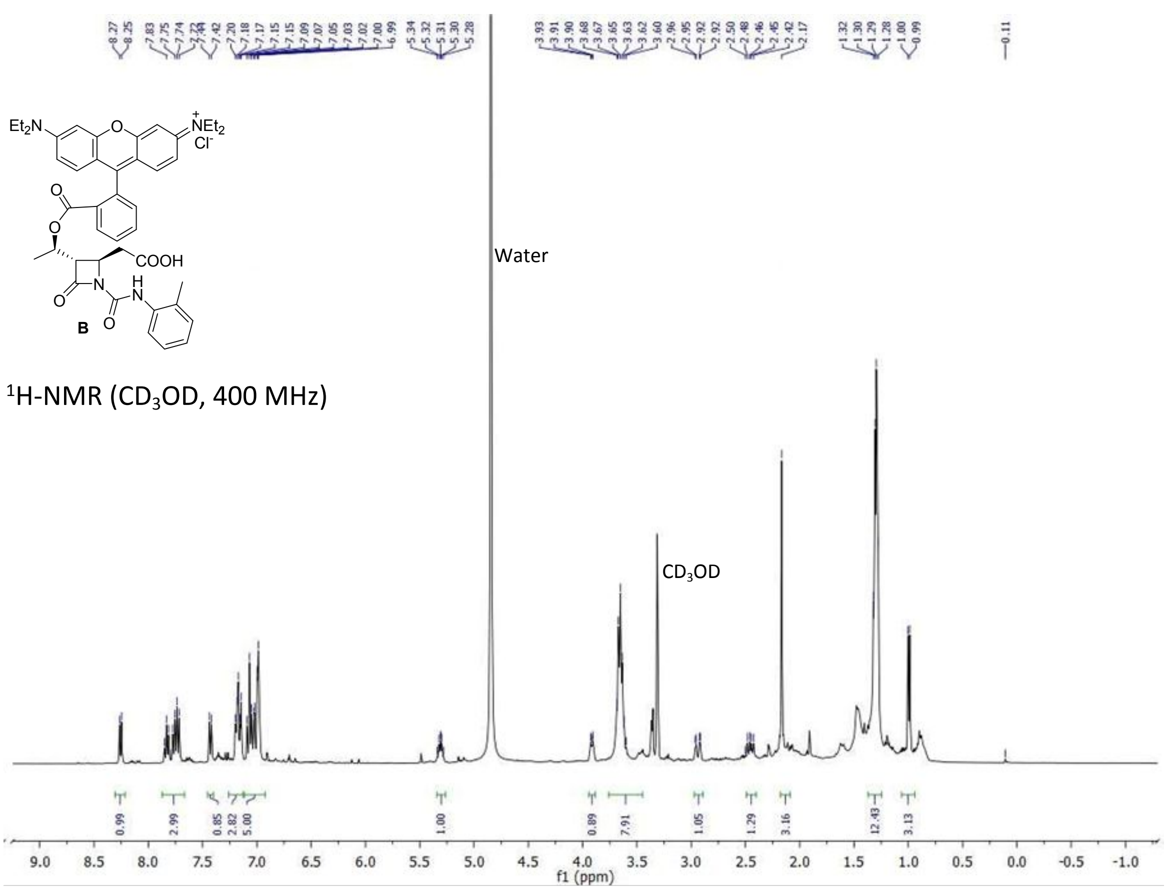

^{1}H-NMR (CD_3OD, 400 MHz)

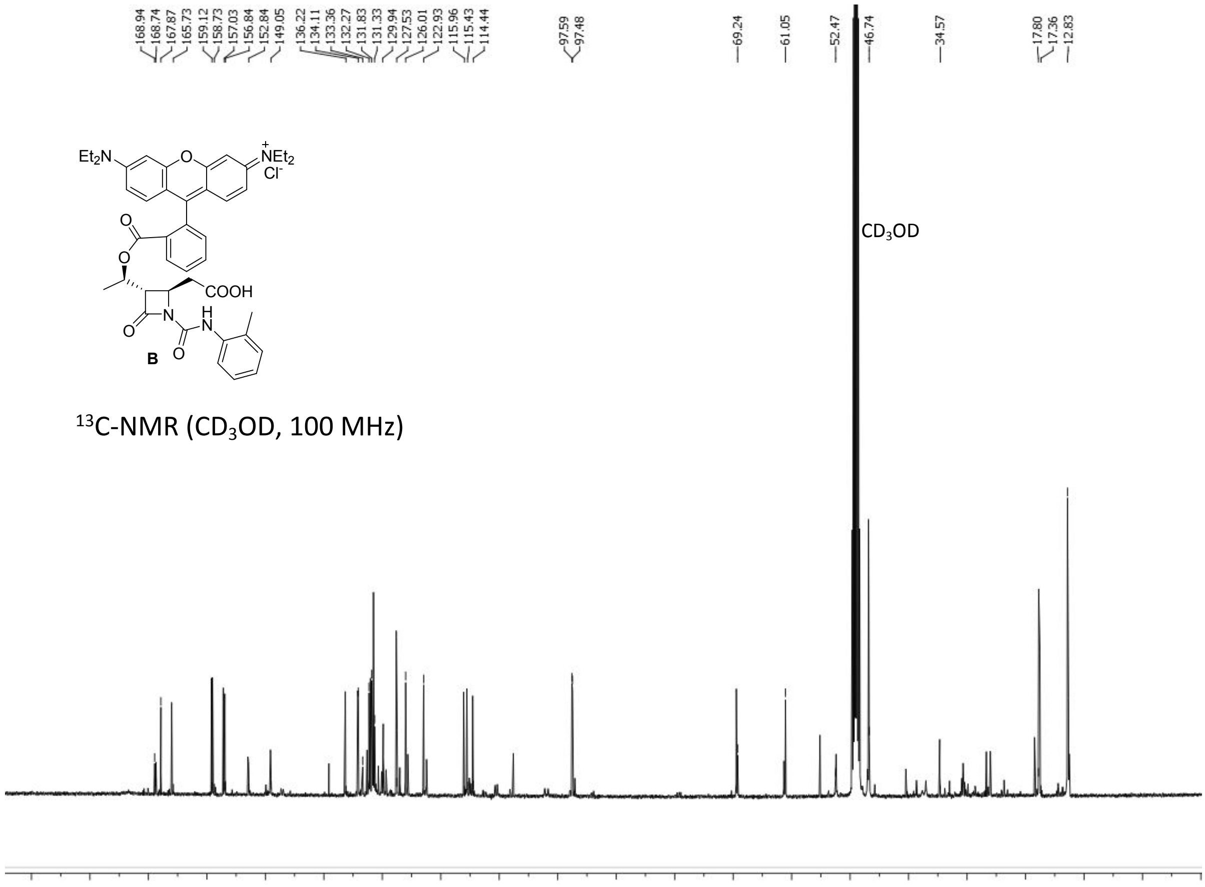

^{13}C-NMR (CD_3OD, 100 MHz)

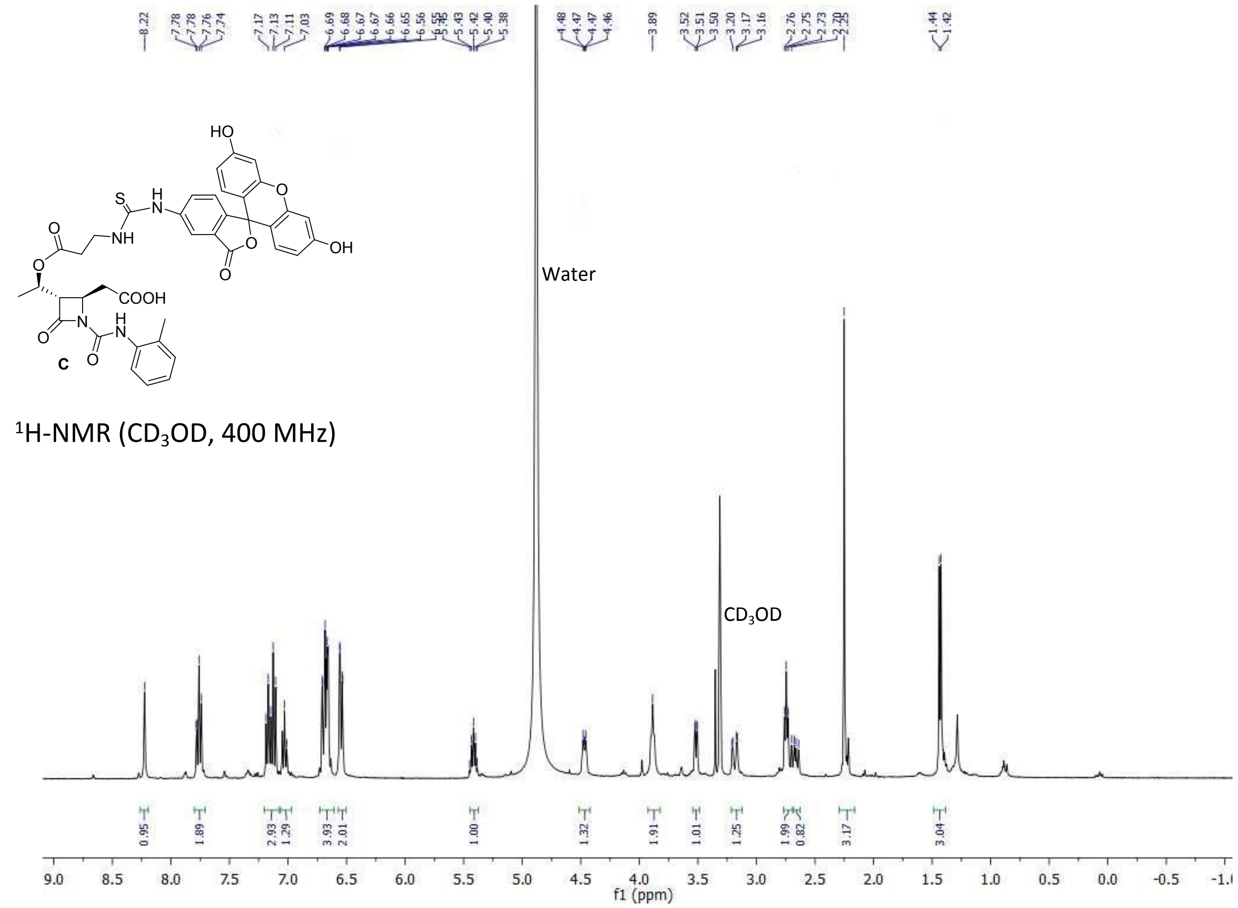

^{1}H-NMR (CD_3OD, 400 MHz)

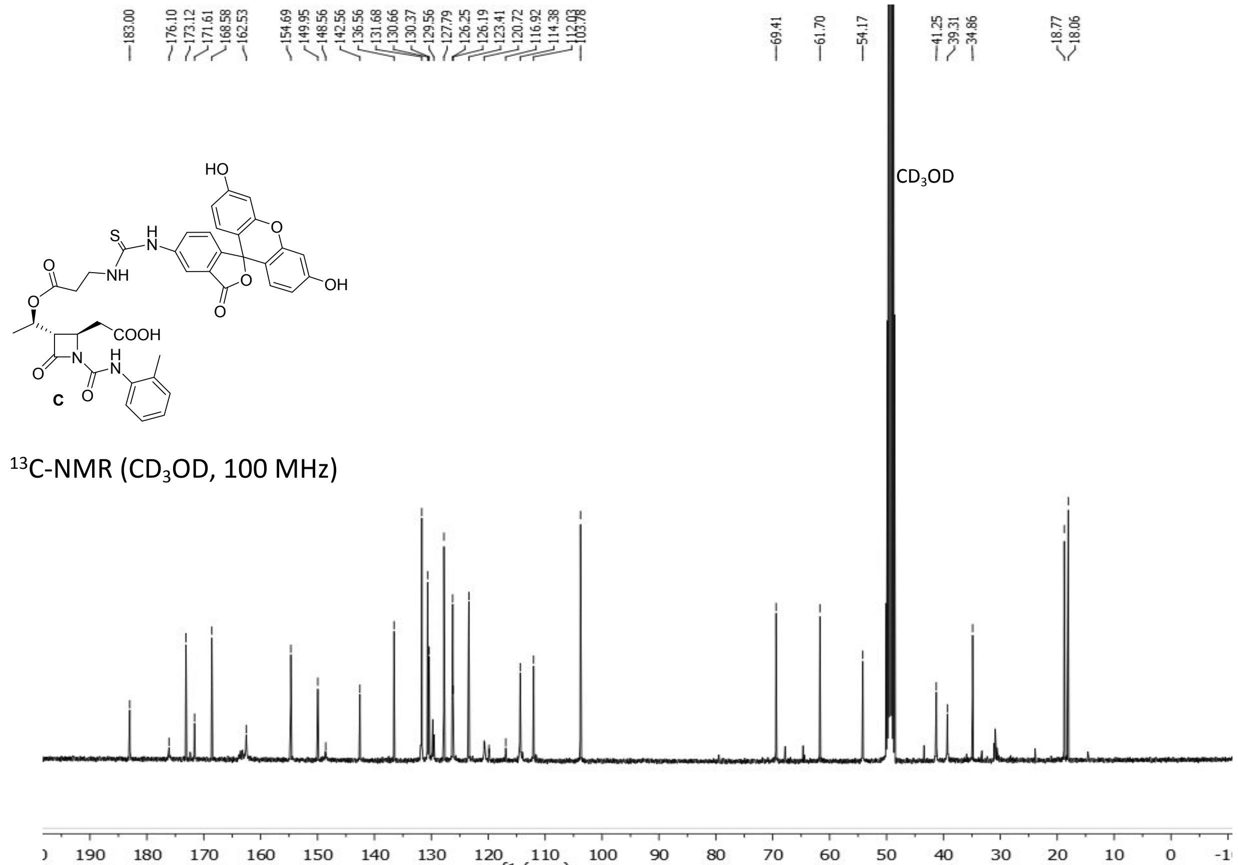

^{13}C-NMR (CD_3OD, 100 MHz)

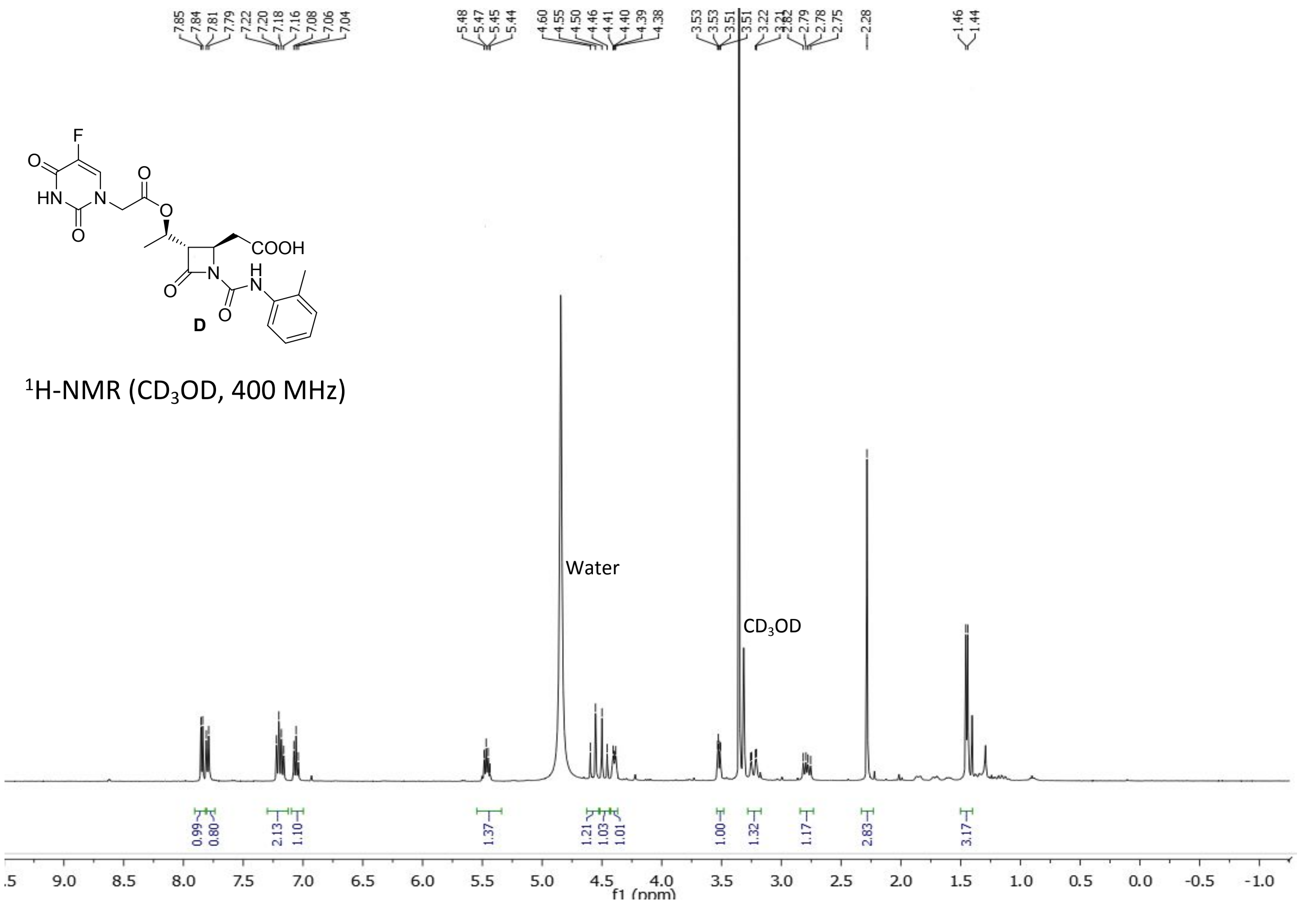

^{1}H-NMR (CD$_3$OD, 400 MHz)

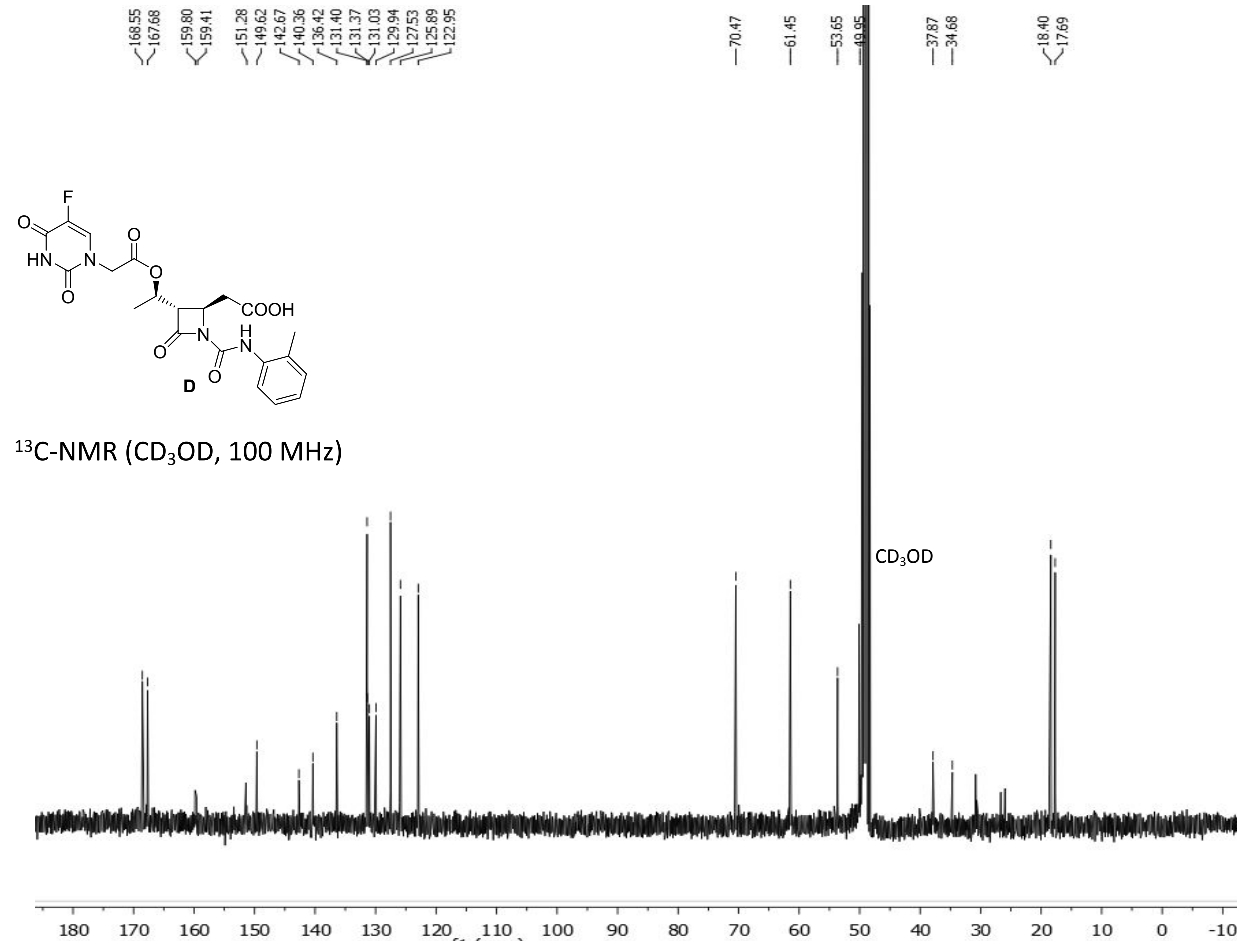

^{13}C-NMR (CD$_3$OD, 100 MHz)

^{1}H-NMR (CD$_3$OD, 400 MHz)

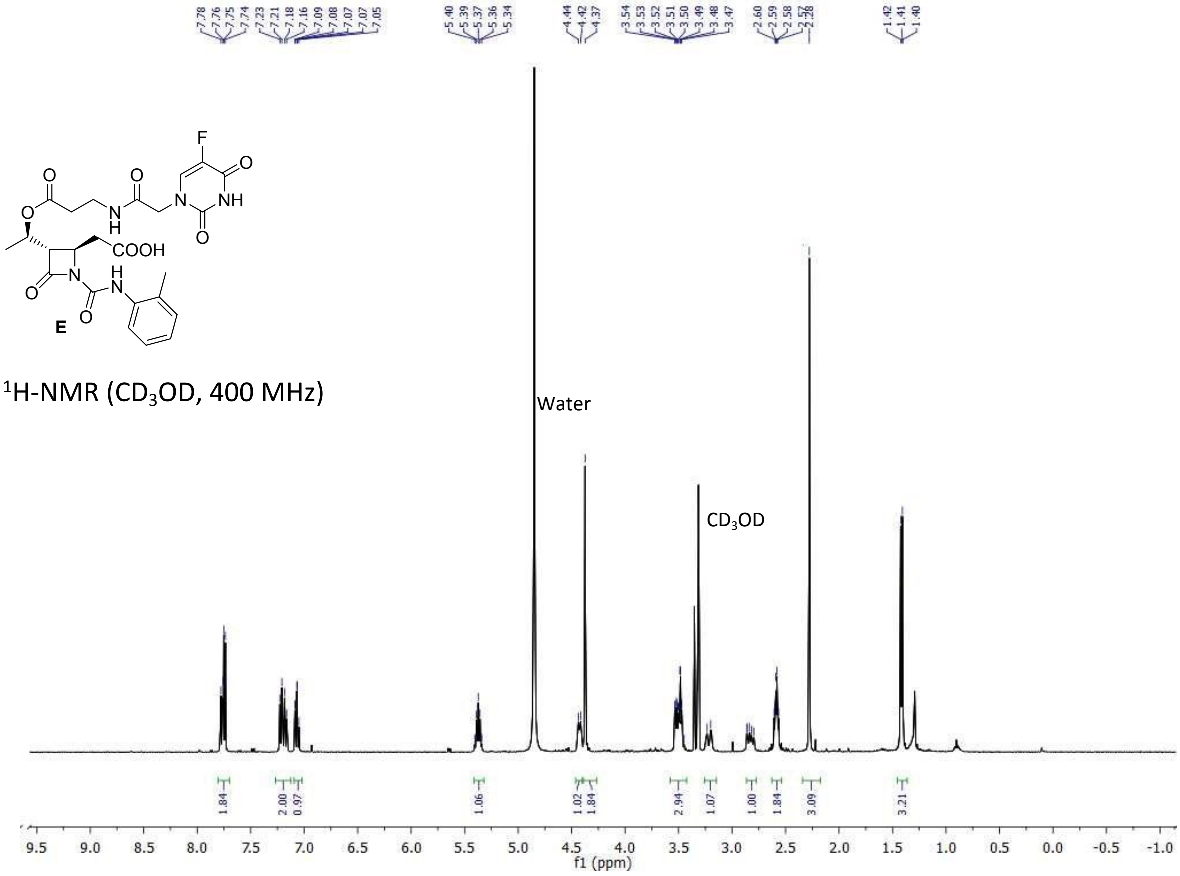

^{13}C-NMR (CD$_3$OD, 100 MHz)

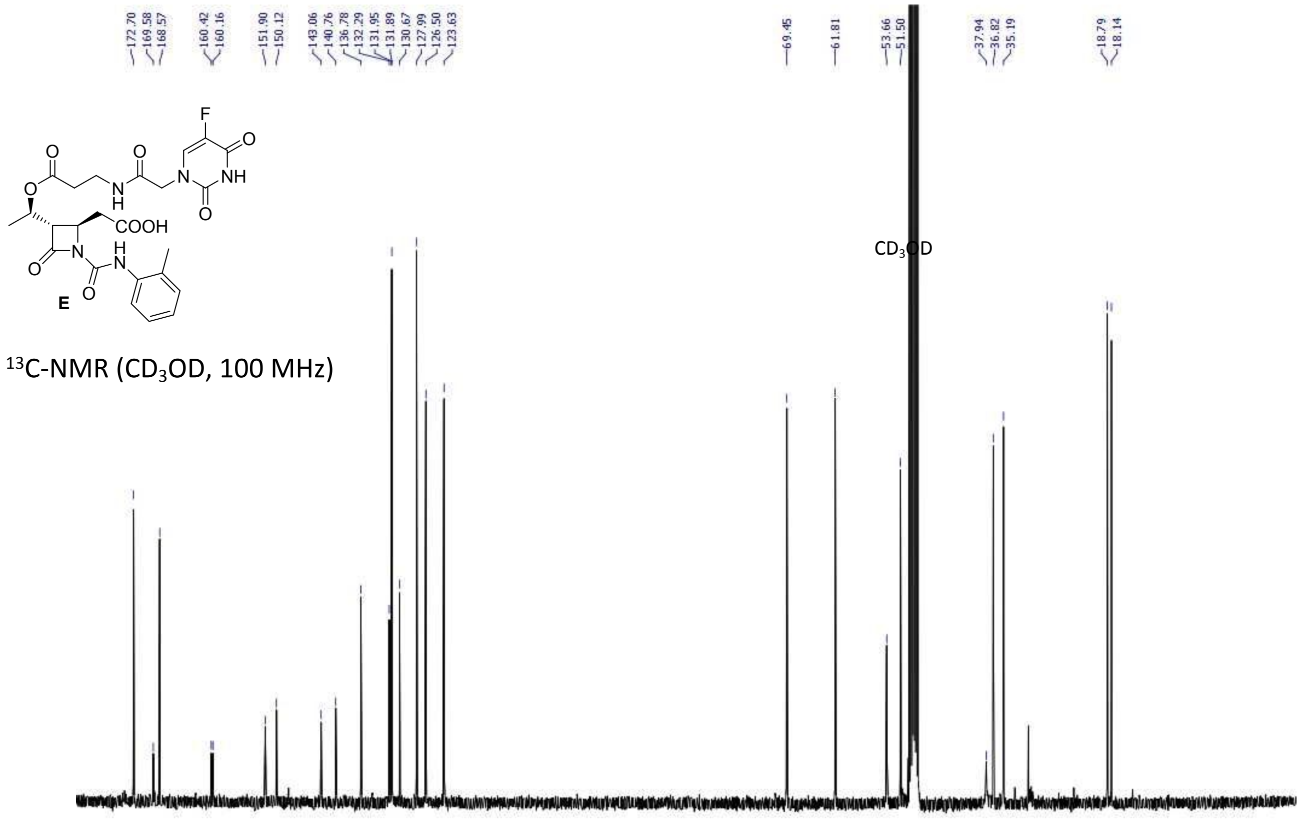

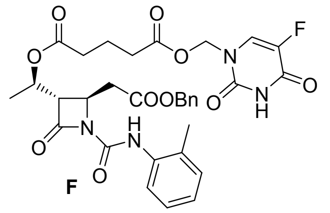

^{1}H-NMR ($CDCl_3$, 400 MHz)

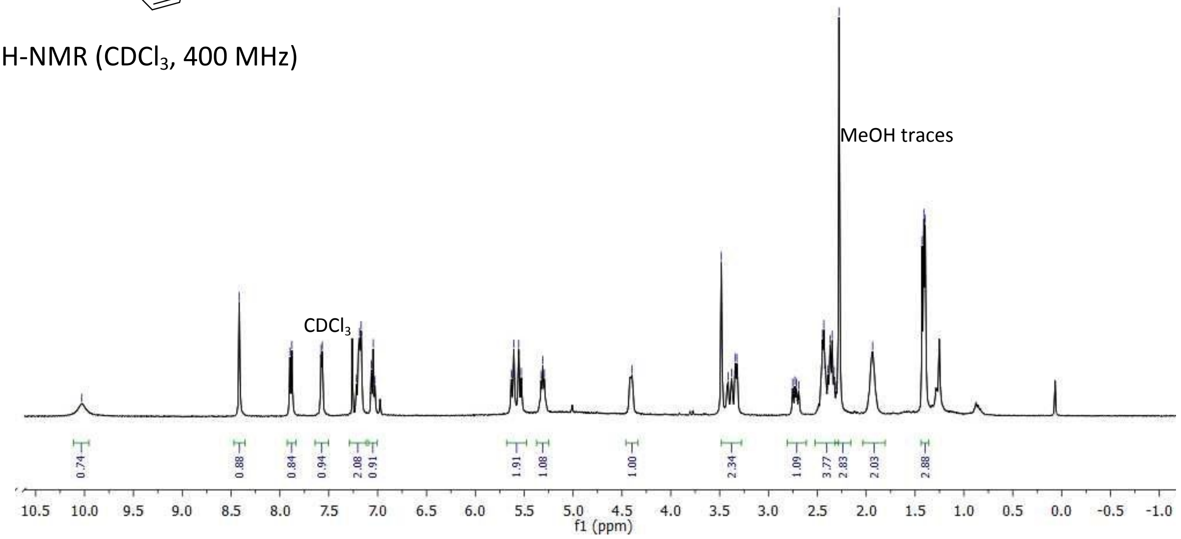

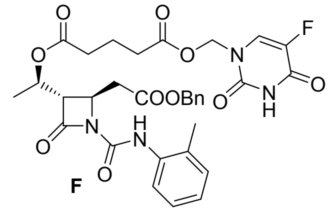

^{13}C-NMR ($CDCl_3$, 100 MHz)

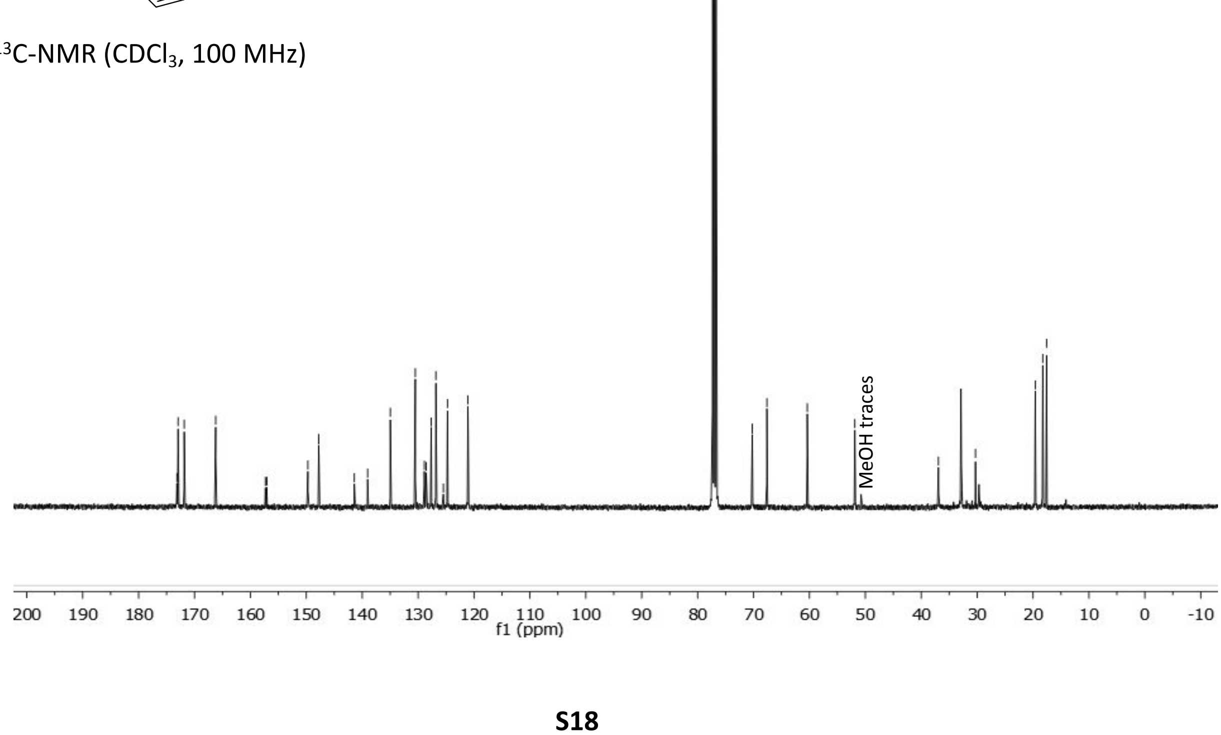

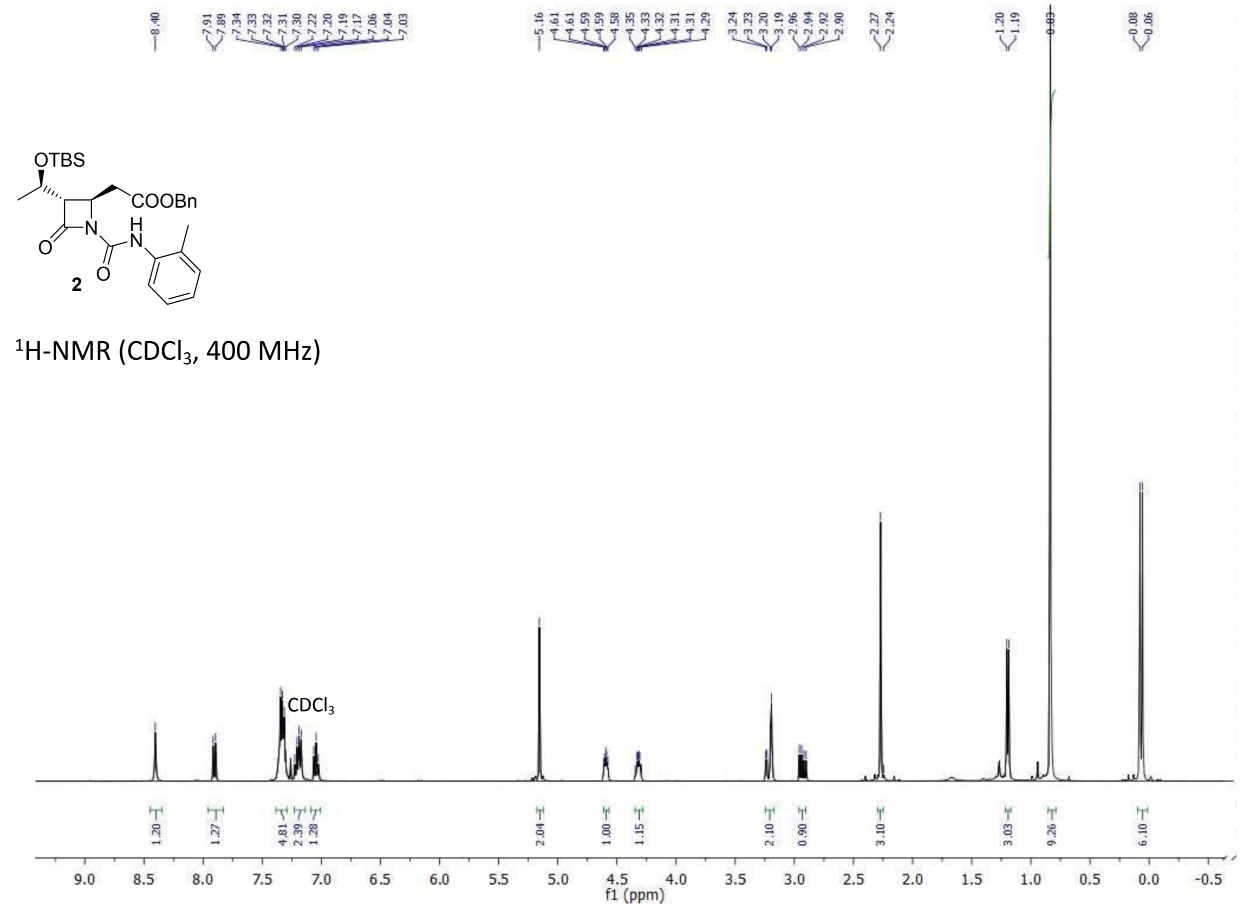

^{1}H-NMR ($CDCl_3$, 400 MHz)

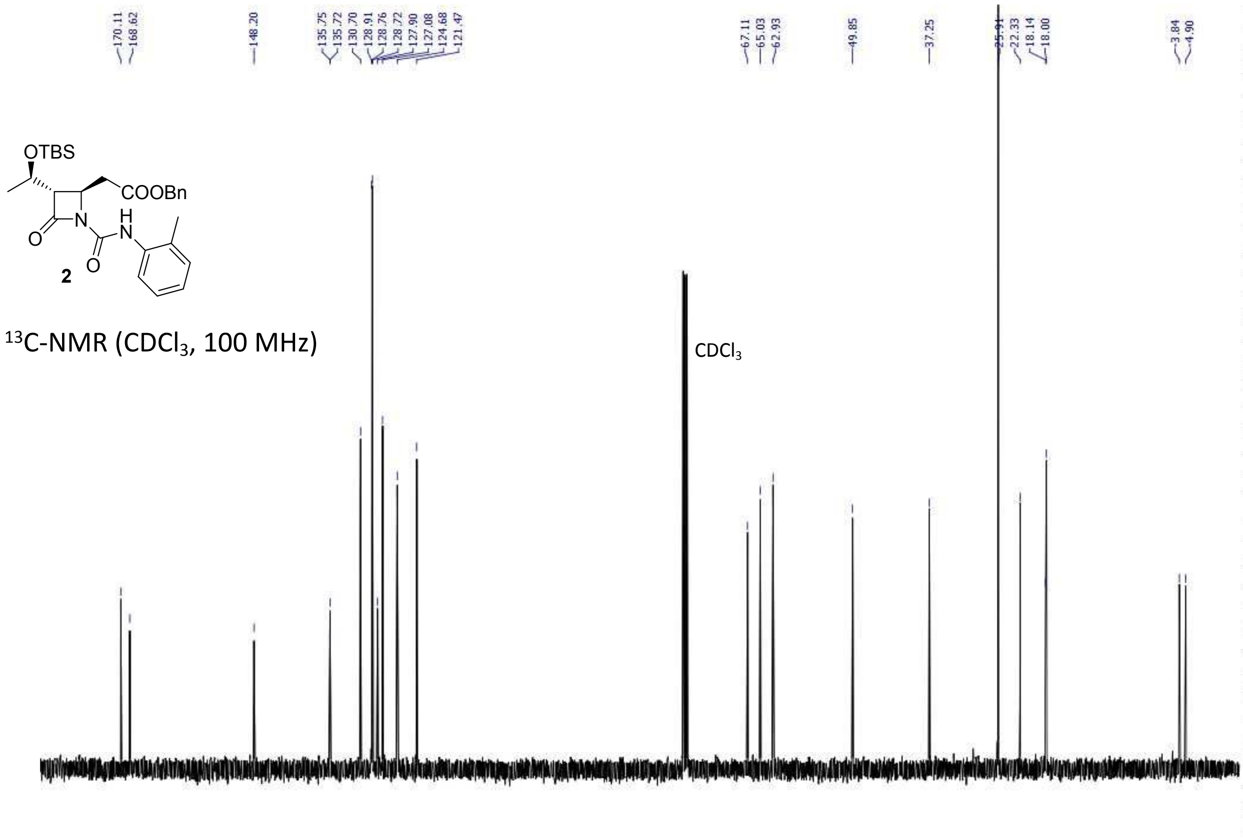

^{13}C-NMR ($CDCl_3$, 100 MHz)

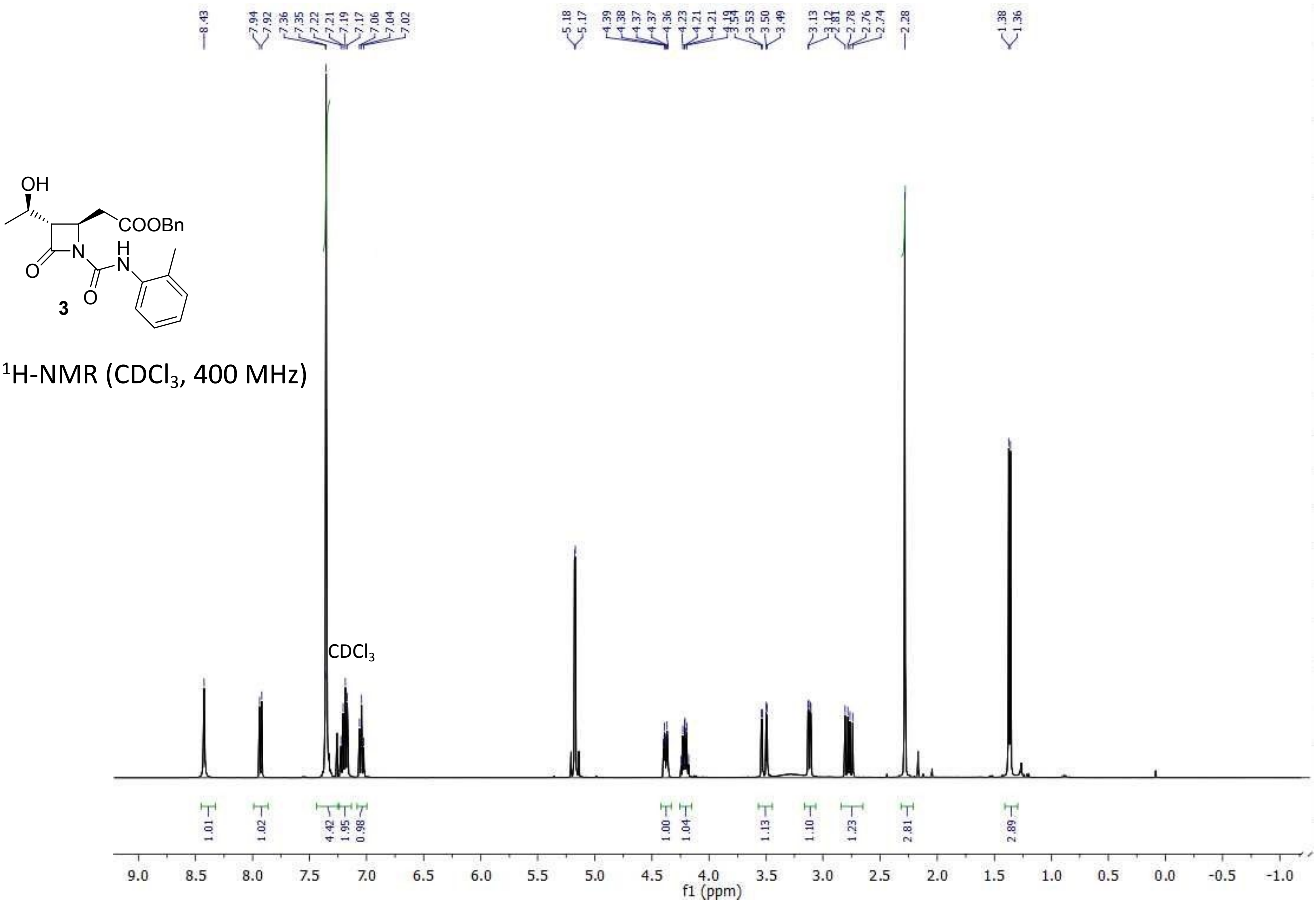

8.43
7.94
7.92
7.36
7.35
7.22
7.21
7.19
7.17
7.06
7.04
7.02
5.18
5.17
4.39
4.38
4.37
4.37
4.36
4.23
4.21
4.21
4.19
3.54
3.53
3.50
3.49
3.13
3.12
2.81
2.78
2.76
2.74
2.28
1.38
1.36
OH
COOBn
H
N
N
O
O
3
^{1}H-NMR (CDCl$_3$, 400 MHz)
CDCl$_3$
1.01
1.02
4.42
1.95
0.98
1.00
1.04
1.13
1.10
1.23
2.81
2.89
9.0 8.5 8.0 7.5 7.0 6.5 6.0 5.5 5.0 4.5 4.0 3.5 3.0 2.5 2.0 1.5 1.0 0.5 0.0 -0.5 -1.0
f1 (ppm)

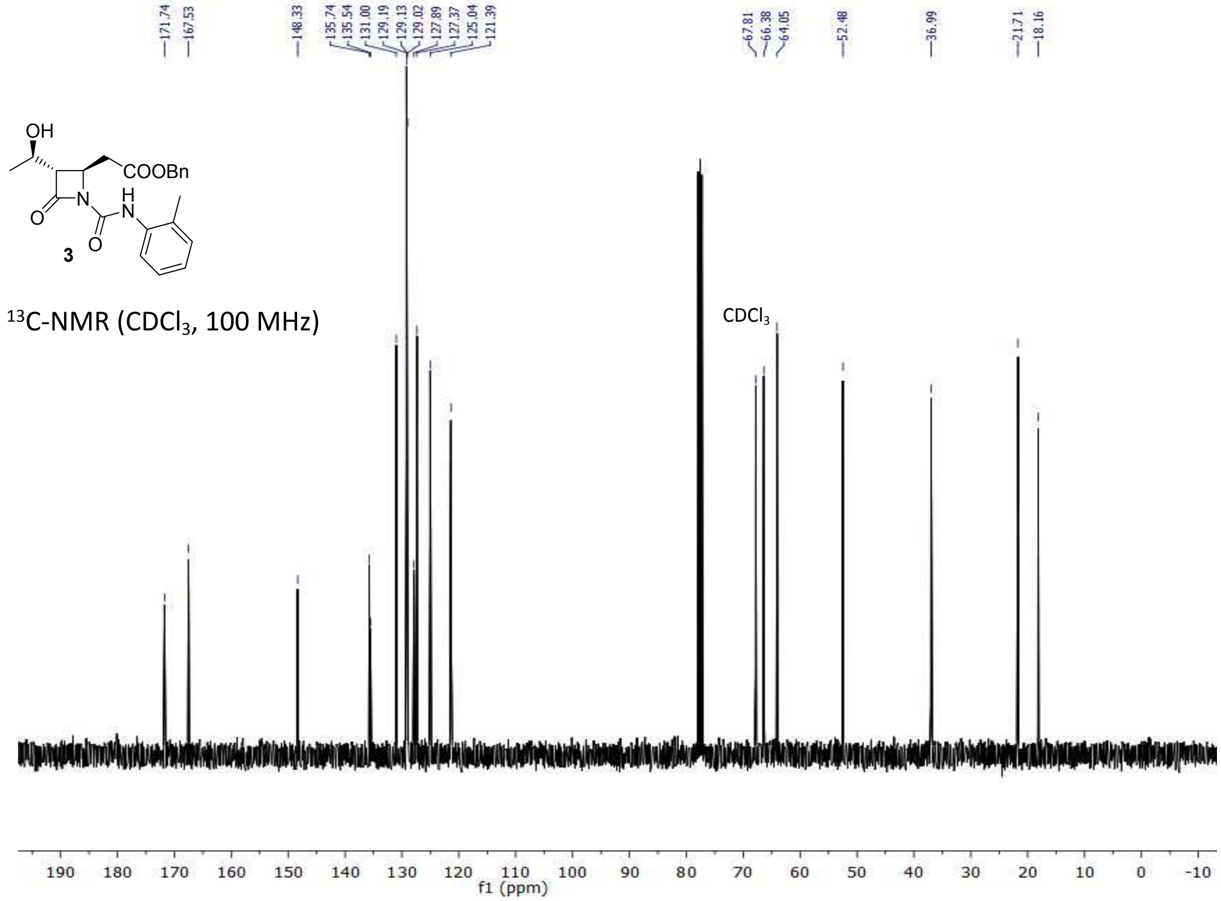

171.74
167.53
148.33
135.74
135.54
131.00
129.19
129.13
129.02
127.89
127.37
125.04
121.39
67.81
66.38
64.05
52.48
36.99
21.71
18.16
OH
COOBn
H
N
N
O
O
3
^{13}C-NMR (CDCl$_3$, 100 MHz)
CDCl$_3$
190 180 170 160 150 140 130 120 110 100 90 80 70 60 50 40 30 20 10 0 -10
f1 (ppm)

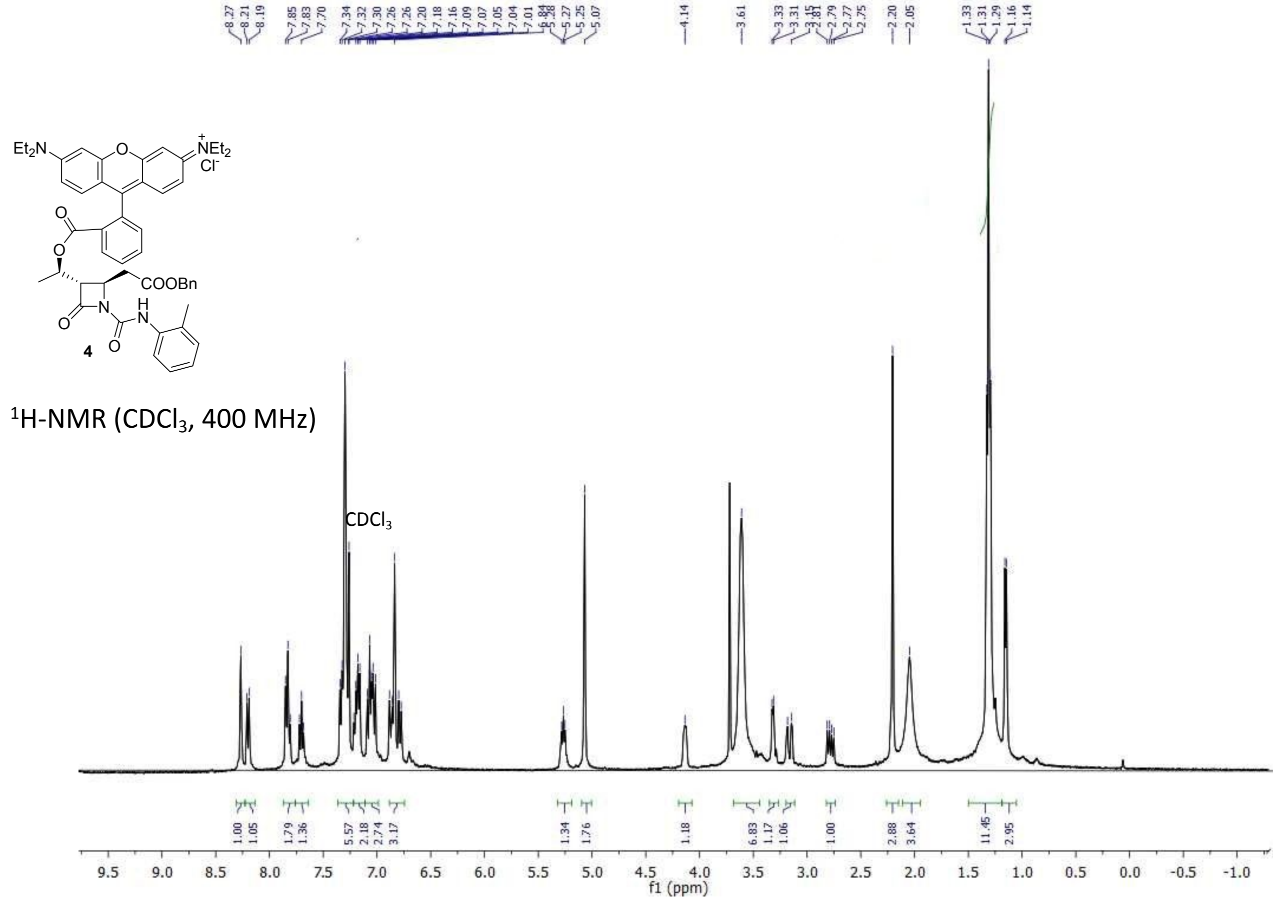

^{1}H-NMR ($CDCl_3$, 400 MHz)

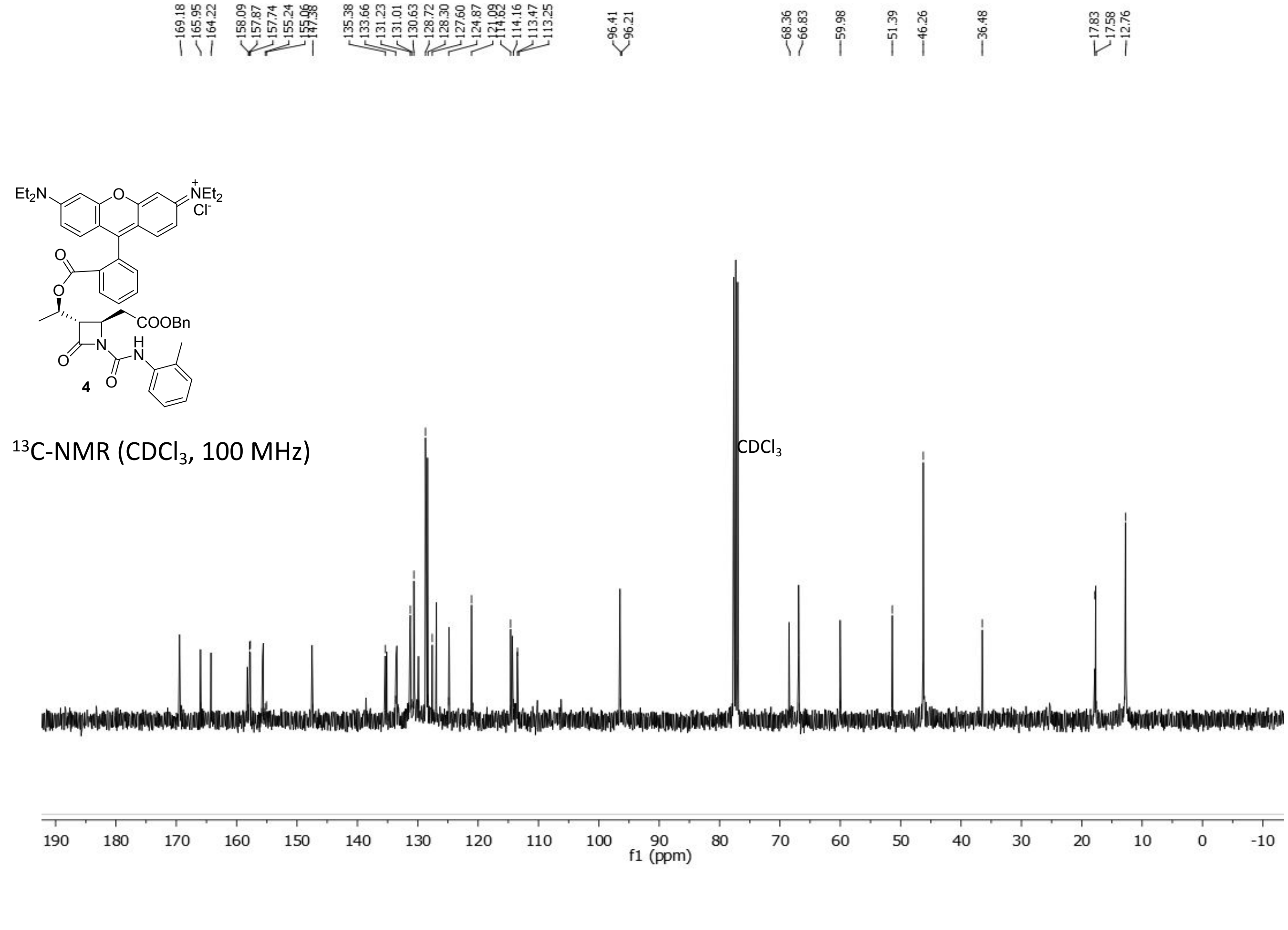

^{13}C-NMR ($CDCl_3$, 100 MHz)

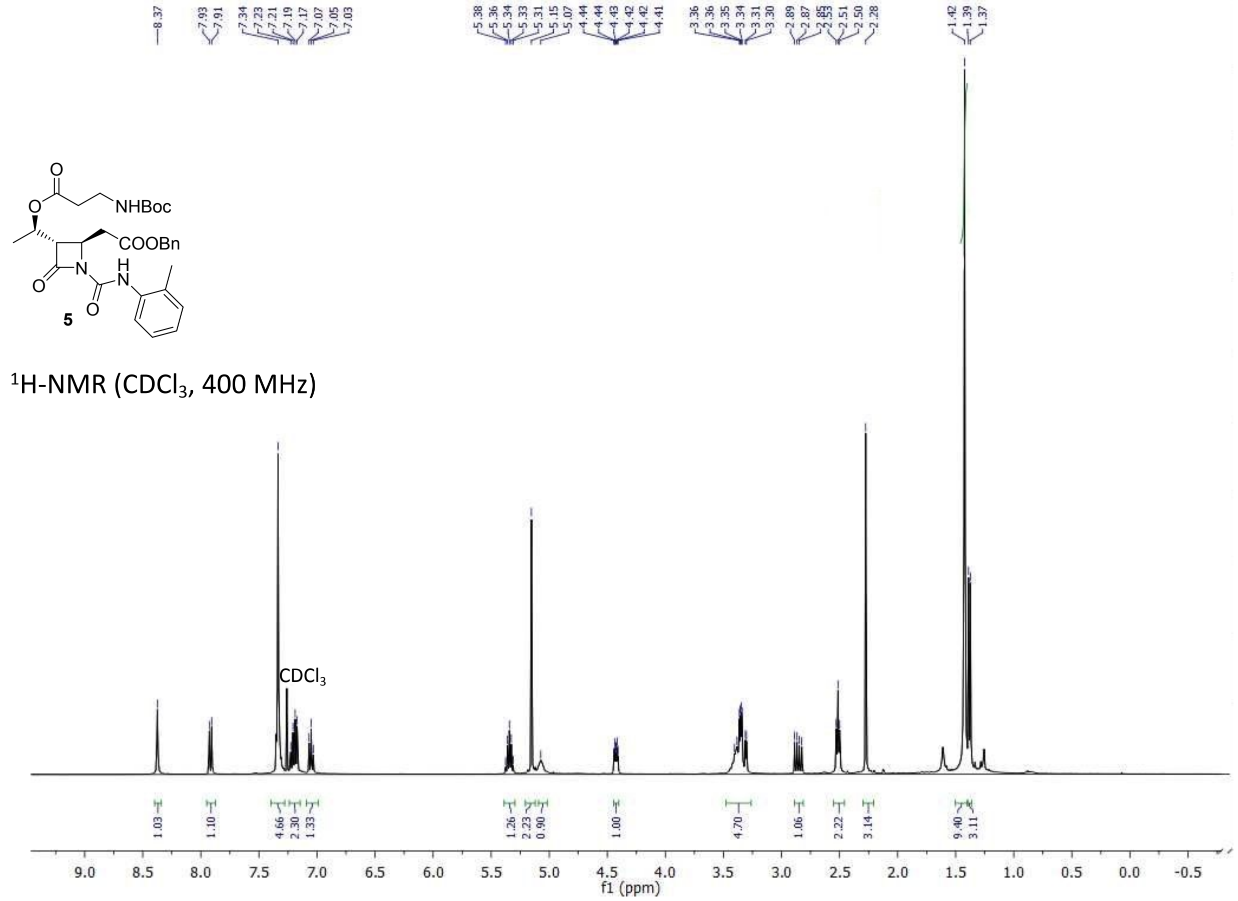

^{1}H-NMR ($CDCl_3$, 400 MHz)

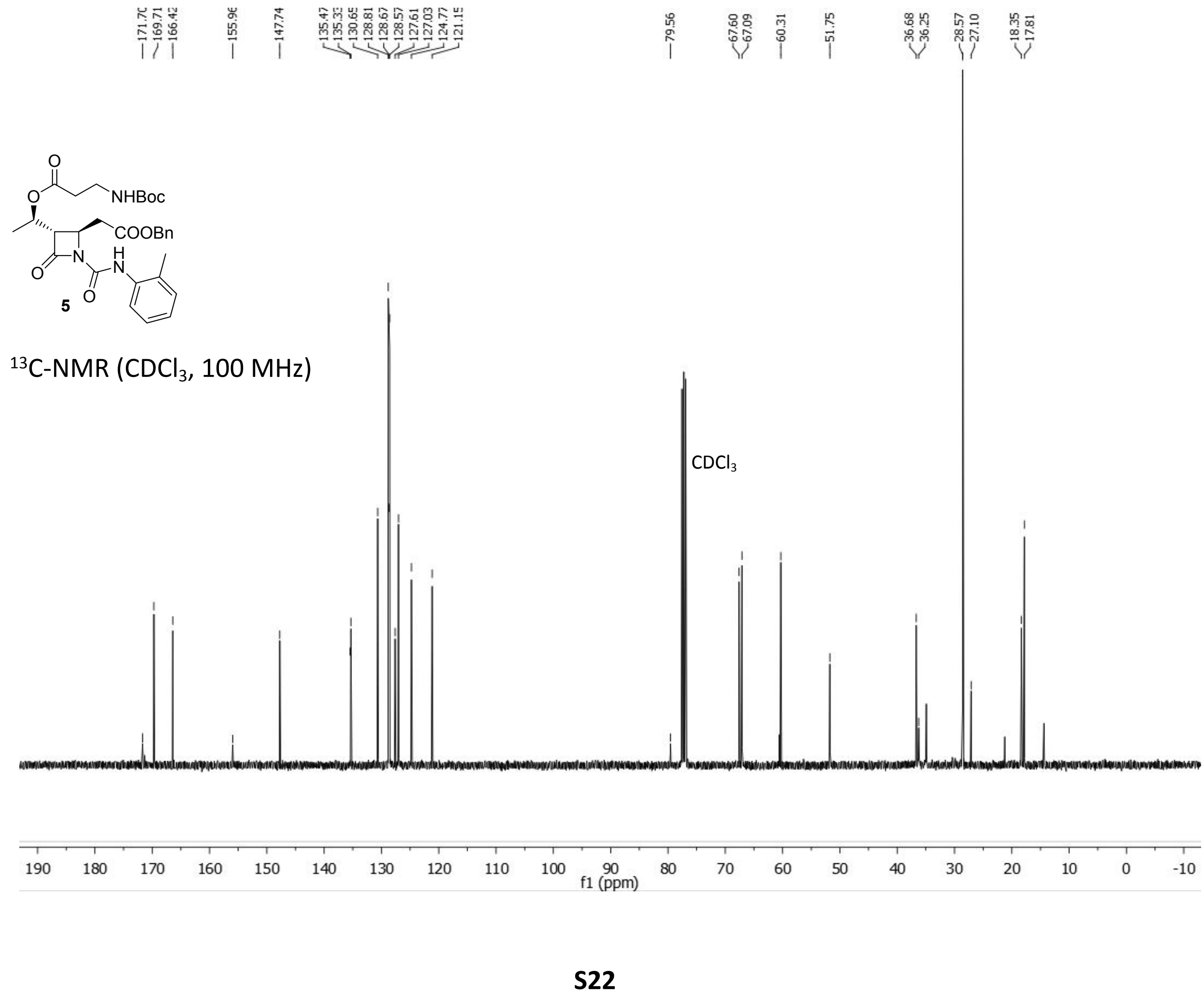

^{13}C-NMR ($CDCl_3$, 100 MHz)

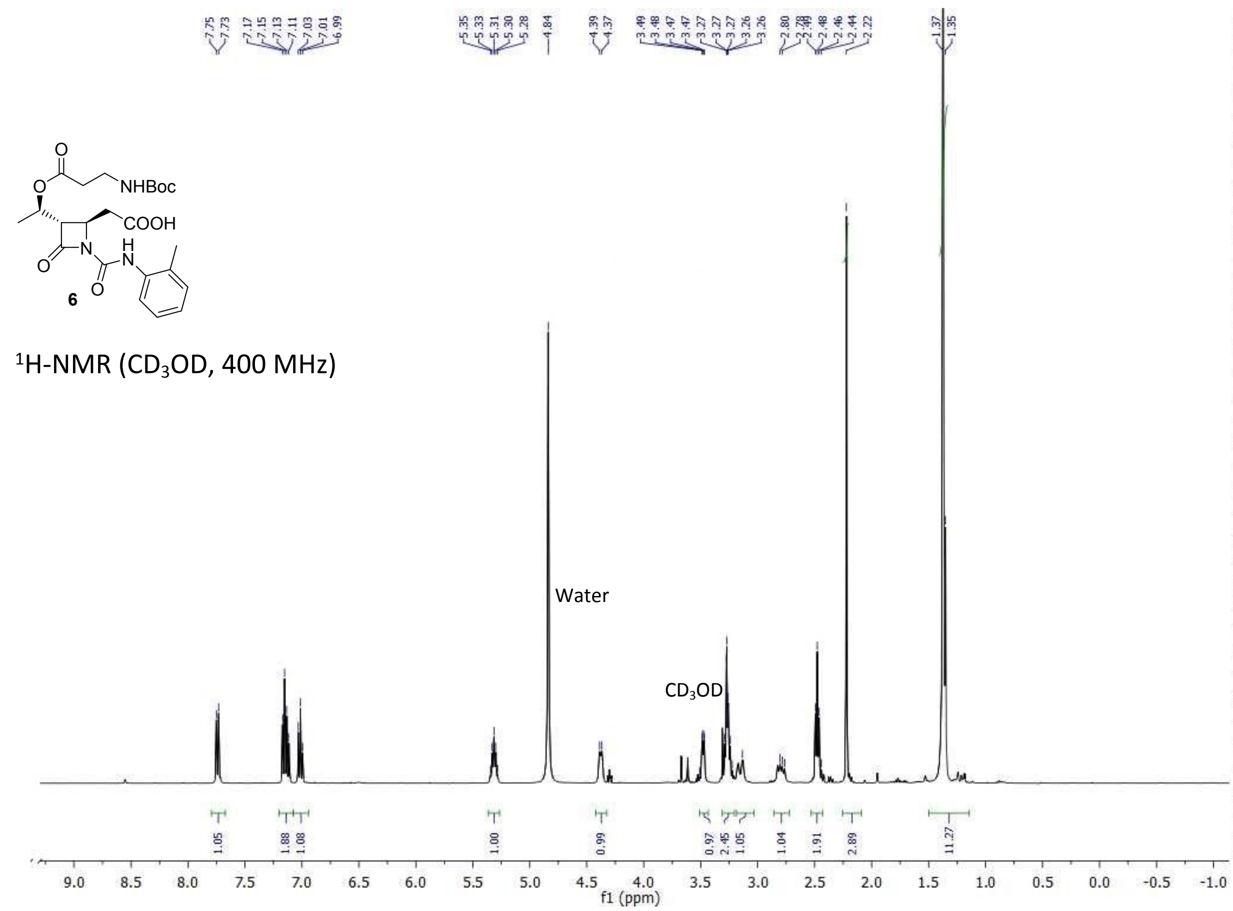

^{1}H-NMR (CD_3OD, 400 MHz)

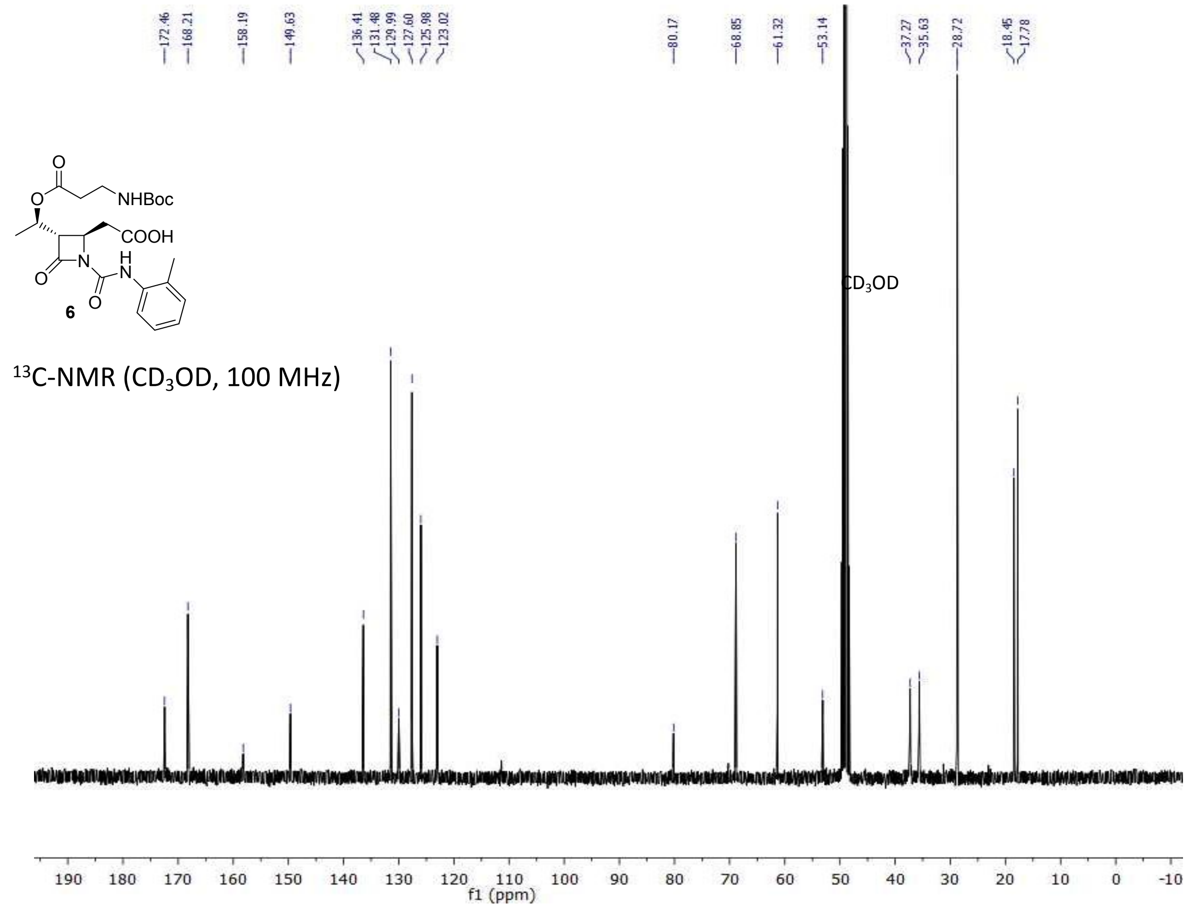

^{13}C-NMR (CD_3OD, 100 MHz)

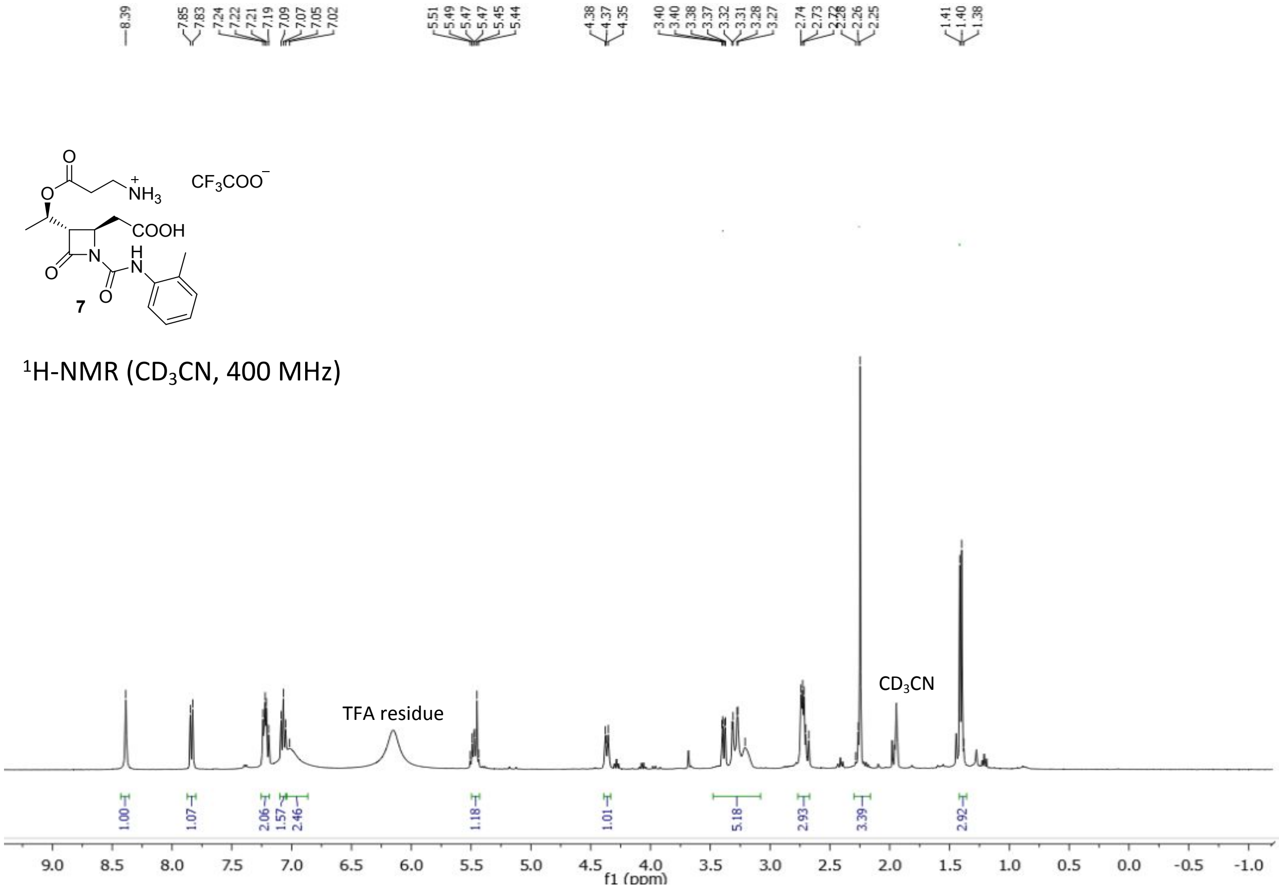
8.39
7.85 7.83 7.24 7.22 7.21 7.19 7.09 7.07 7.05 7.02
5.51 5.49 5.47 5.47 5.45 5.44
4.38 4.37 4.35
3.40 3.40 3.38 3.37 3.32 3.31 3.28 3.27
2.74 2.73 2.72 2.28 2.26 2.25
1.41 1.40 1.38
CF$_3$COO$^-$
COOH
7
^{1}H-NMR (CD$_3$CN, 400 MHz)
TFA residue
CD$_3$CN
1.00 1.07 2.06 1.57 2.46 1.18 1.01 5.18 2.93 3.39 2.92
f1 (ppm)

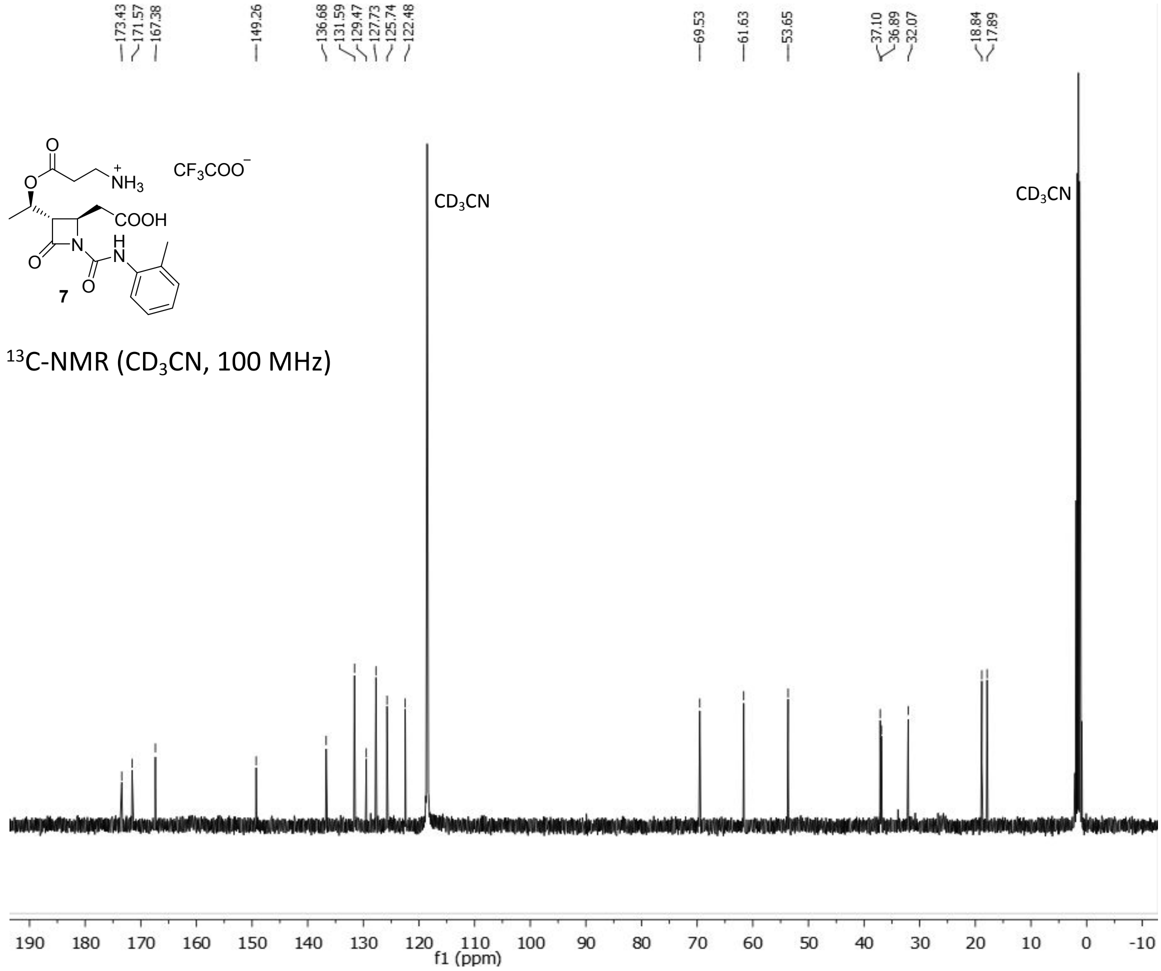
173.43 171.57 167.38
149.26
136.68 131.59 129.47 127.73 125.74 122.48
69.53
61.63
53.65
37.10 36.89 32.07
18.84 17.89
CF$_3$COO$^-$
COOH
7
^{13}C-NMR (CD$_3$CN, 100 MHz)
CD$_3$CN
CD$_3$CN
f1 (ppm)

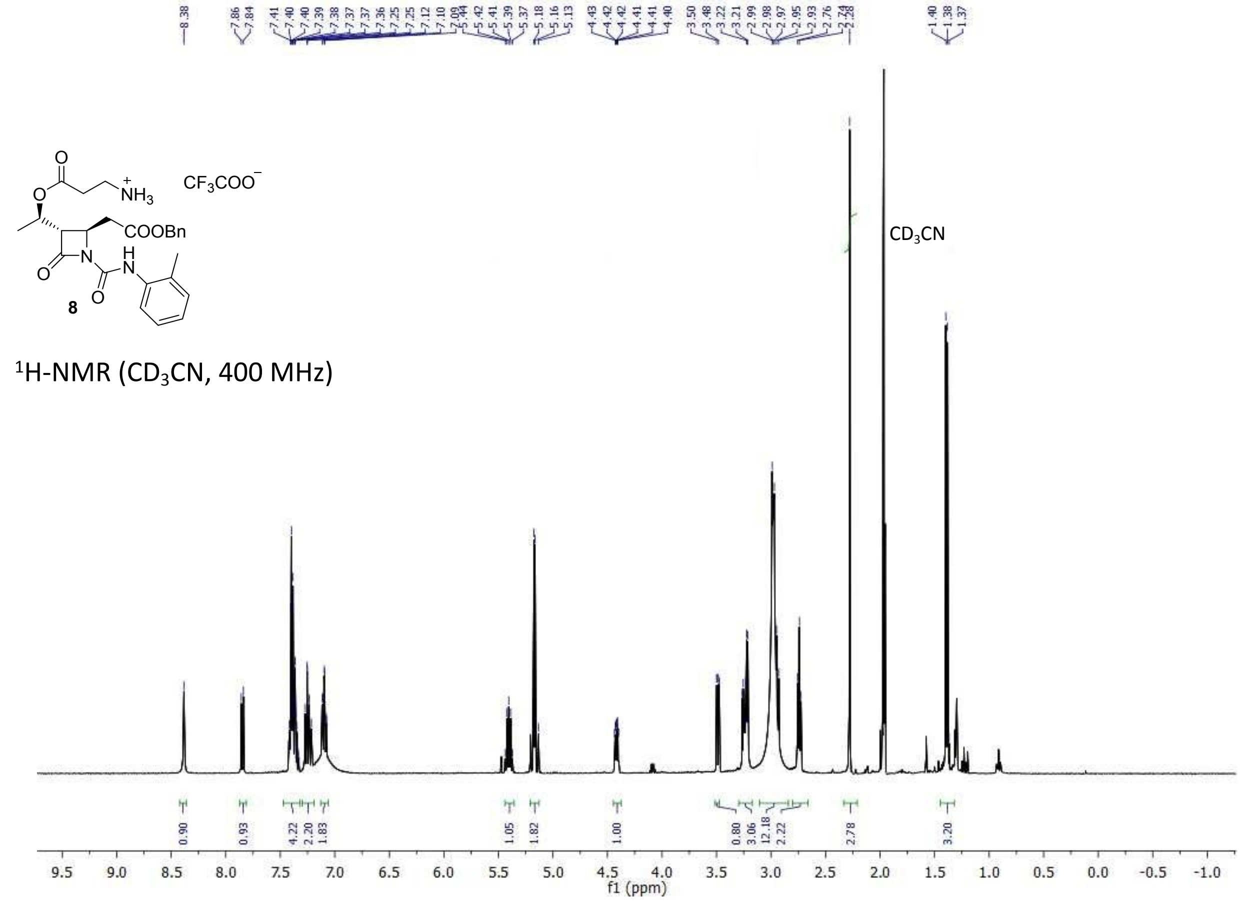

^{1}H-NMR (CD_3CN, 400 MHz)

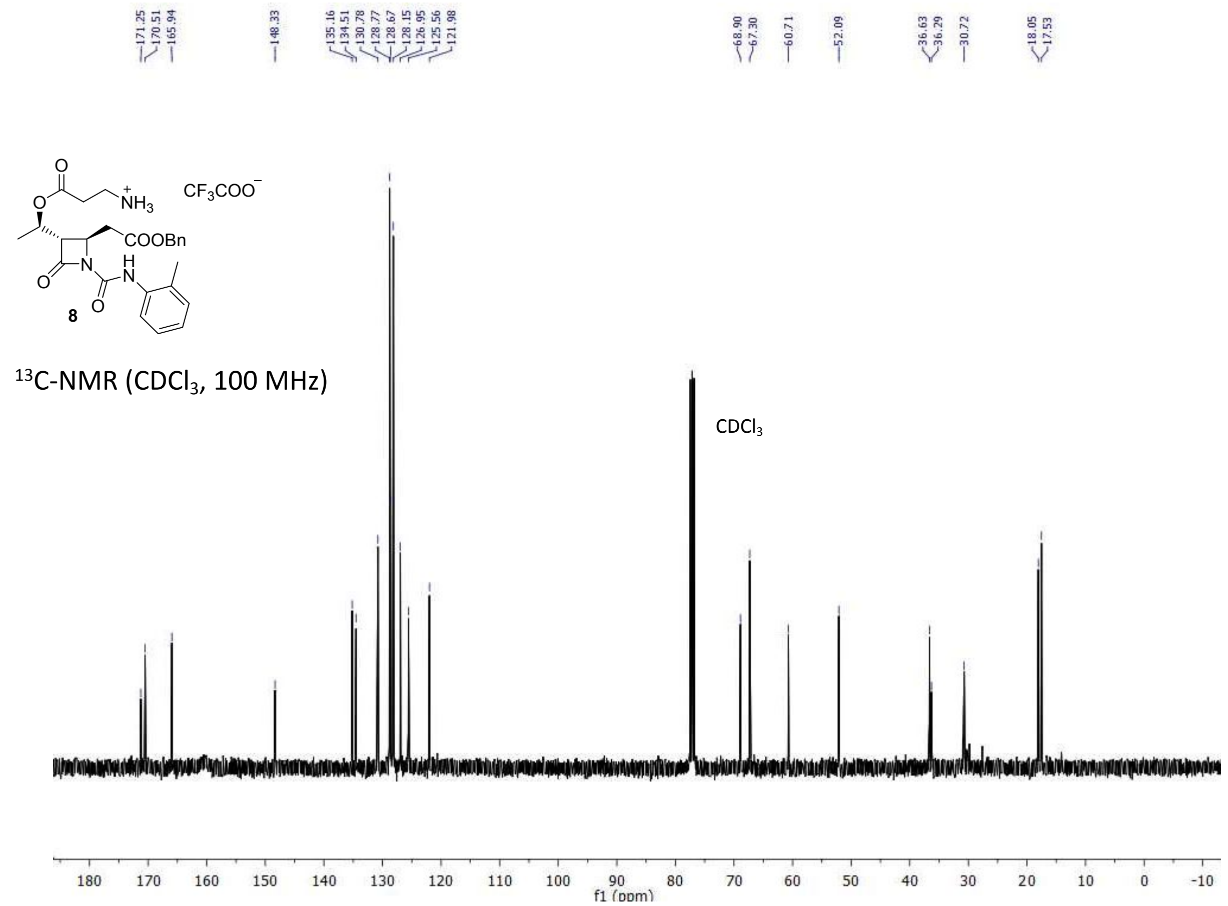

^{13}C-NMR ($CDCl_3$, 100 MHz)

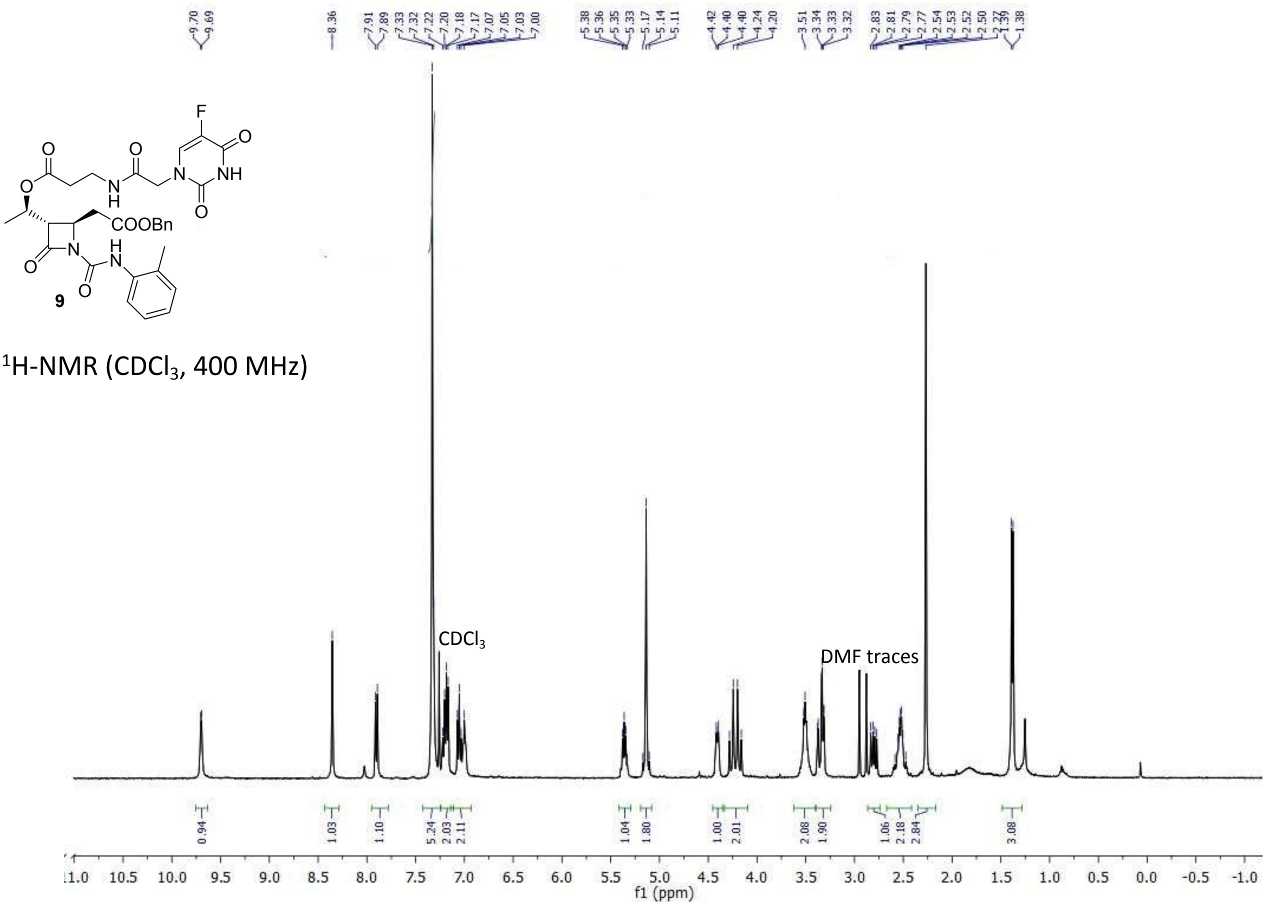

^{1}H-NMR ($CDCl_3$, 400 MHz)

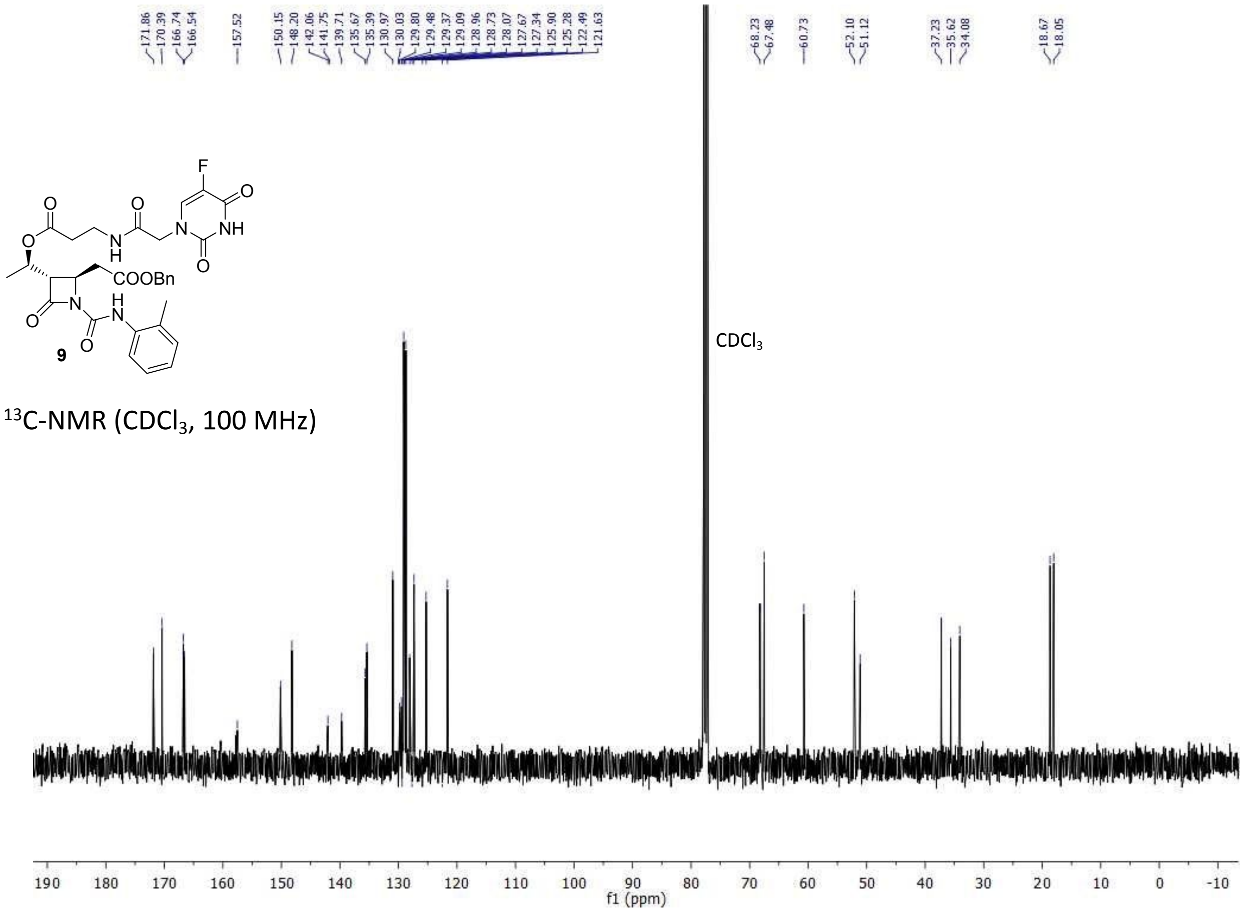

^{13}C-NMR ($CDCl_3$, 100 MHz)

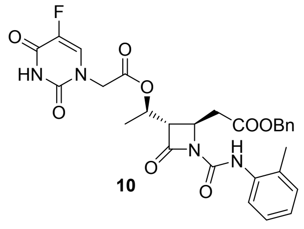

10

^{1}H-NMR ($CDCl_3$, 400 MHz)

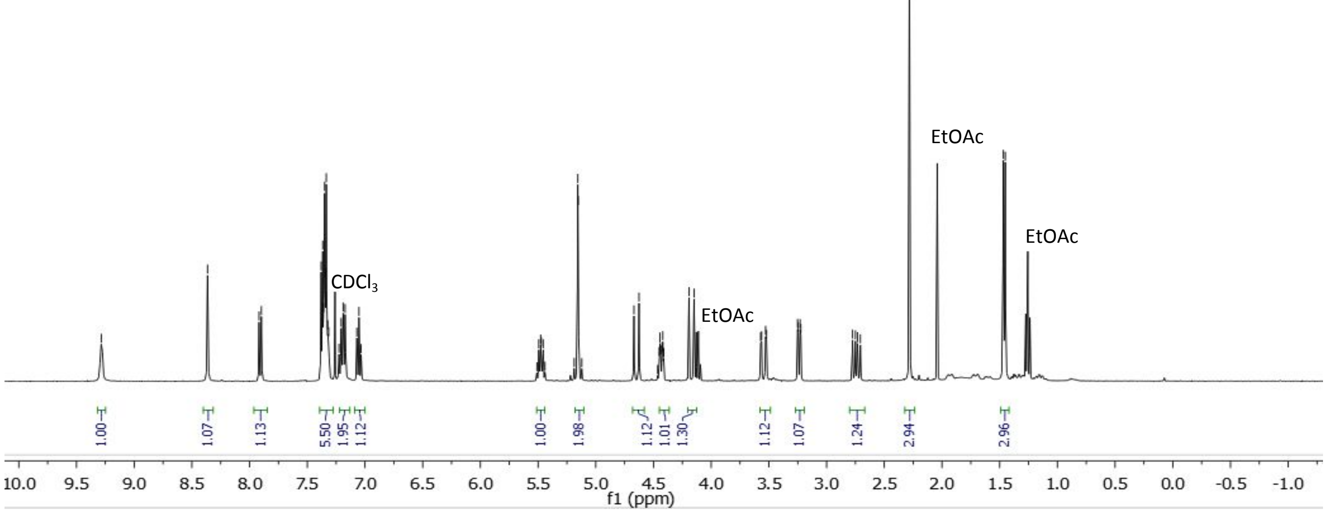

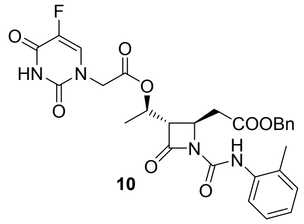

10

^{13}C-NMR ($CDCl_3$, 100 MHz)

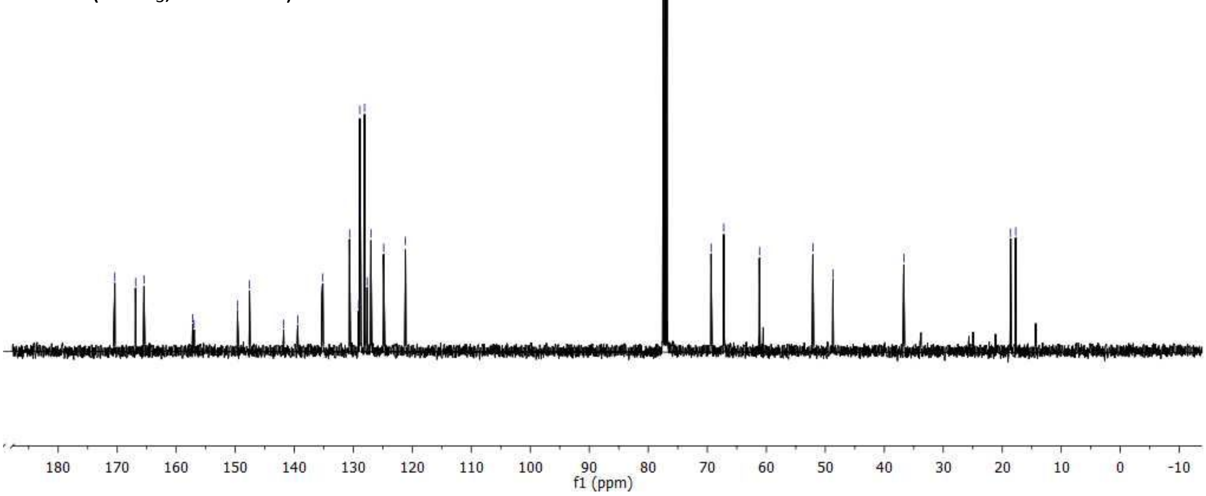

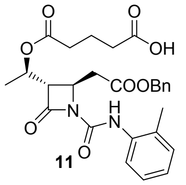

11

^{1}H-NMR ($CDCl_3$, 400 MHz)

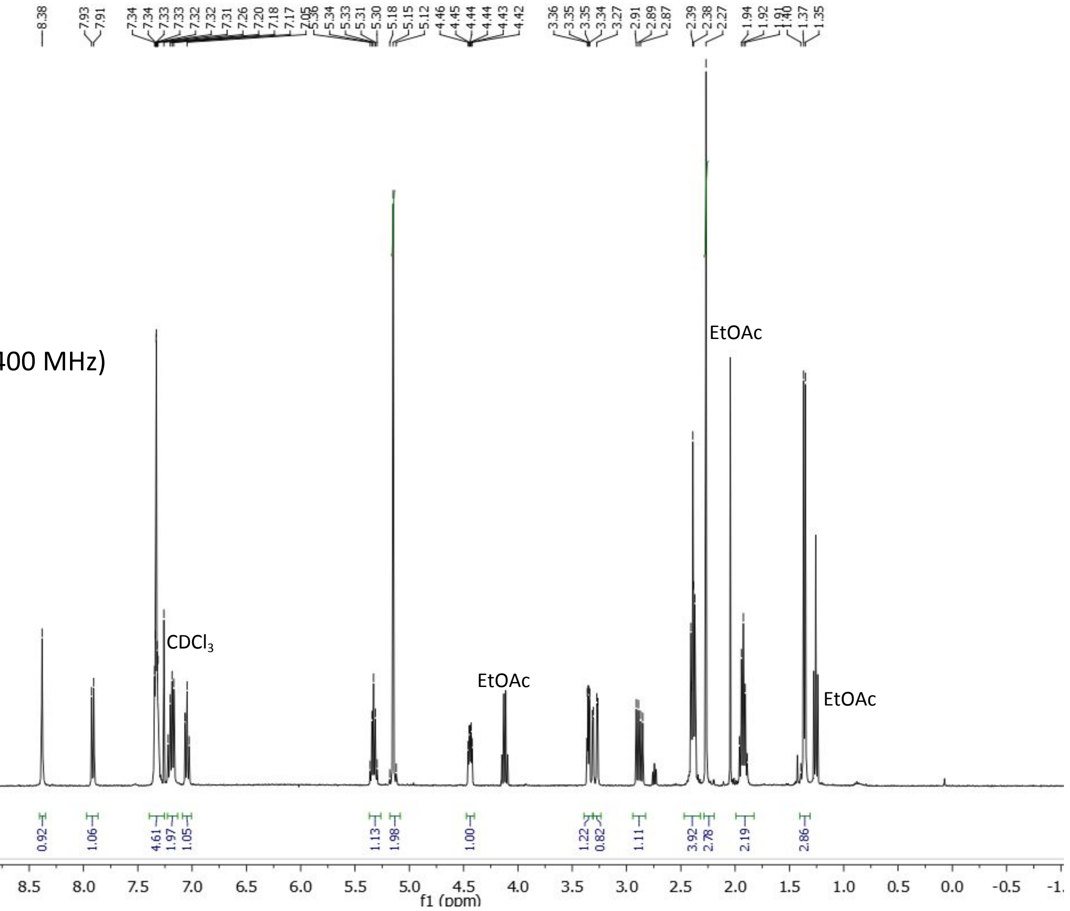

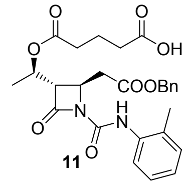

11

^{13}C-NMR ($CDCl_3$, 100 MHz)

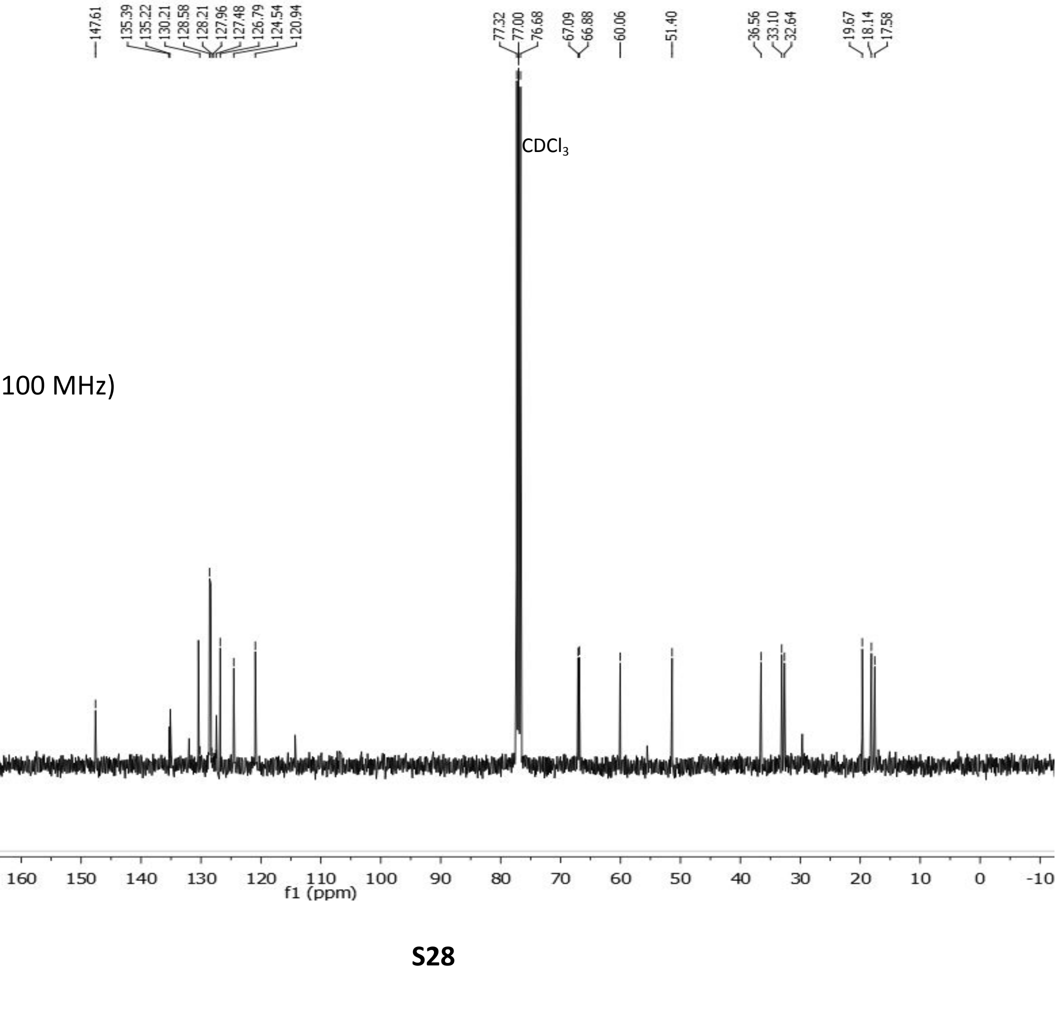

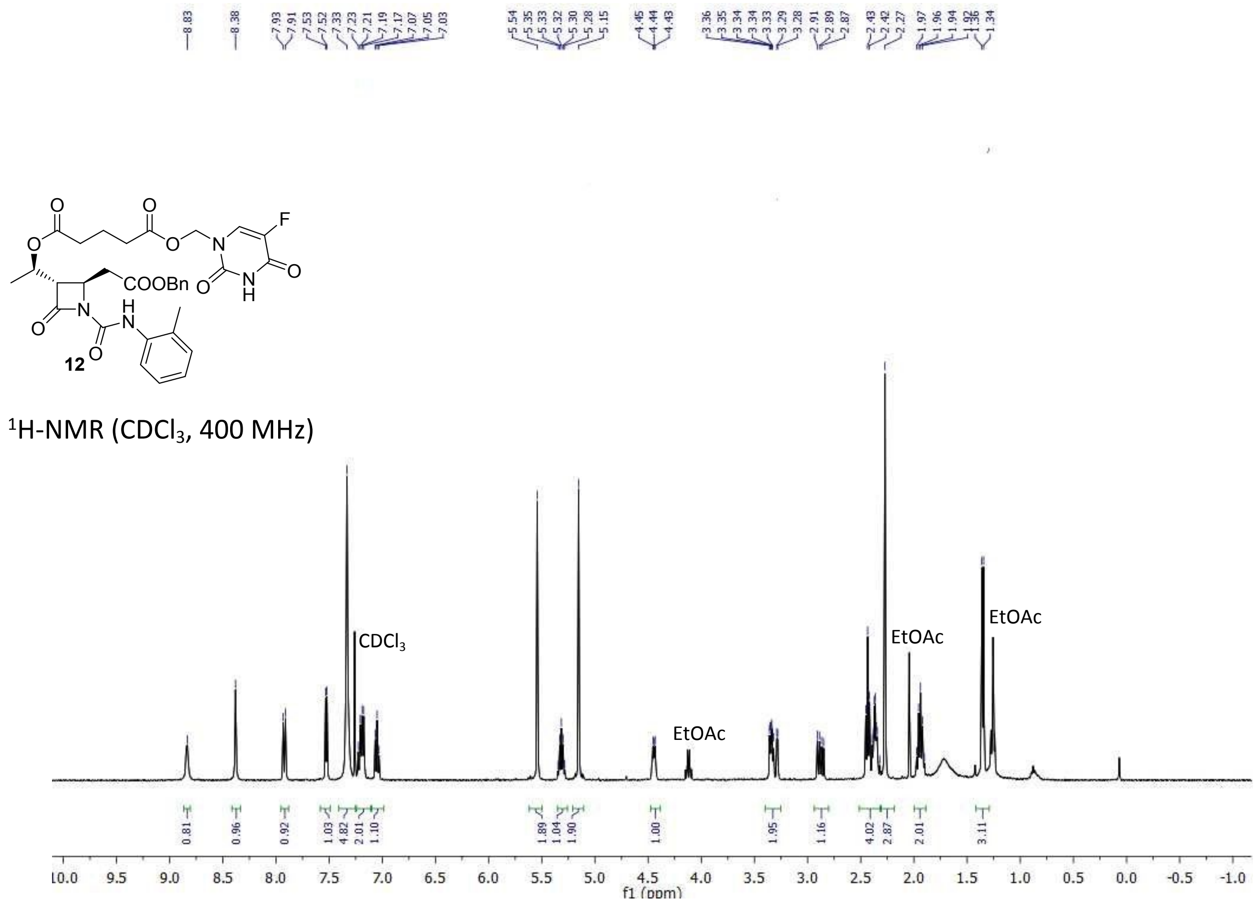

^{1}H-NMR (CDCl$_3$, 400 MHz)

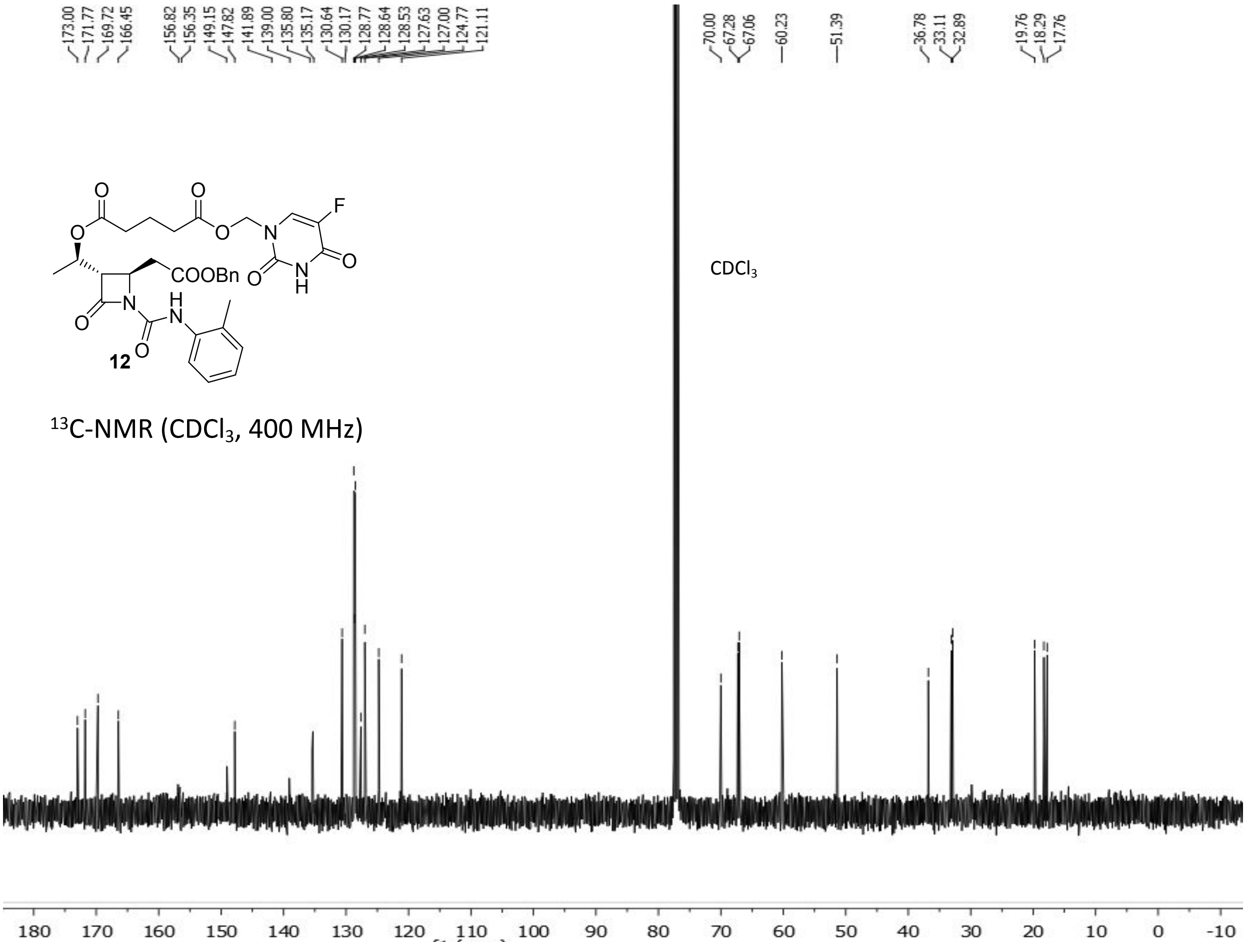

^{13}C-NMR (CDCl$_3$, 400 MHz)

HPLC analysis of compounds B, C, D, E, and F

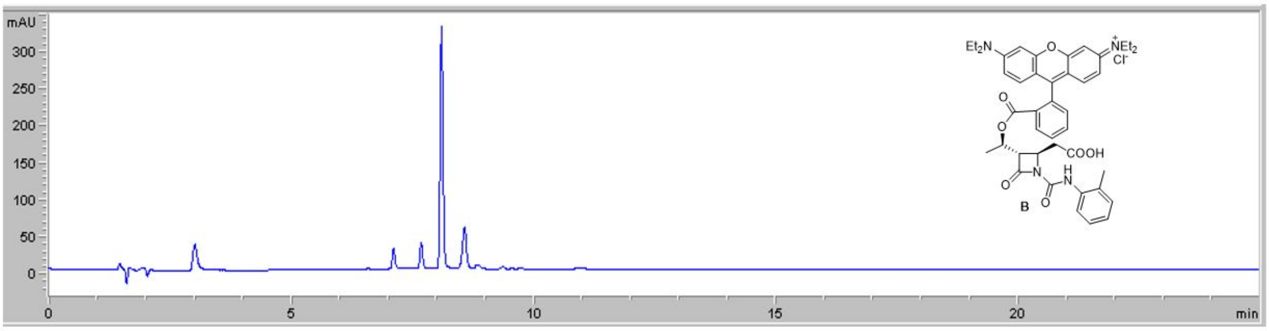

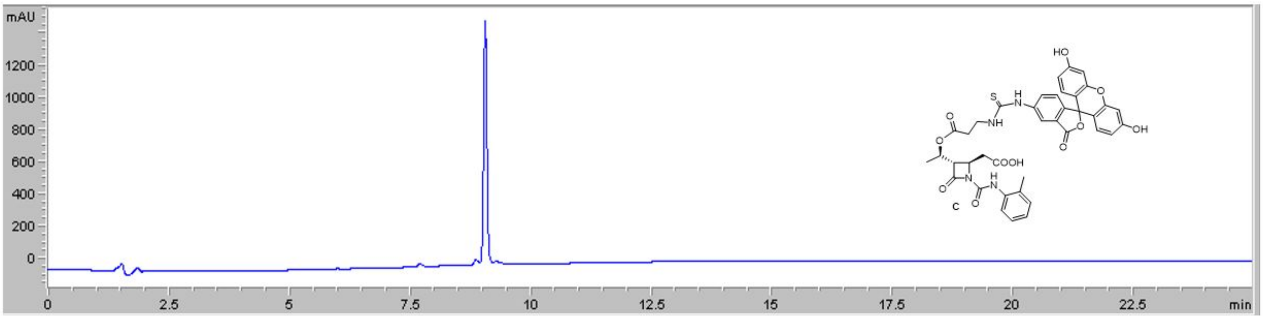

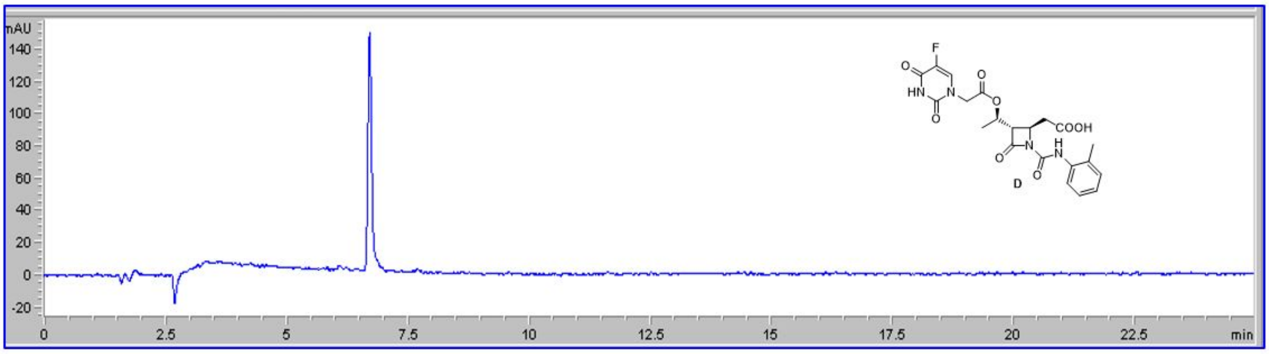

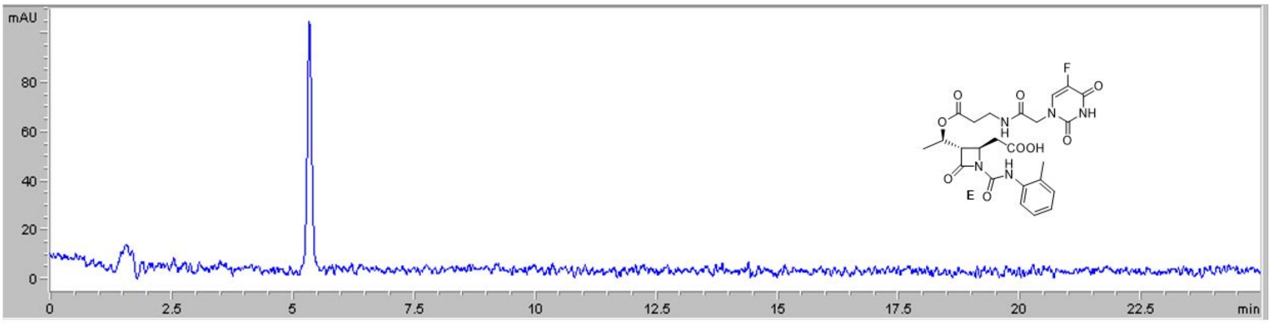

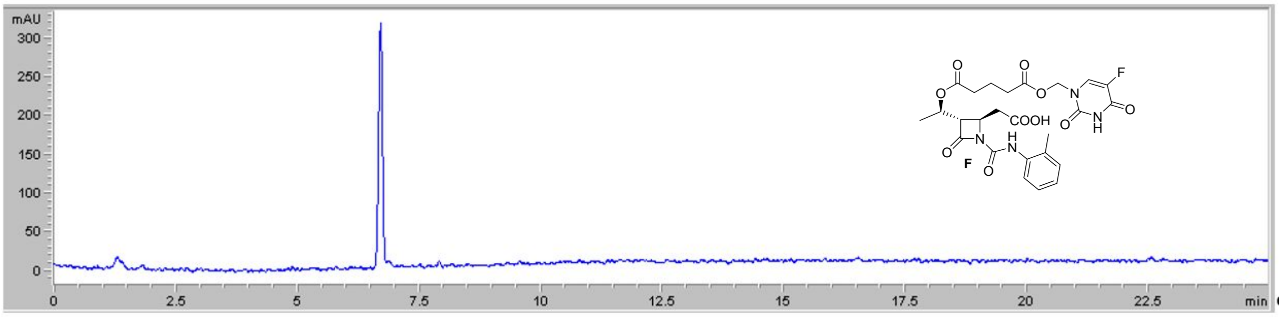

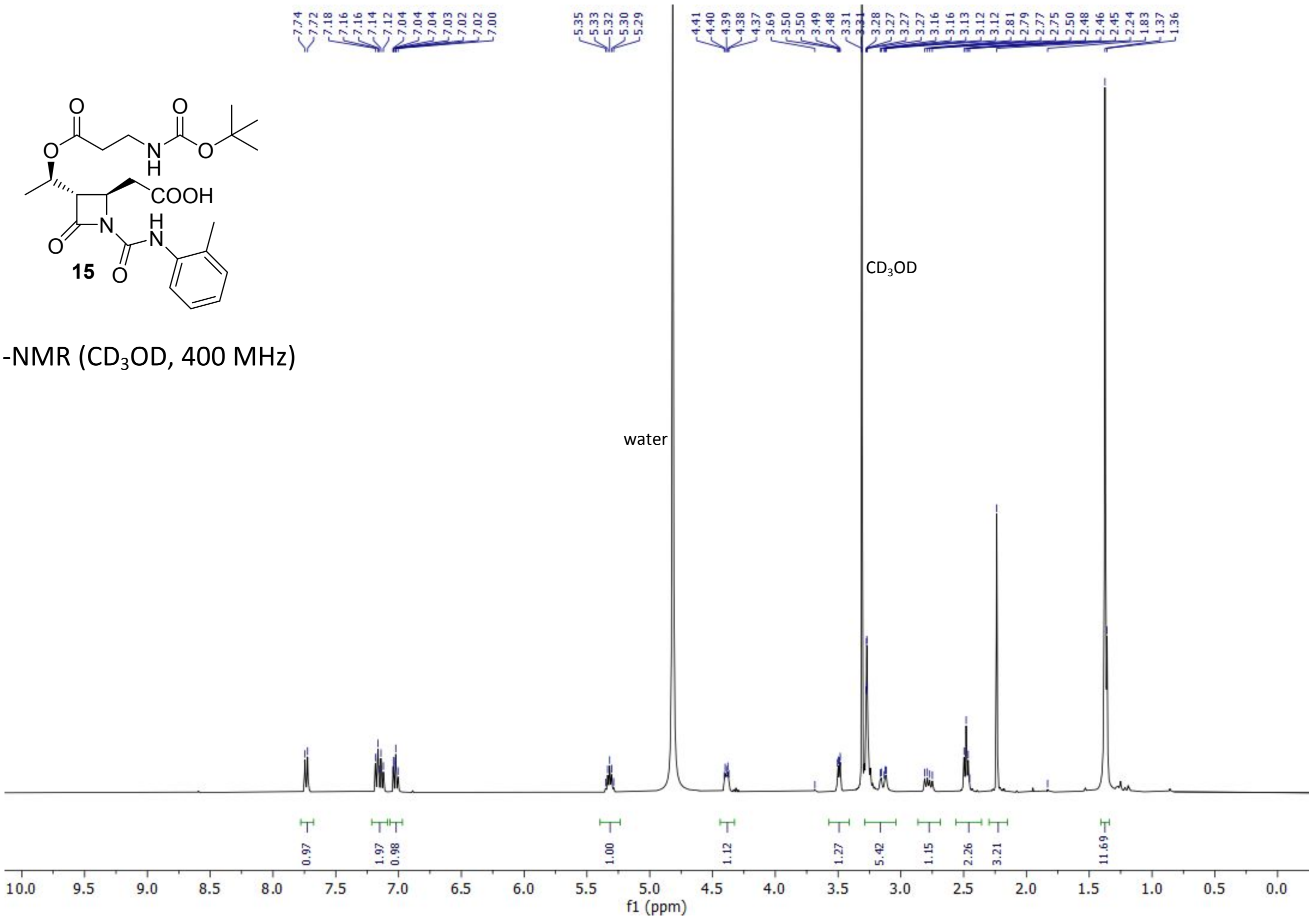

^{1}H-NMR (CD_3OD, 400 MHz)

HPLC-MS analysis of compound **15**

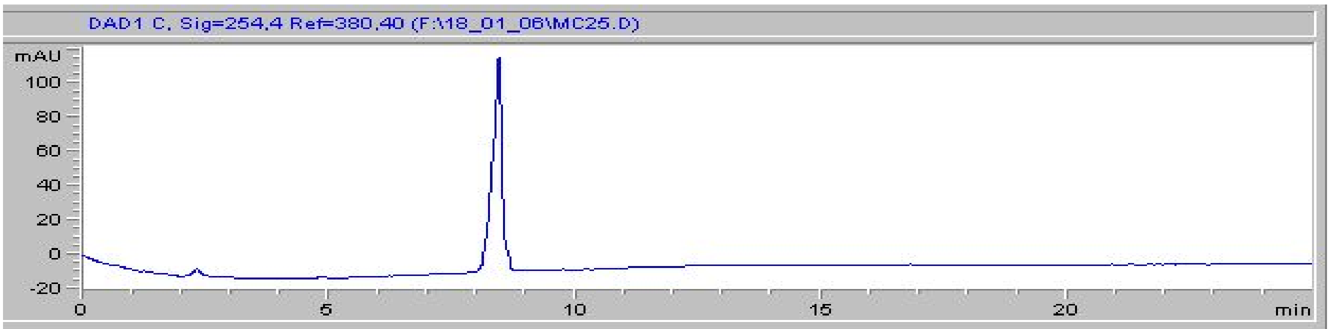

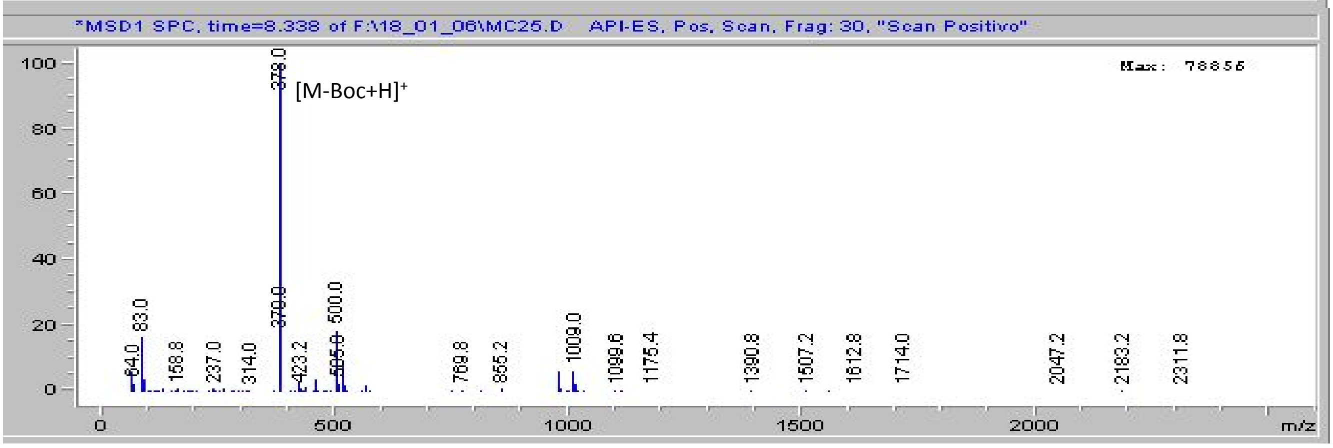

Supplement: Supplementary file 1 — pt1c00094_si_001.pdf [file pt1c00094_si_001.pdf]
